# Supplementary material for: Synthesis, Cytotoxic Activity Evaluation and Quantitative Structure-ActivityAnalysis of Substituted 5,8-Dihydroxy-1,4-naphthoquinones and Their O- and S-Glycoside Derivatives Tested against Neuro-2a Cancer Cells
Source: Mar Drugs. 2020 Nov 29;18(12):602. doi: 10.3390/md18120602 (PMC7761386; doi:10.3390/md18120602)

Supplementary Materials

for the paper

"Synthesis, Cytotoxic Activity Evaluation and Quantitative Structure-Activity Analysis of Substituted 5,8-Dihydroxy-1,4-Naphthoquinones and their *O*- and *S*-Glycoside Derivatives Tested Against Neuro-2a Cancer Cells"

Sergey Polonik,Galina Likhatskaya, Yuri Sabutski, Dmitry Pelageev, Vladimir Denisenko, Evgeny Pislyagin, Ekaterina Chingizova, Ekaterina Menchinskaya, Dmitry Aminin.

Content

^{1}H and ^{13}C NMR spectra for synthesized compounds S2-S34

2-(Tetra-O-acetyl-β-D-glucopyranosyl-1-oxy)-5,8-dihydroxy-3-methylnaphthalene-1,4-dione ***18 (****U-444),* *(*[1]*H NMR - 500 MHz,* [13]*C NMR - 125 MHz, solvent - CDCl*$_3$*)*

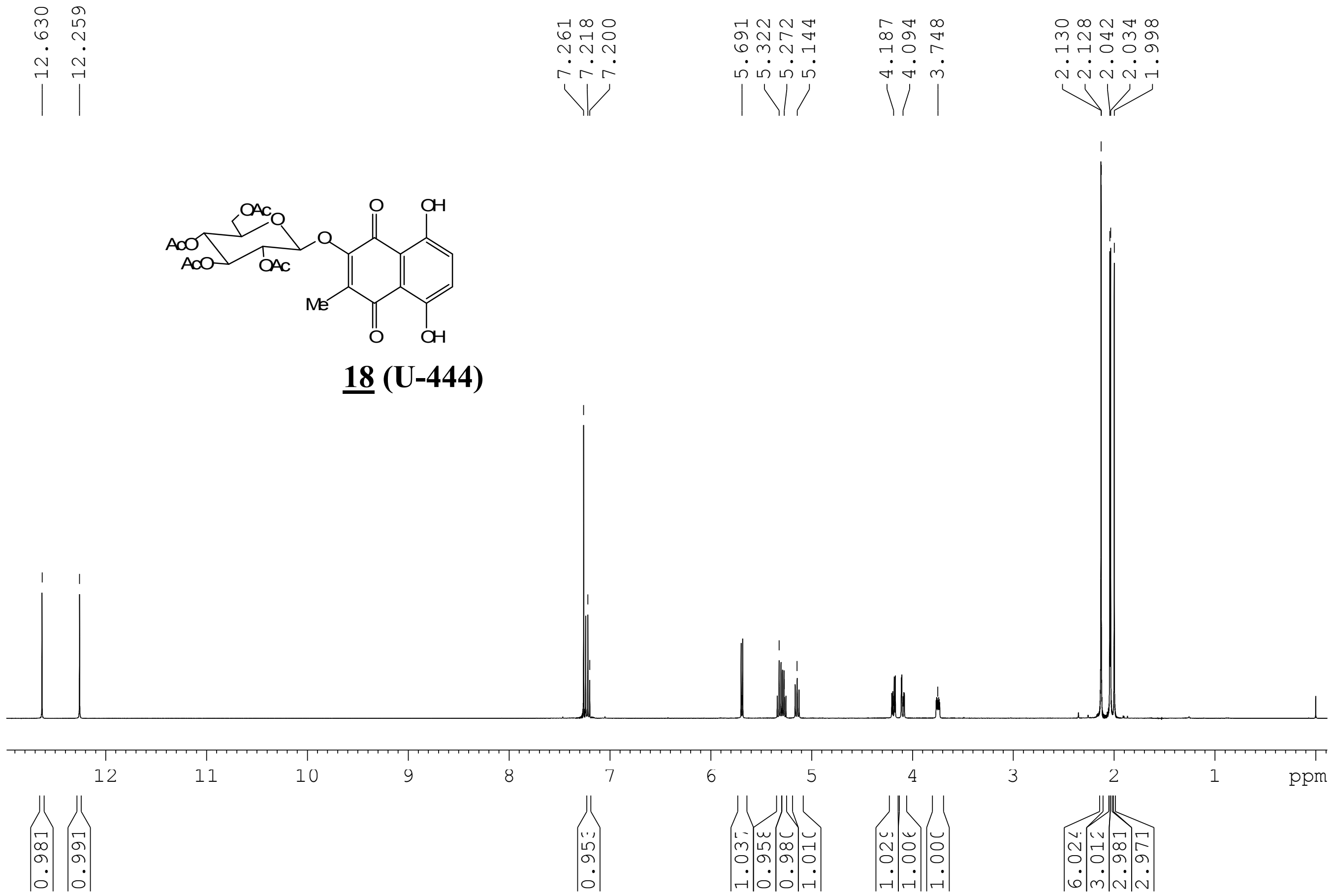

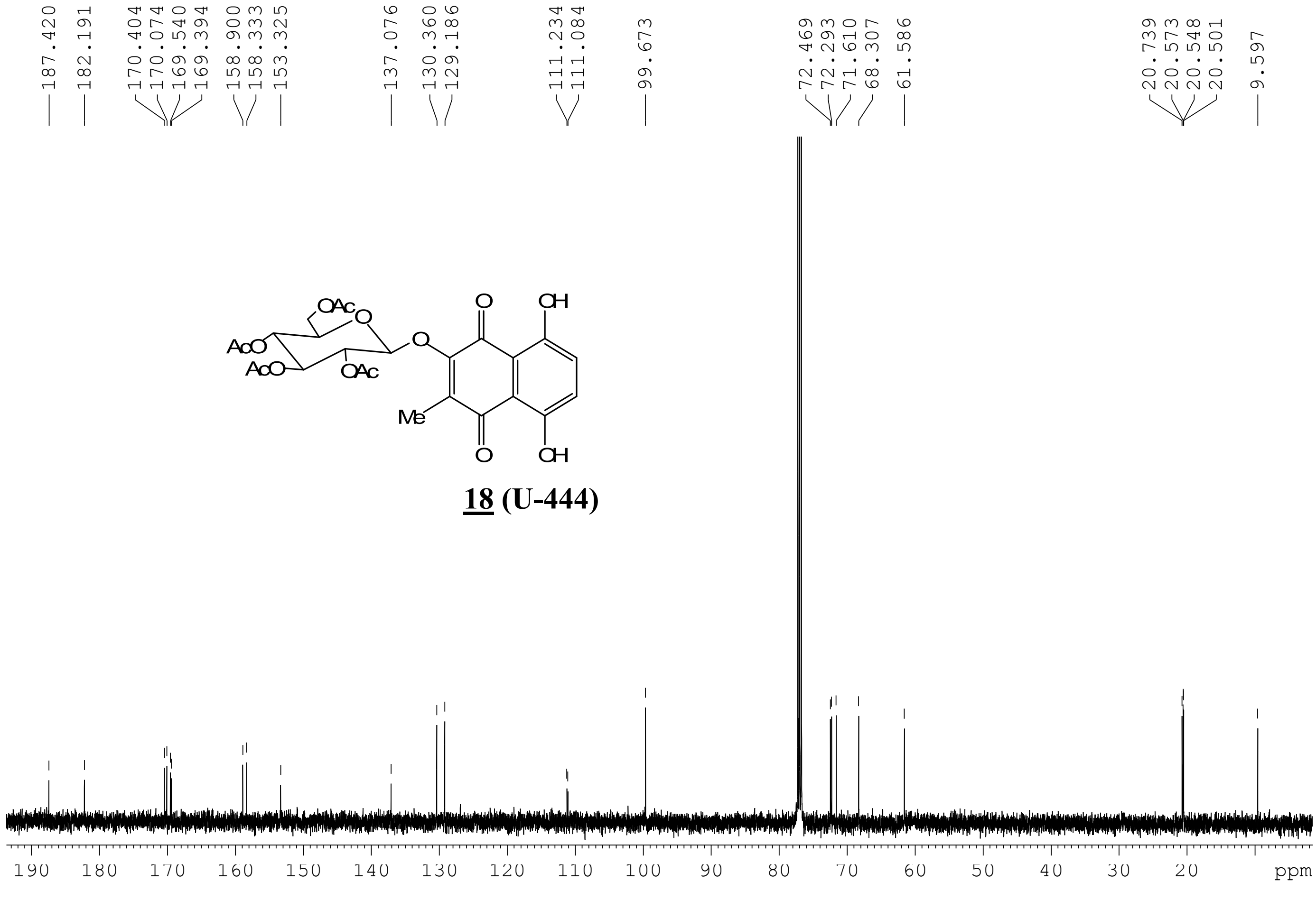

2-(Tetra-O-acetyl-β-D-glucopyranosyl-1-oxy)-3-ethyl-5,8-dihydroxynaphthalene-1,4-dione ***19*** *(U-443)****,*** *(^{1}H NMR - 500 MHz, ^{13}C NMR - 125 MHz, solvent - CDCl$_3$)*

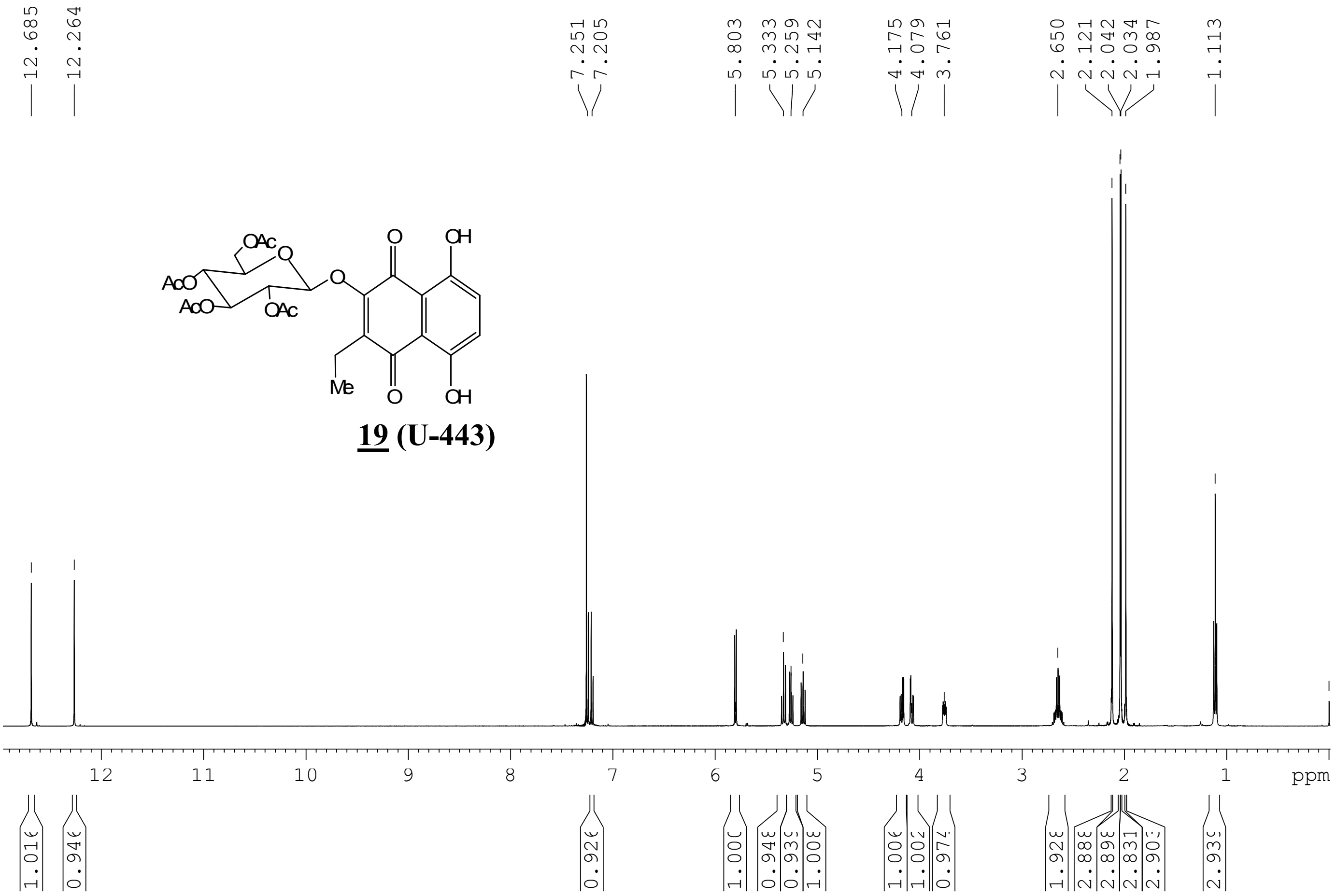

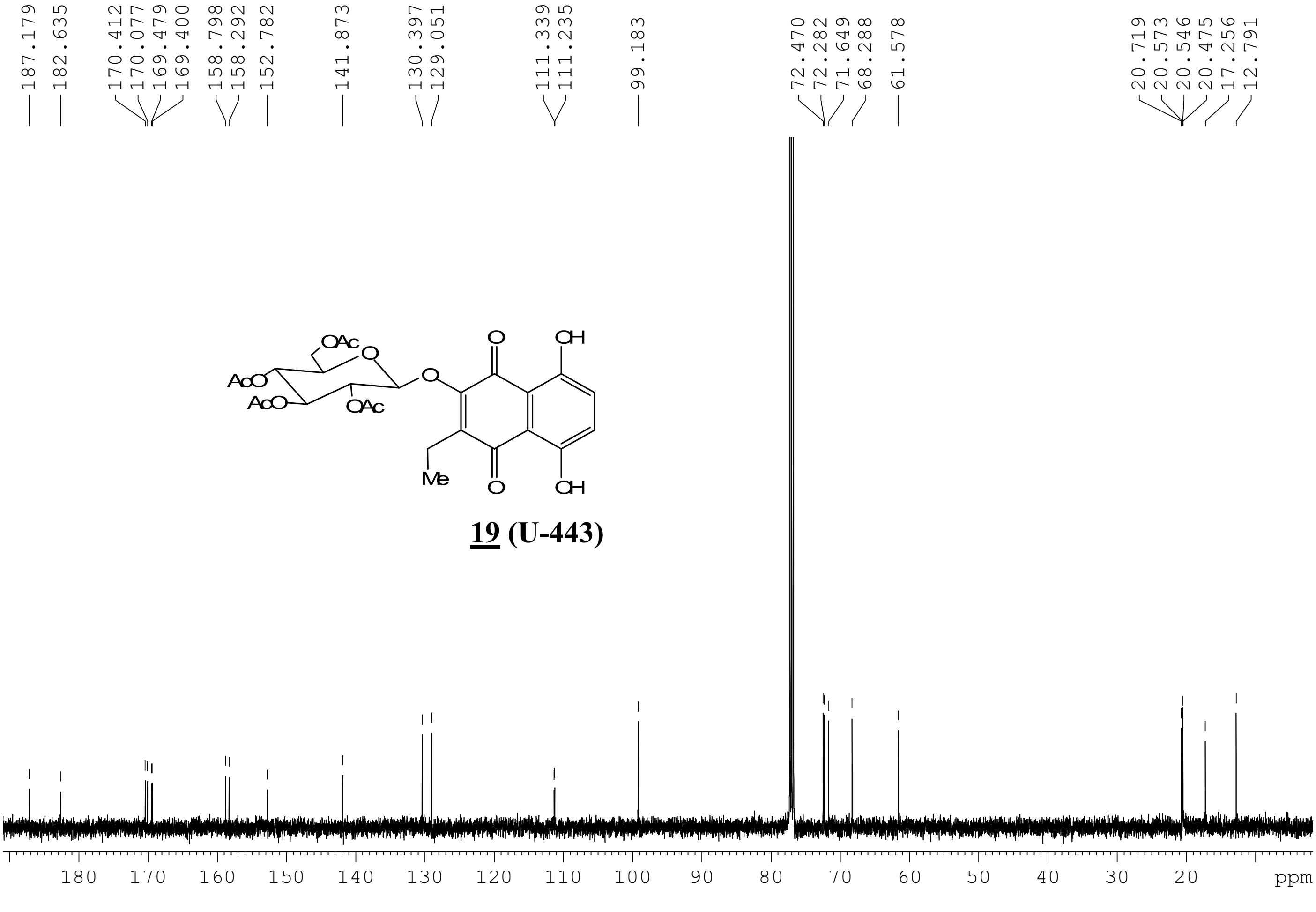

2,3-Bis(tetra-O-acetyl-β-D-glucopyranosyl-1-oxy)-5,8- dihydroxy-6-methylnaphthalene-1,4-dione ***20*** *(U-420)* ***,*** *(^{1}H NMR - 500 MHz, ^{13}C NMR - 125 MHz, solvent - $CDCl_3$)*

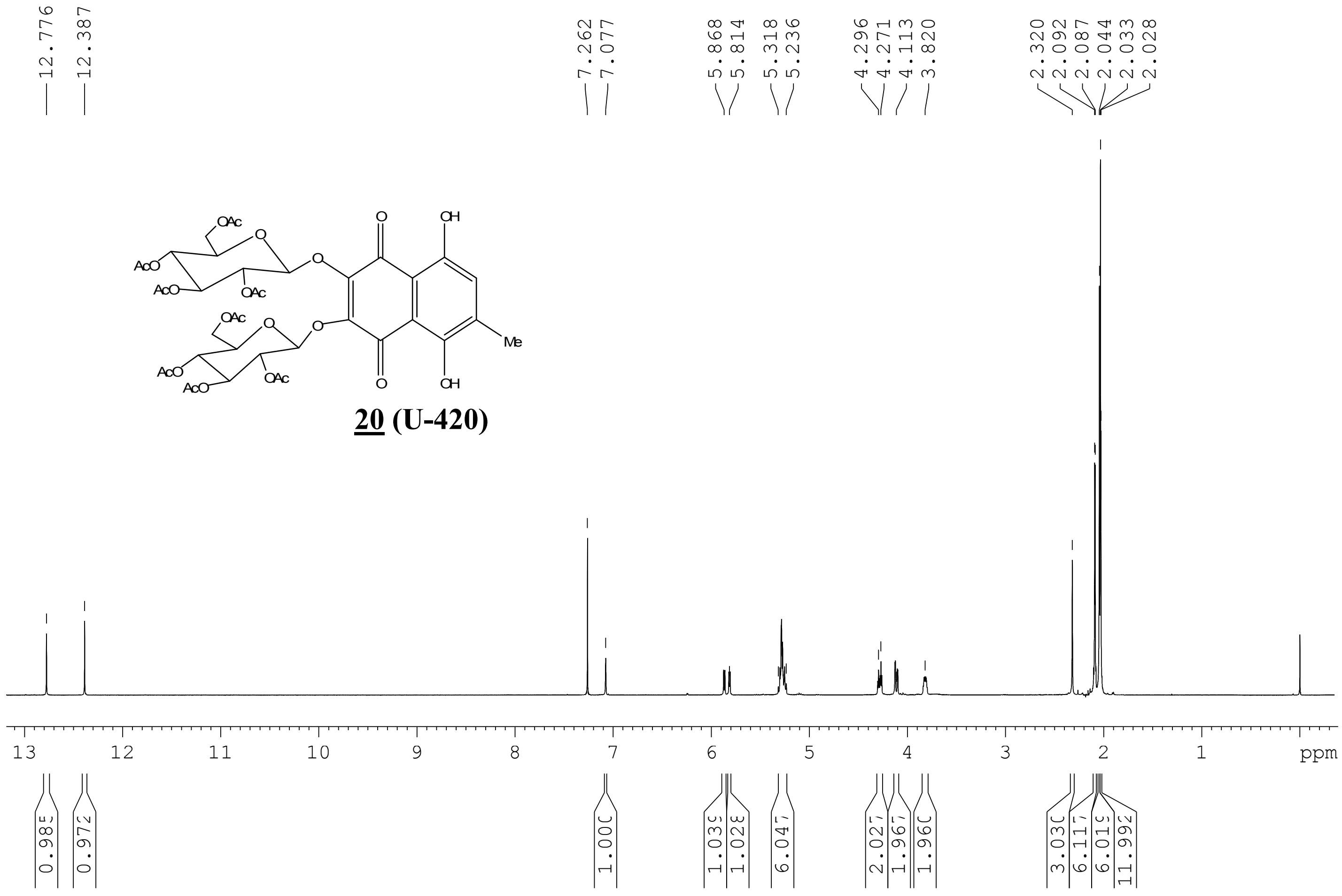

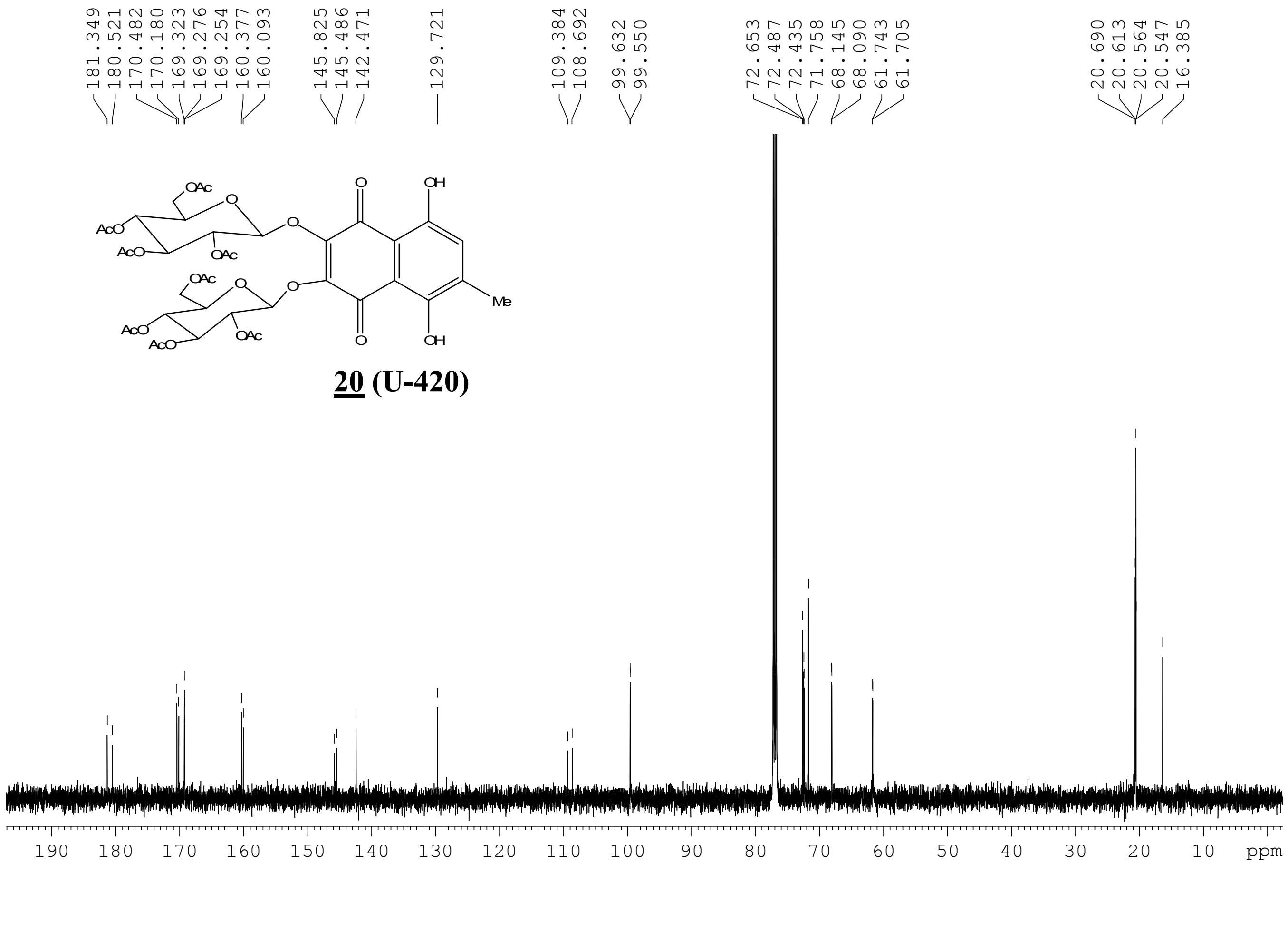

2,3-Bis(tetra-O-acetyl-β-D-glucopyranosyl-1-oxy)-6-ethyl-5,8-dihydroxynaphthalene-1,4-dione **21** *(U-421)*, *(^{1}H NMR - 500 MHz, ^{13}C NMR - 125 MHz, solvent - $CDCl_3$)*

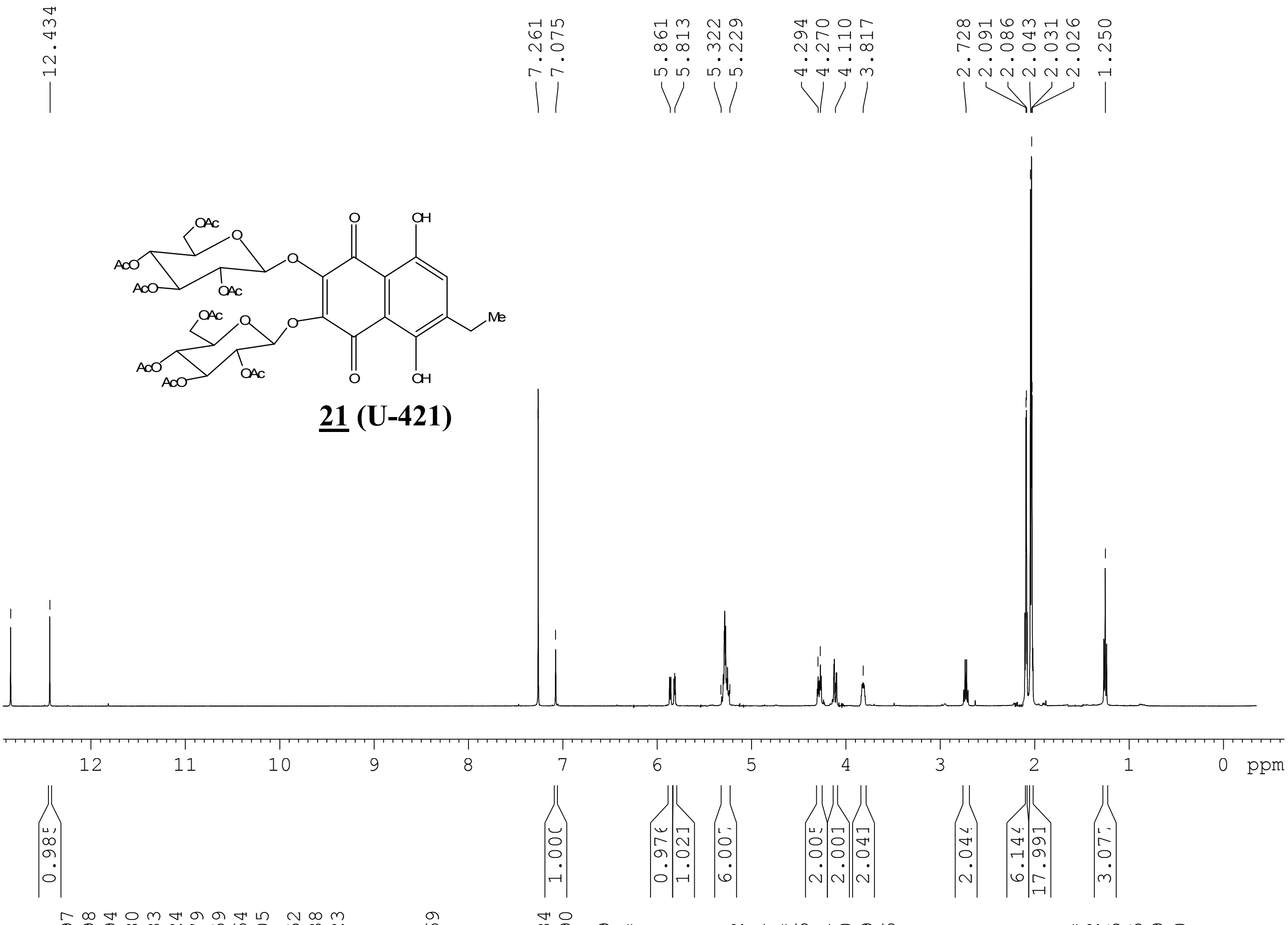

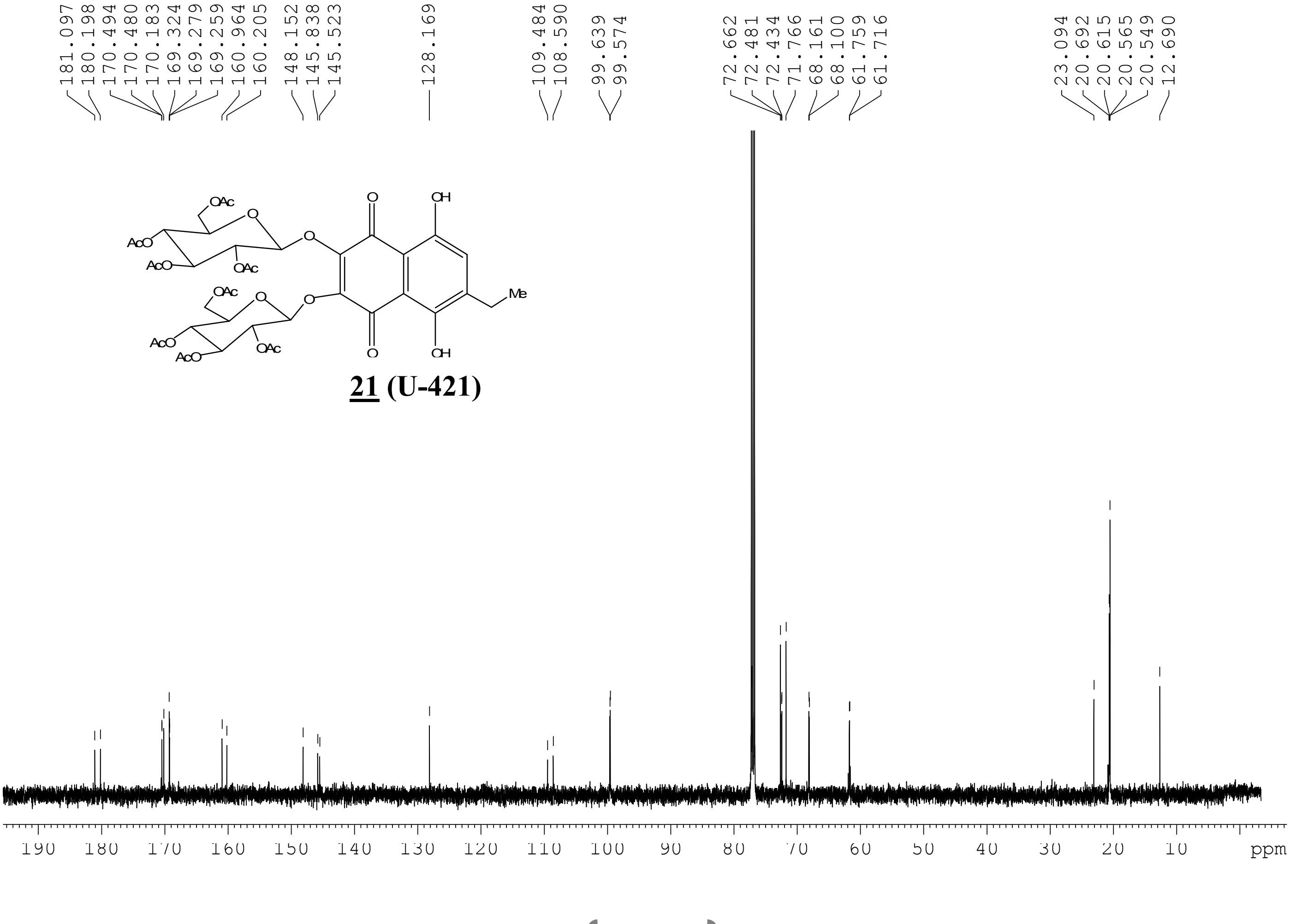

2-(Tetra-O-acetyl-β-D-glucopyranosyl-1-oxy)-5,8-dihydroxy-6,7-dimethylnaphthalene-1,4-dione **23** *(U-330)*, *(^{1}H NMR - 500 MHz, ^{13}C NMR - 125 MHz, solvent - $CDCl_3$)*

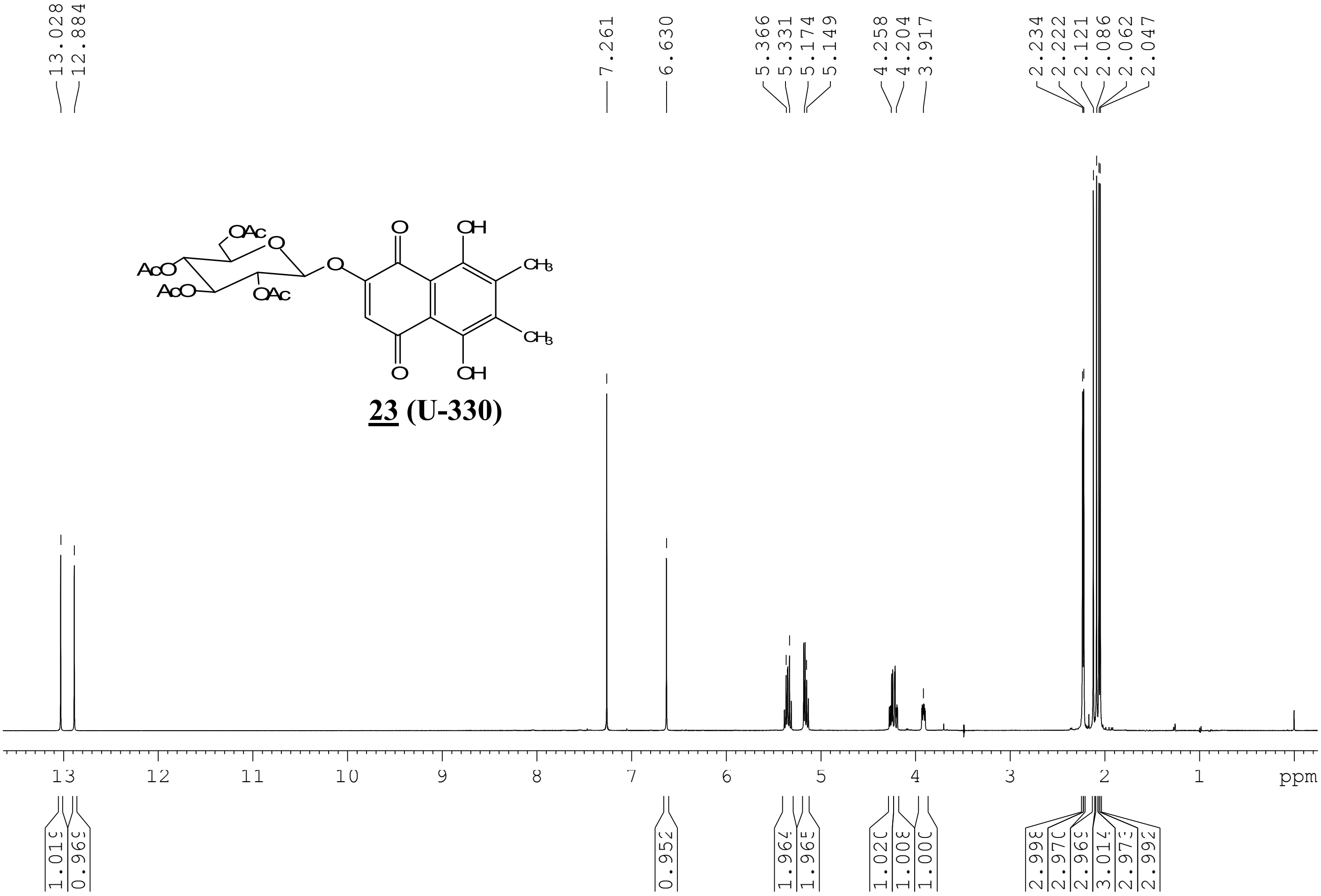

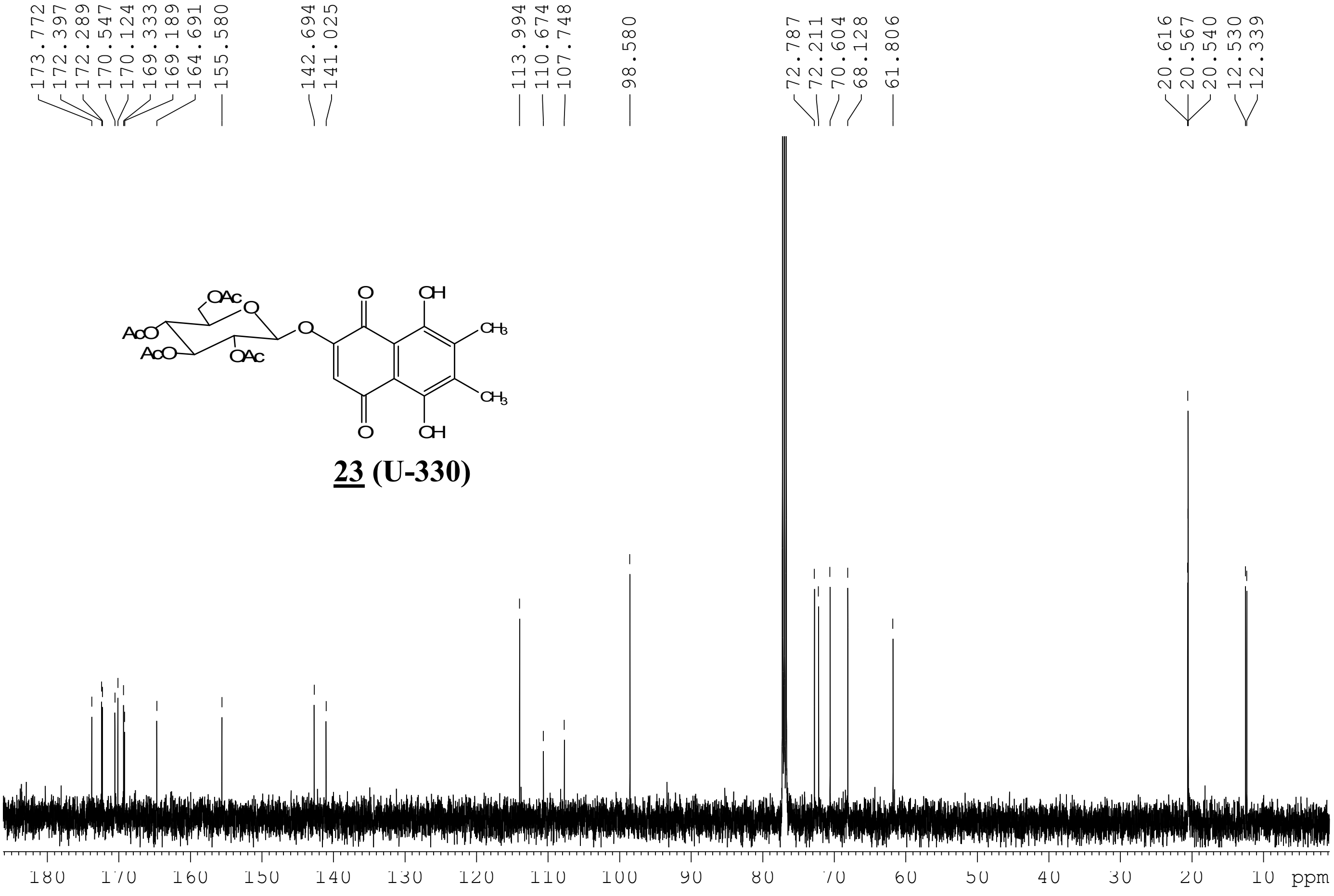

3-(Tetra-O-acetyl-β-D-glucopyranosyl-1-thiomethyl)-2,5,8-trihydroxynaphthalene-1,4-dione ***28*** *(U-633)***,** *(^{1}H NMR - 500 MHz, ^{13}C NMR - 125 MHz, solvent - $CDCl_3$)*

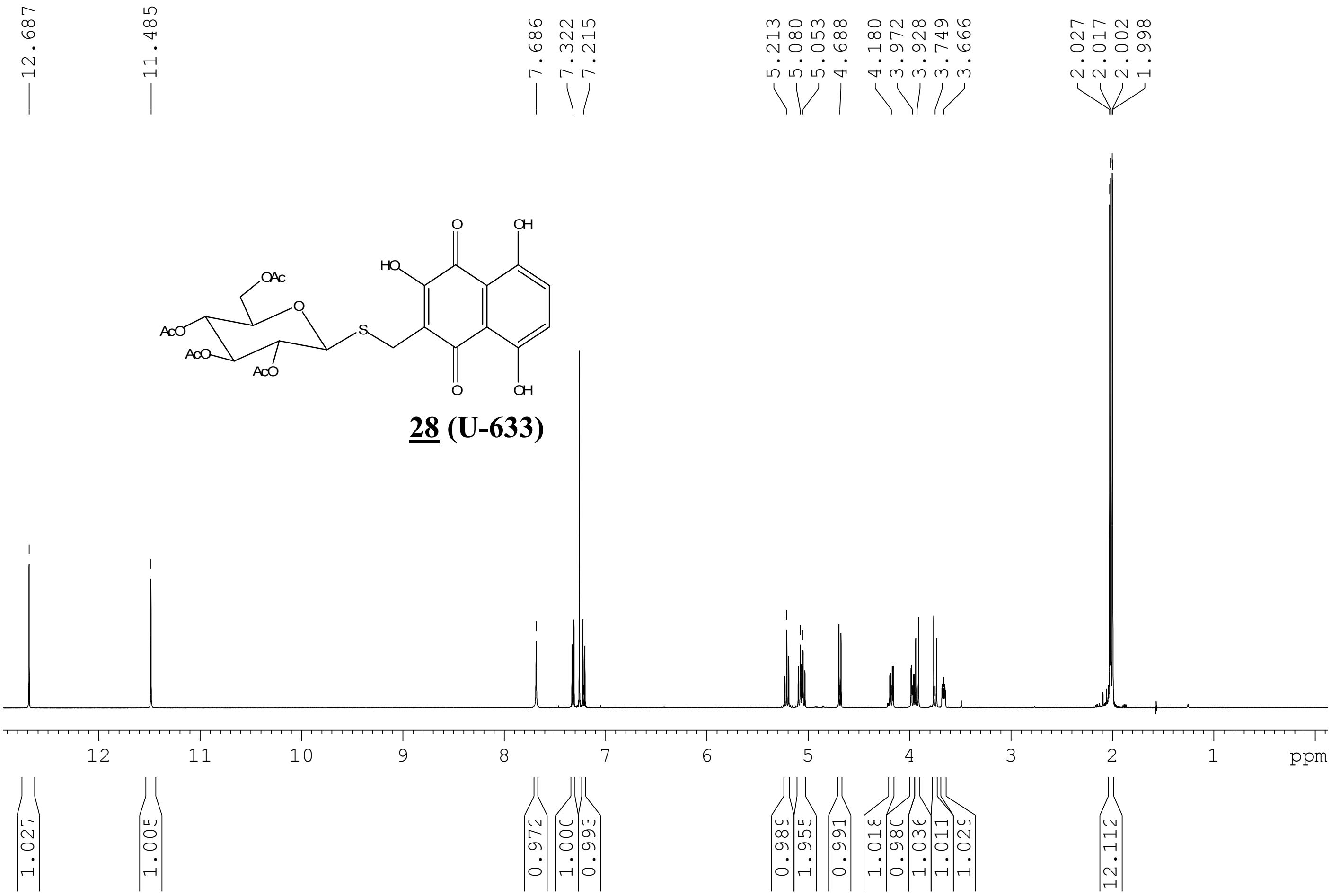

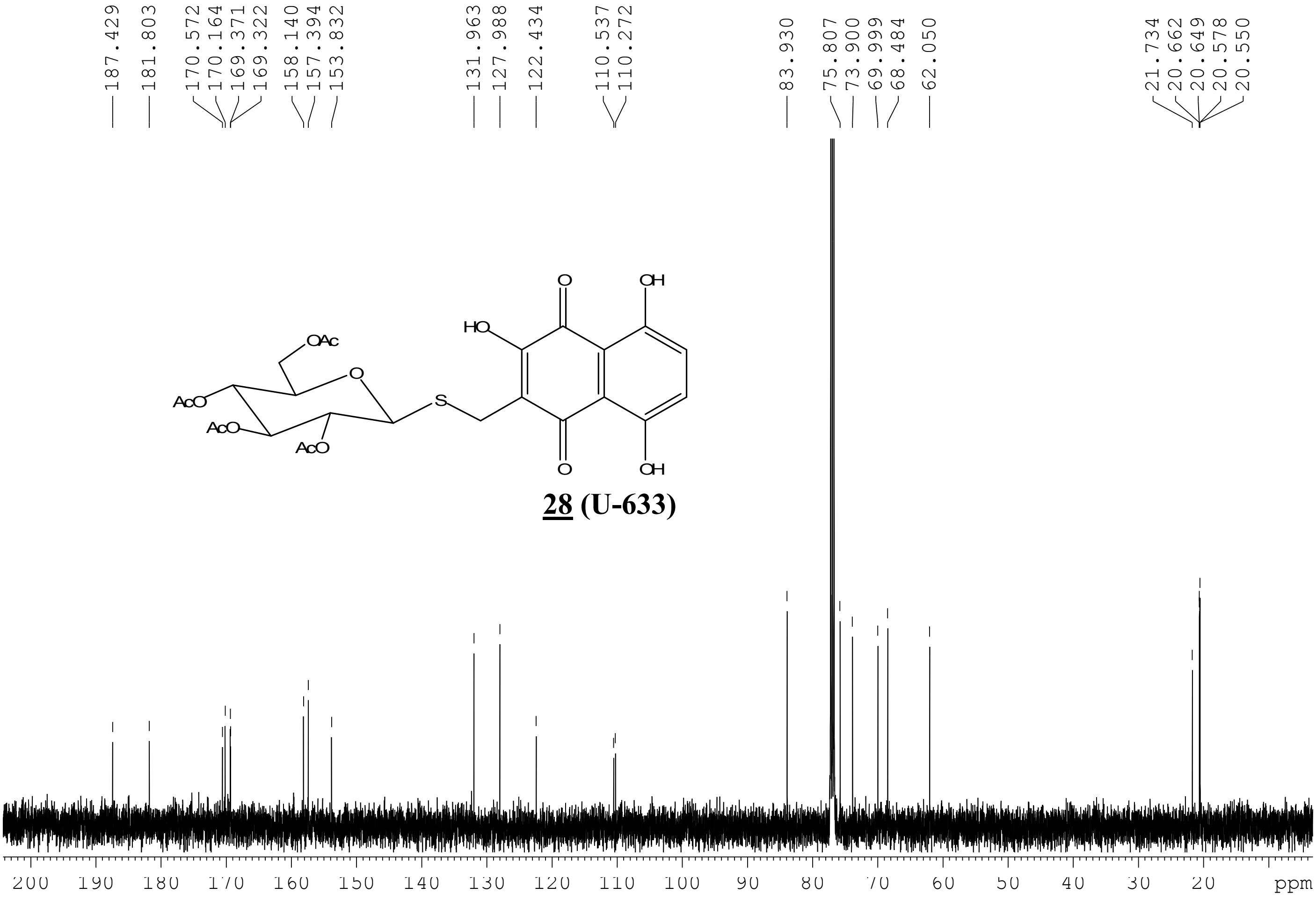

3-(Tetra-O-acetyl-β-D-glucopyranosyl-1-thiomethyl)-2,5,8-trihydroxy-6,7-dimethylnaphthalene-1,4-dione ***29*** *(U-519), (^{1}H NMR - 500 MHz, ^{13}C NMR - 125 MHz, solvent - CDCl₃)*

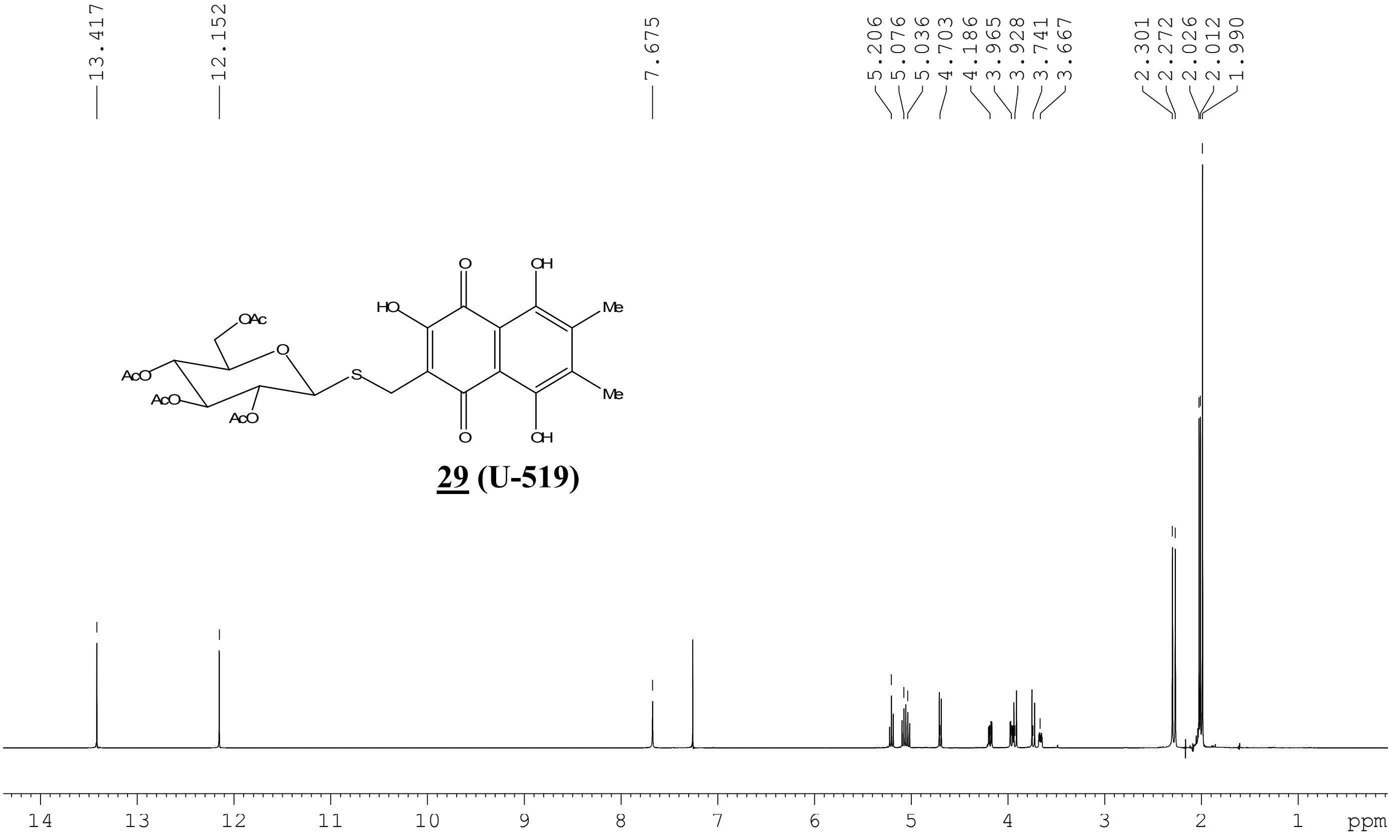

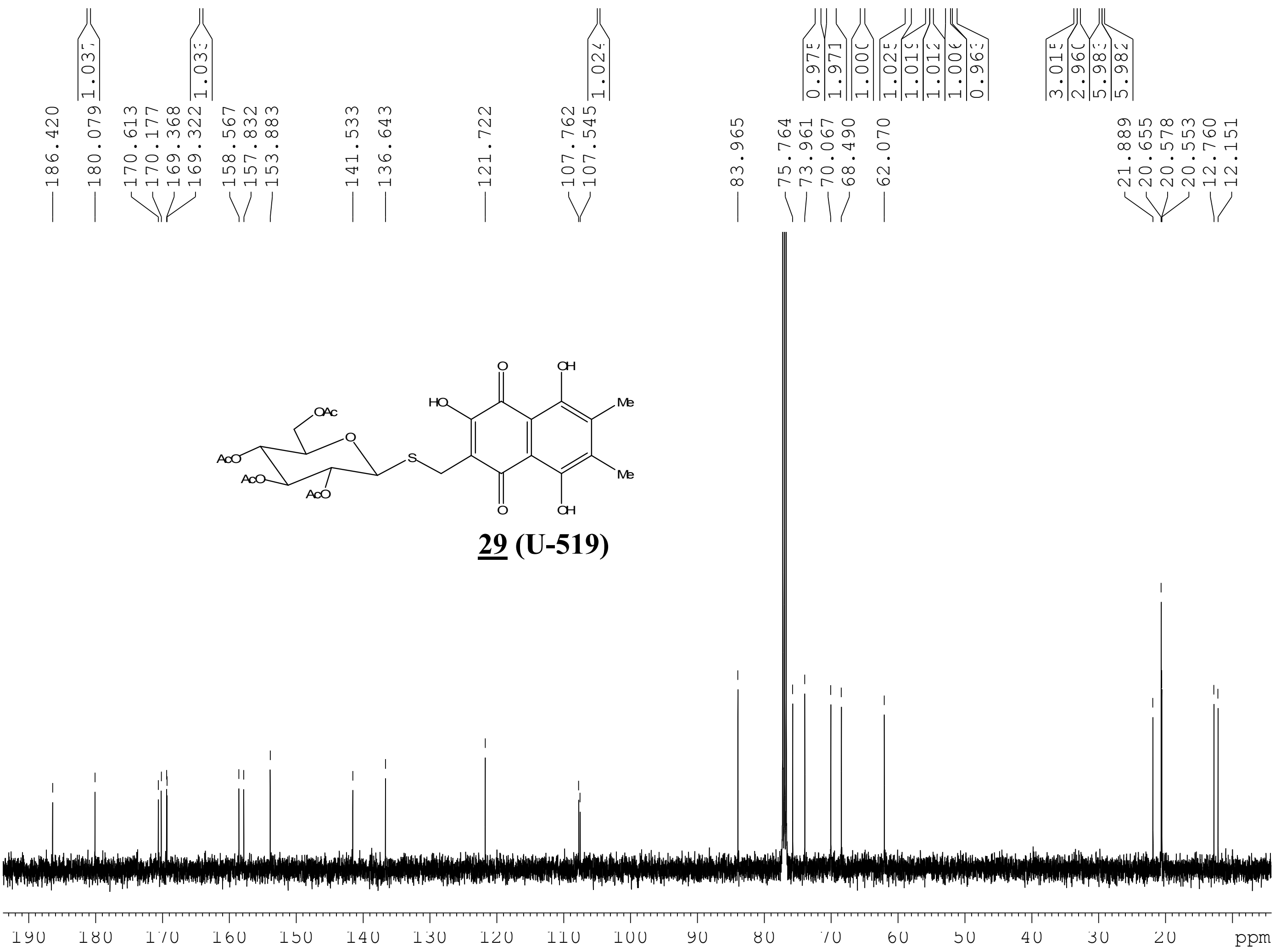

3-(Tetra-O-acetyl-β-D-glucopyranosyl-1-thiomethyl)-6,7-dichloro-2,5,8-trihydroxynaphthalene-1,4-dione ***30*** *(U-518)****,*** *(^{1}H NMR - 300 MHz, ^{13}C NMR - 75 MHz, solvent - $CDCl_3$)*

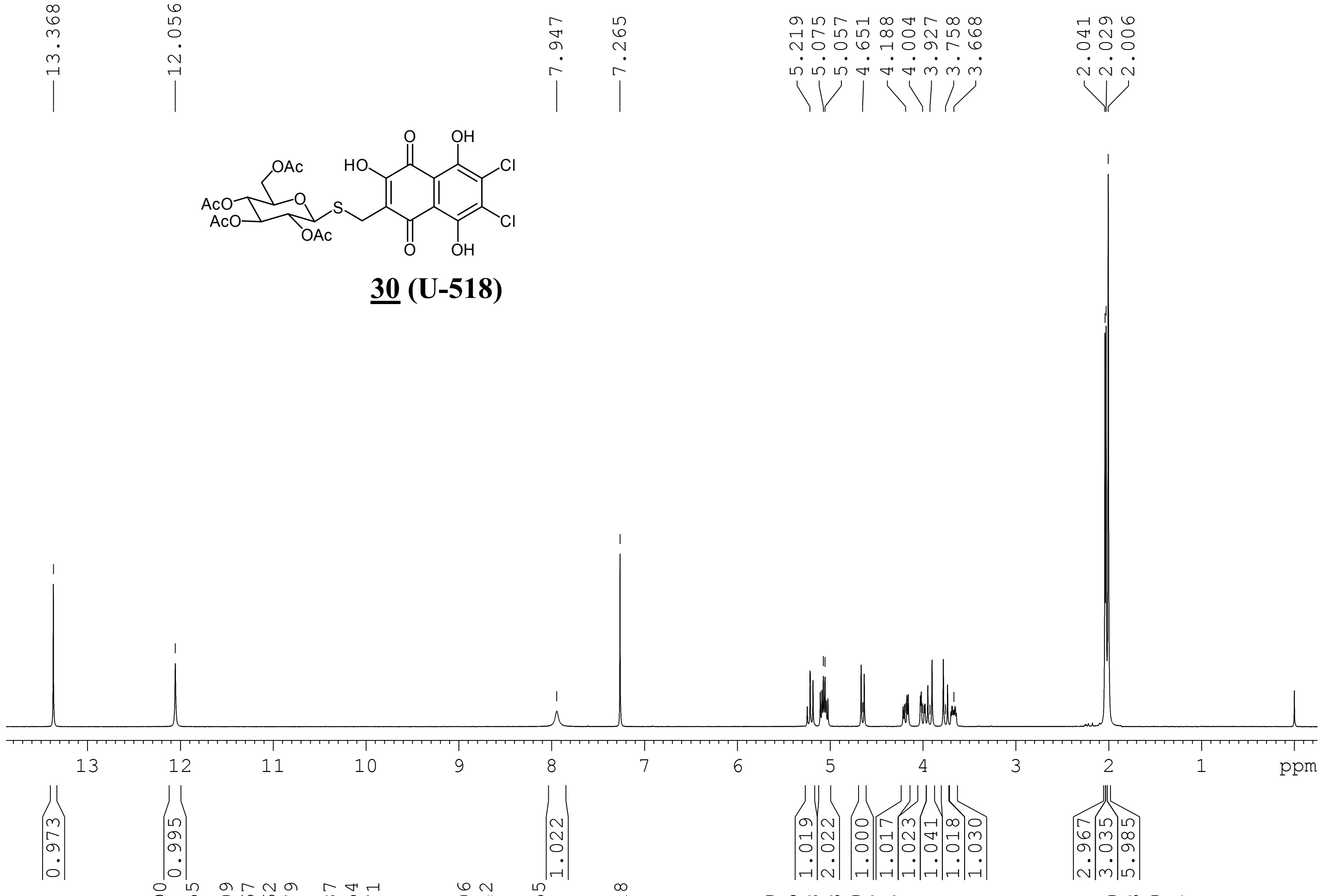

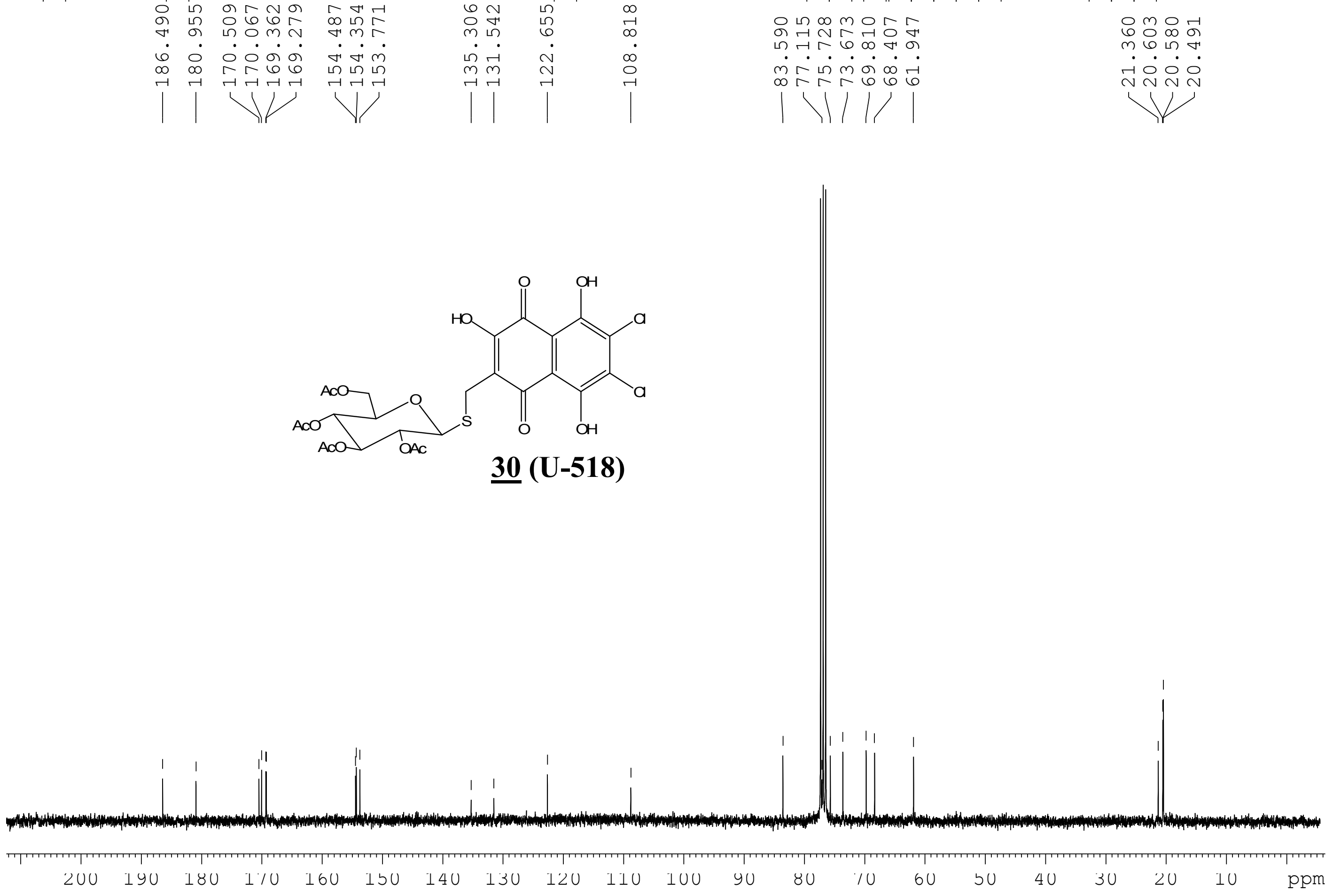

*3-(Tetra-O-acetyl-β-D-glucopyranosyl-1-thiomethyl)-2,5,8-trihydroxy-7-methoxynaphthalene-1,4- dione **31** (U-639)*, *(^{1}H NMR - 500 MHz, ^{13}C NMR - 125 MHz, solvent - $CDCl_3$)*

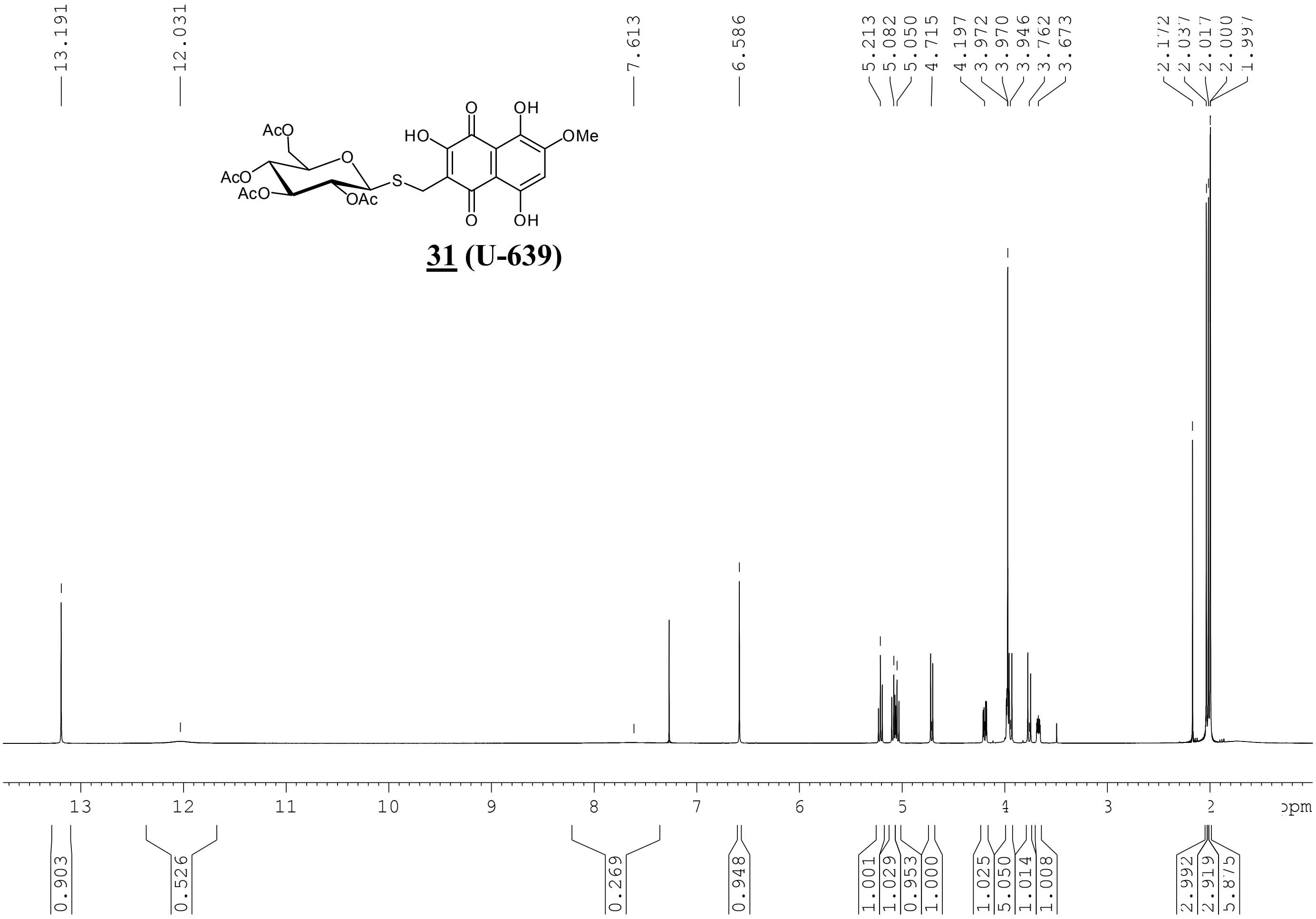

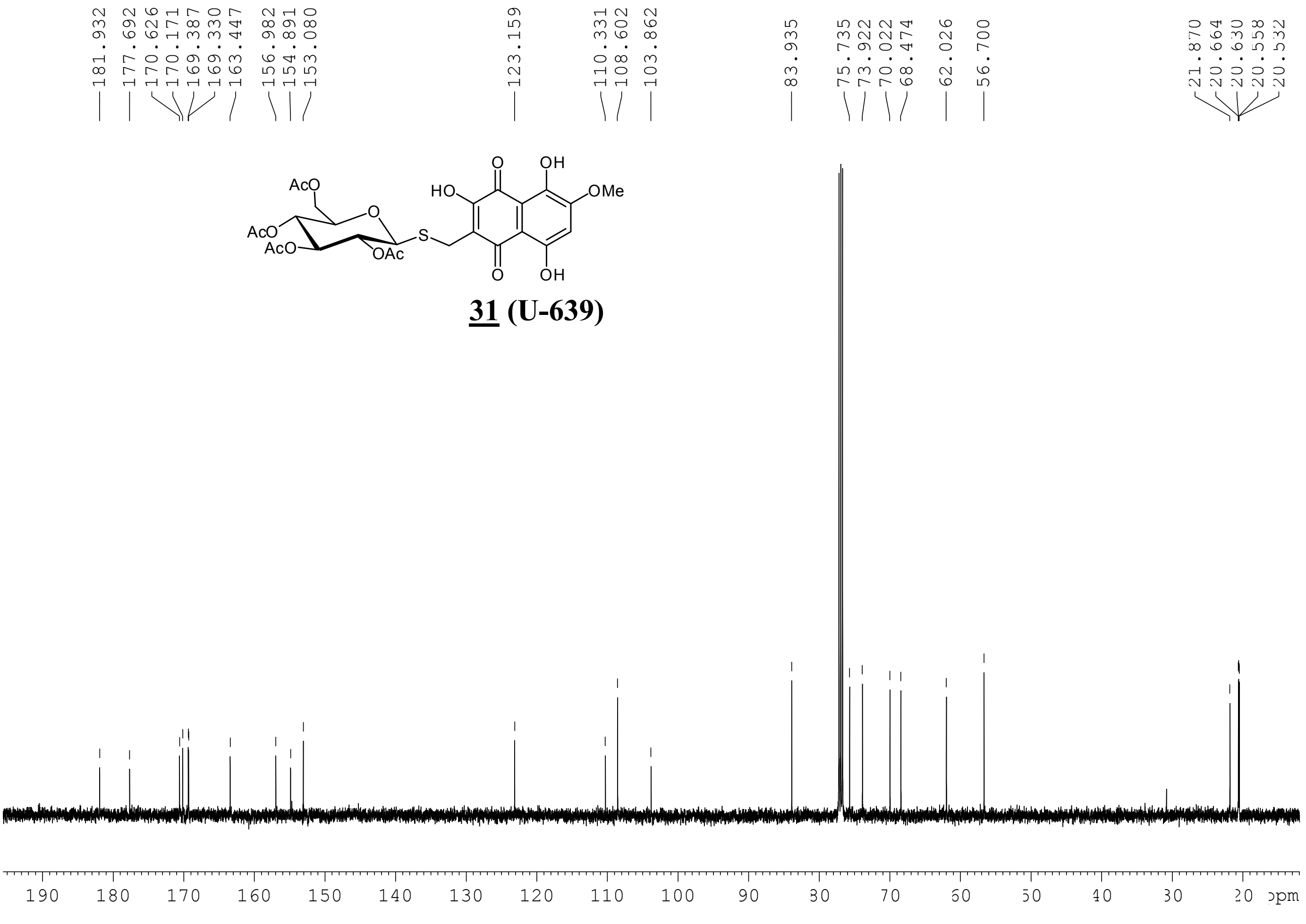

3-(Tetra-O-acetyl-β-D-glucopyranosyl-1-thiomethyl)-2,5,8-trihydroxy-6,7-dimethoxynaphthalene-1,4-dione ***32*** *(U-637)*, *(^{1}H NMR - 500 MHz, ^{13}C NMR - 125 MHz, solvent - $CDCl_3$)*

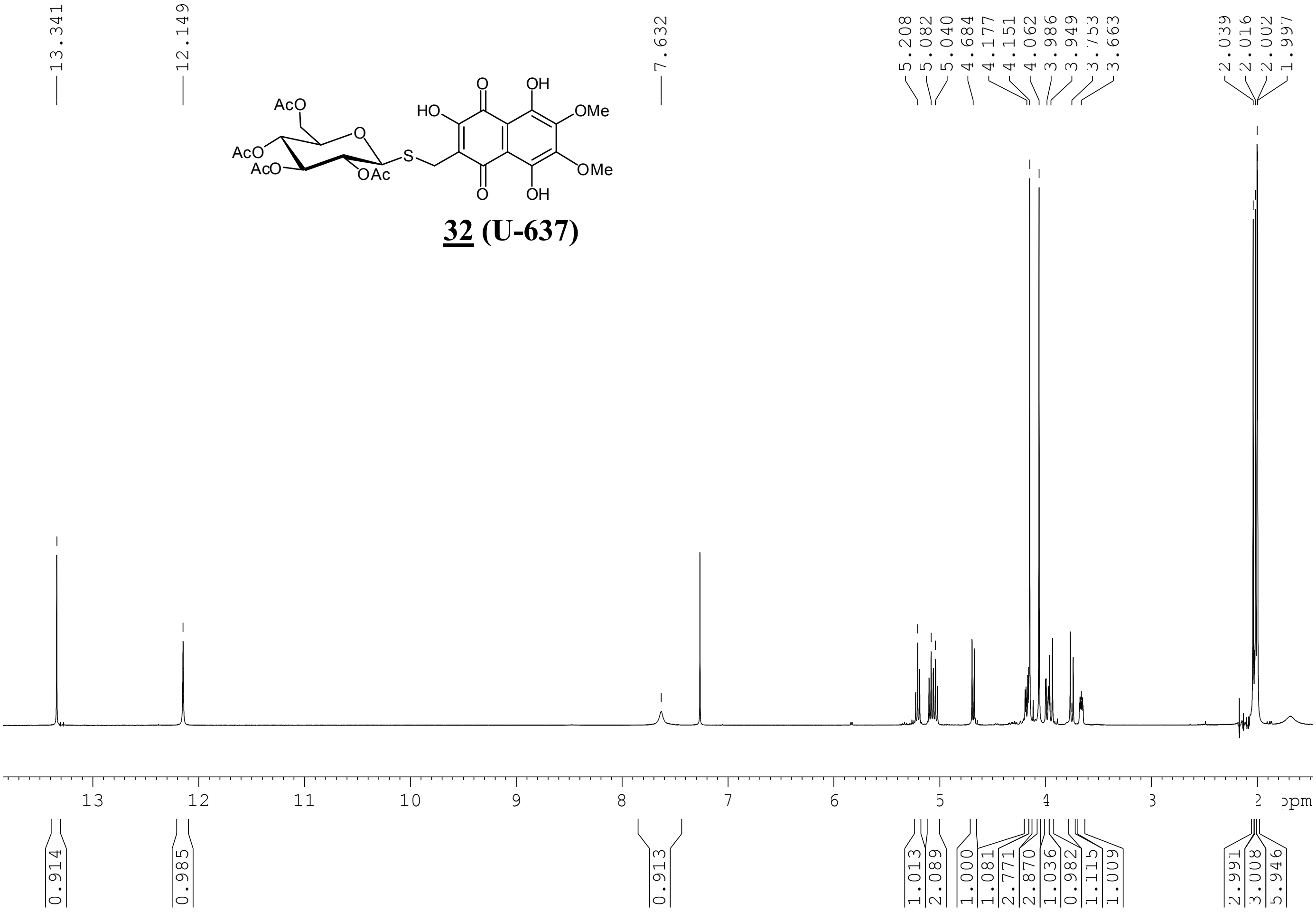

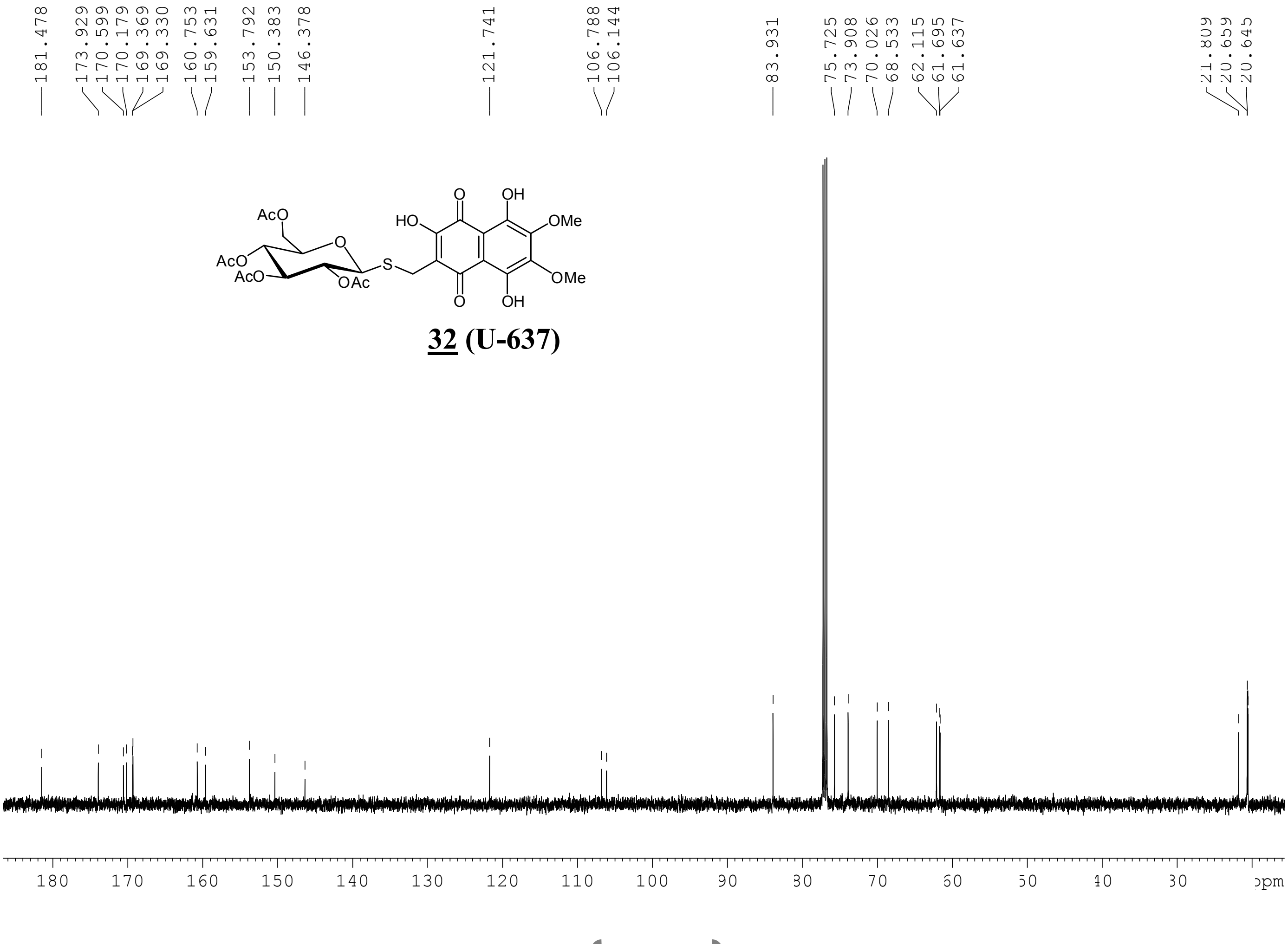

3-(β-D-Glucopyranosyl-1-thiomethyl)-2,5,8-trihydroxynaphthalene-1,4-dione ***38*** *(U-635)*, *(^{1}H NMR - 500 MHz, ^{13}C NMR - 125 MHz, solvent – DMSO-d_6)*

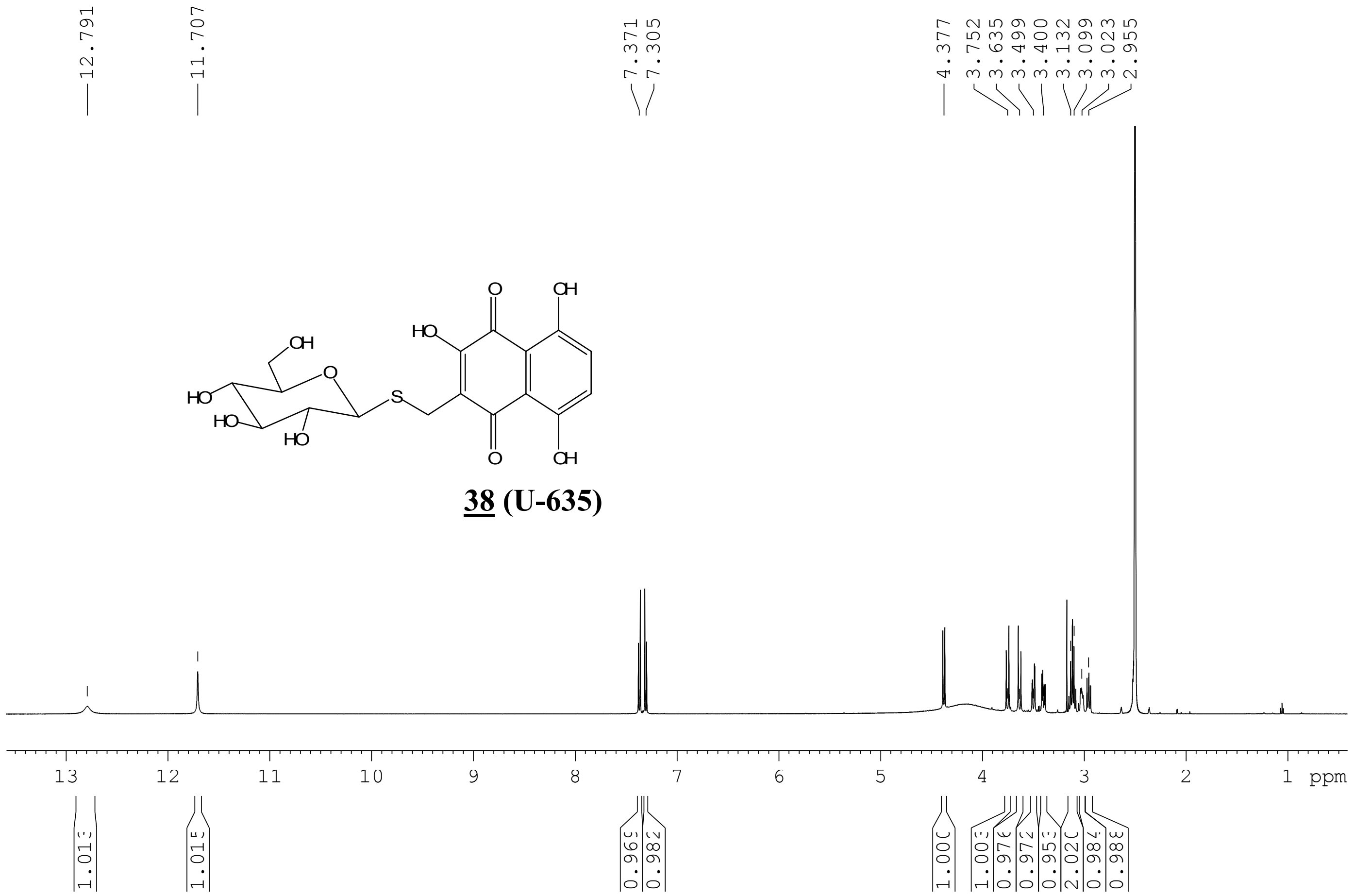

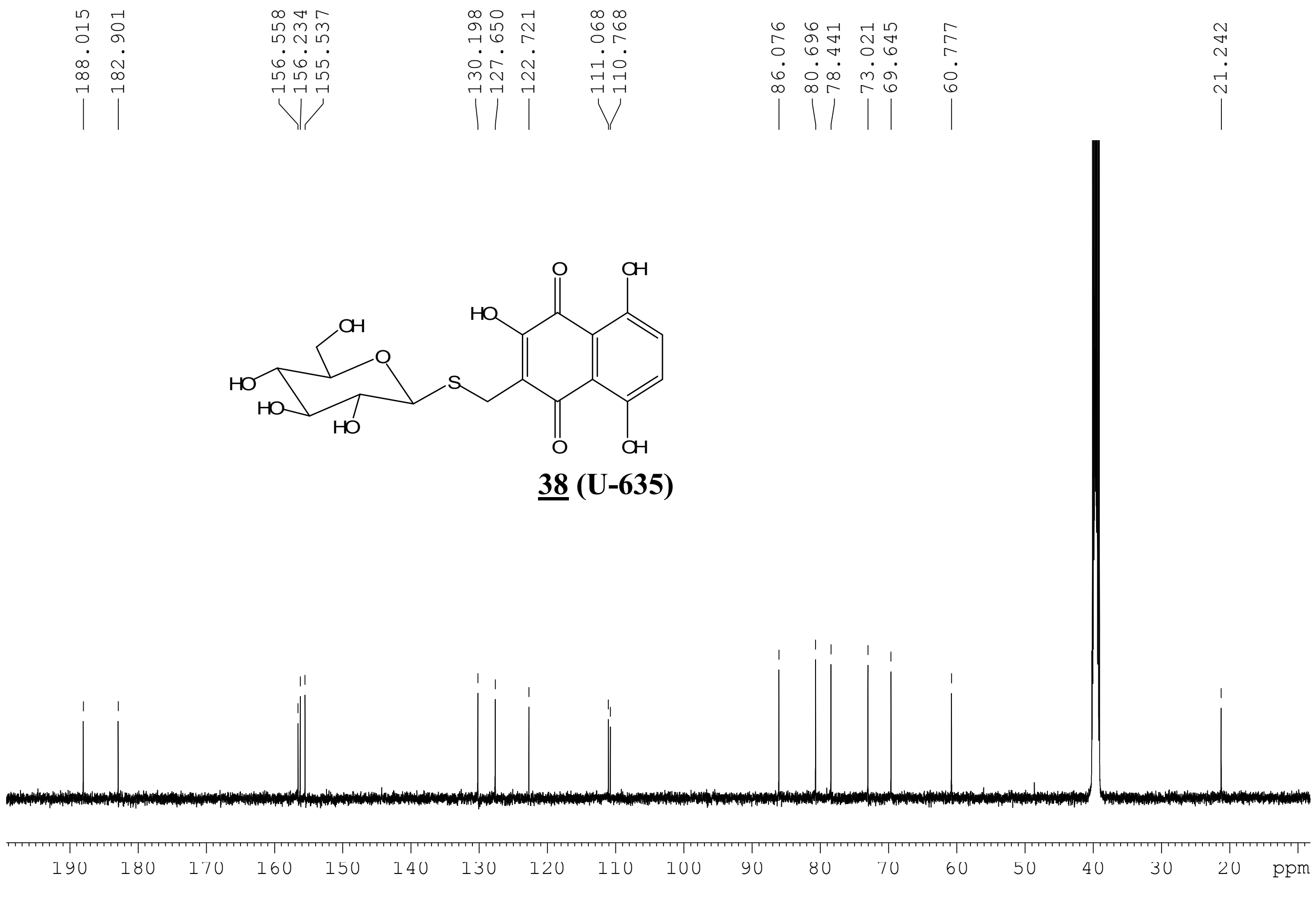

*3-(β-D-Glucopyranosyl-1-thiomethyl)-2,5,8-trihydroxy-6,7-dimethylnaphthalene-1,4-dione **39** (U-520)*, *(^{1}H NMR - 500 MHz, ^{13}C NMR - 125 MHz, solvent – DMSO-d₆)*

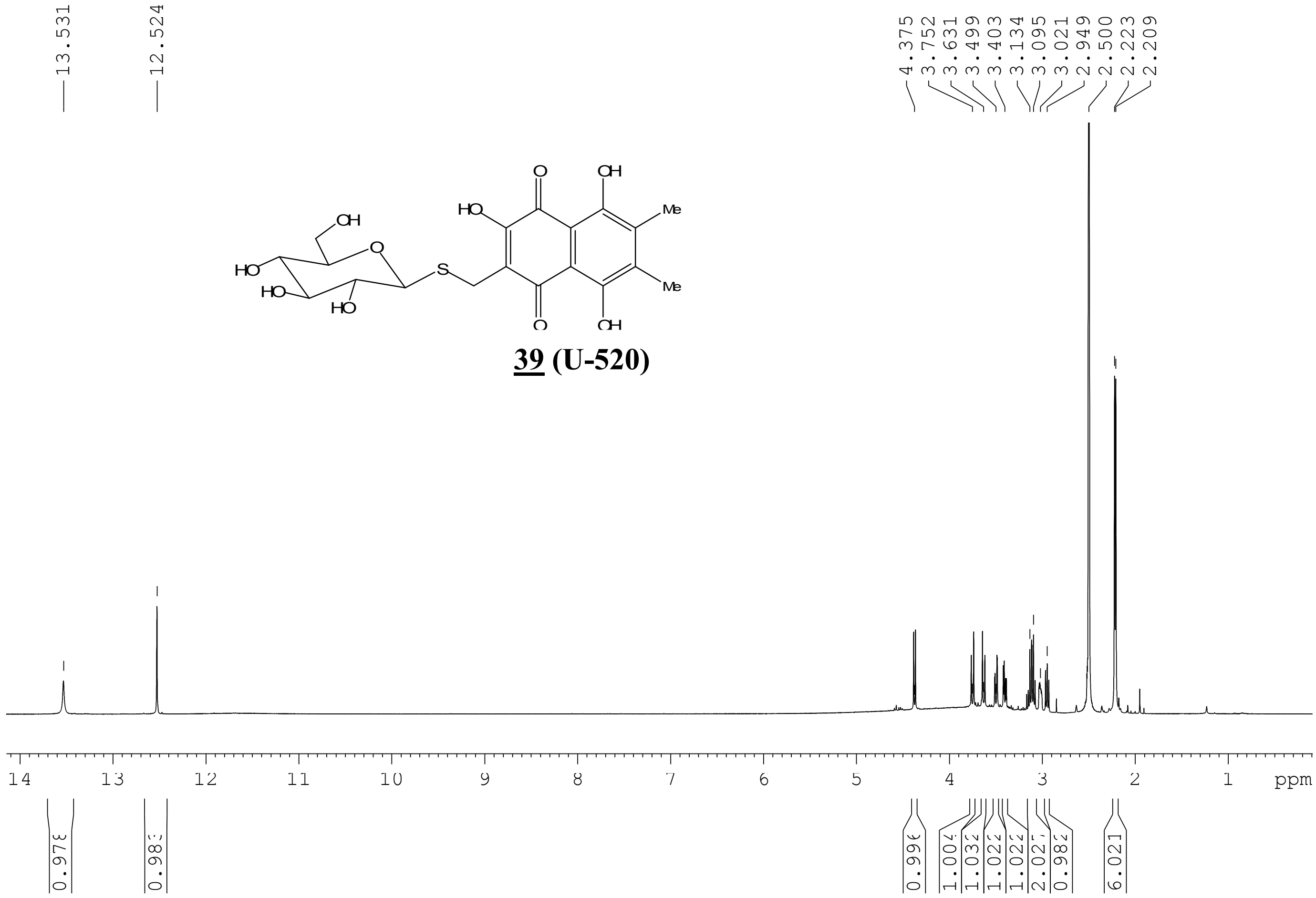

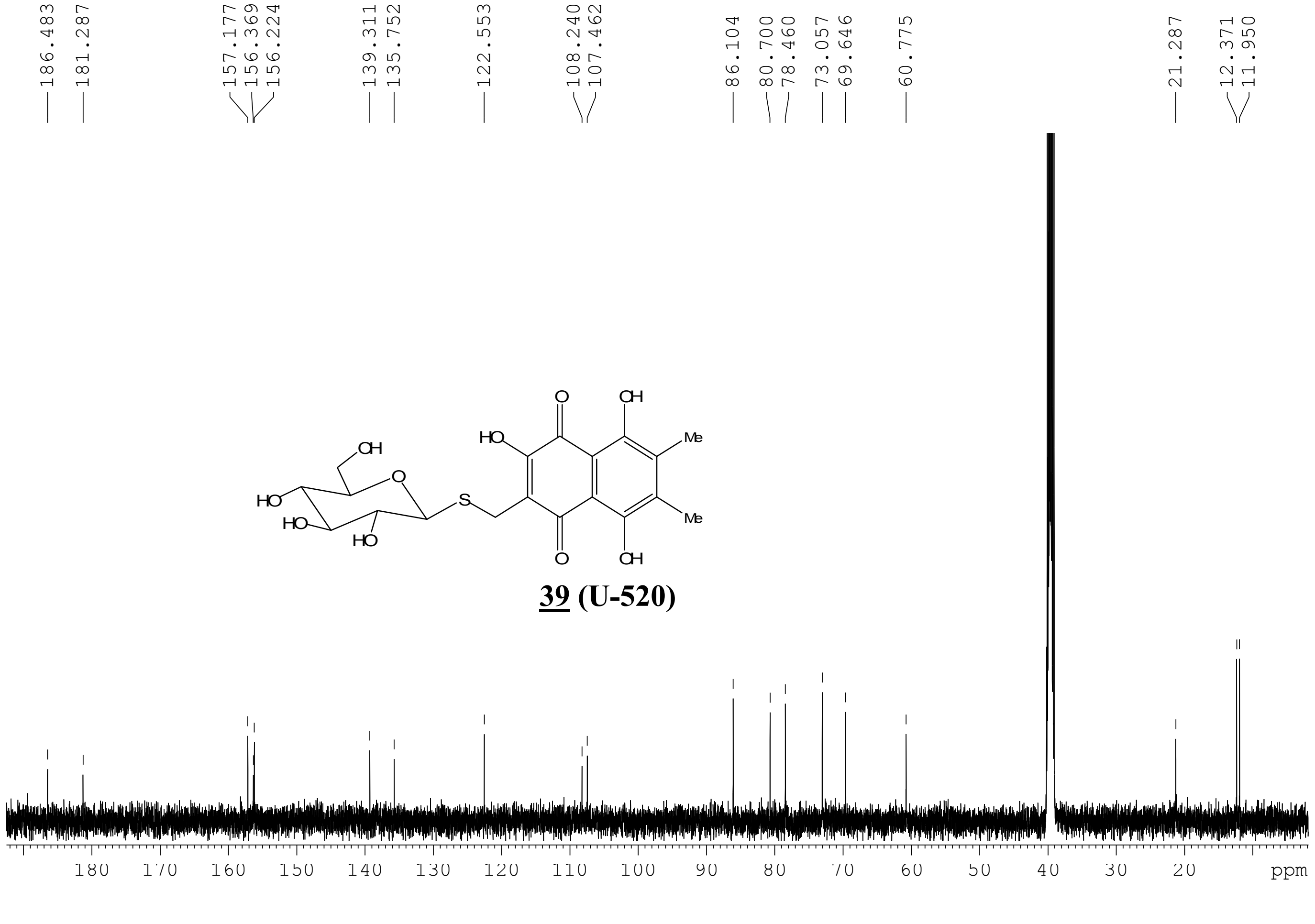

6,7-Dichloro-3-(β-D-glucopyranosyl-1-thiomethyl)-2,5,8-trihydroxynaphthalene-1,4-dione ***40*** *(U-624)*, *(^{1}H NMR - 500 MHz, ^{13}C NMR - 125 MHz, solvent – DMSO-d$_6$)*

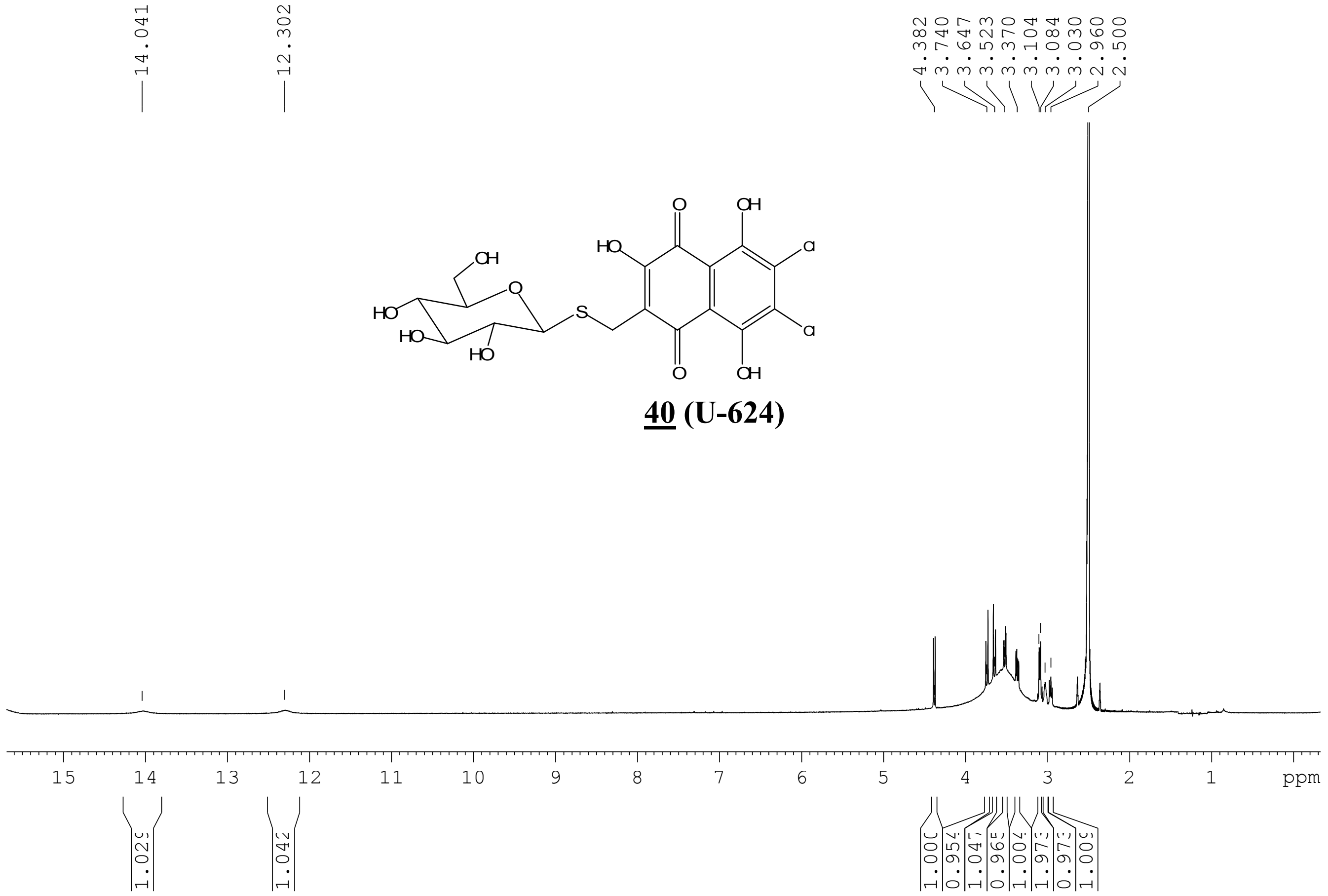

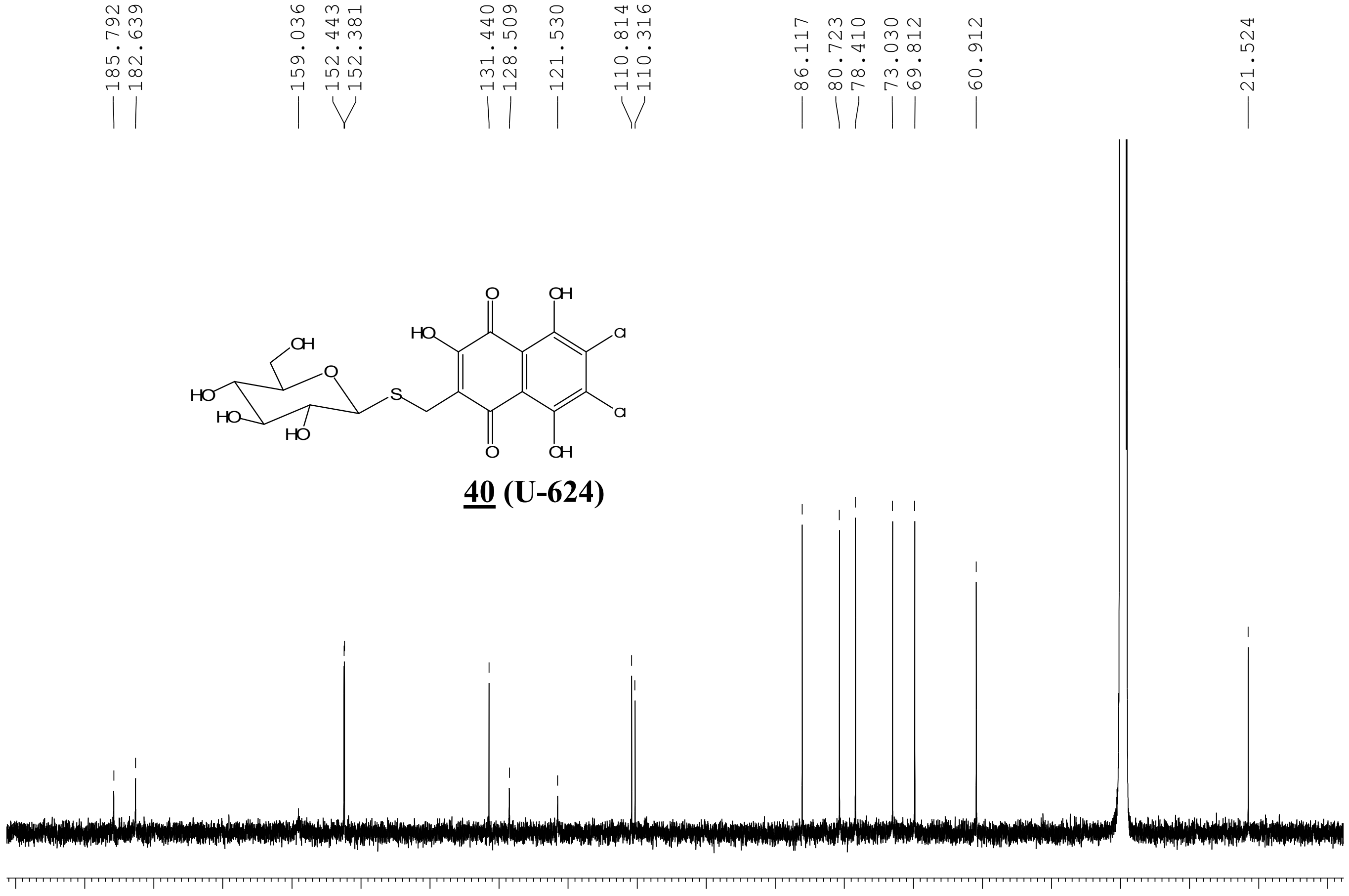

3-(β-D-Glucopyranosyl-1-thiomethyl)-2,5,8-trihydroxy-7-methoxynaphthalene-1,4-dione **41** *(U-644), (^{1}H NMR - 700 MHz, ^{13}C NMR - 176 MHz, solvent – DMSO-d_6)*

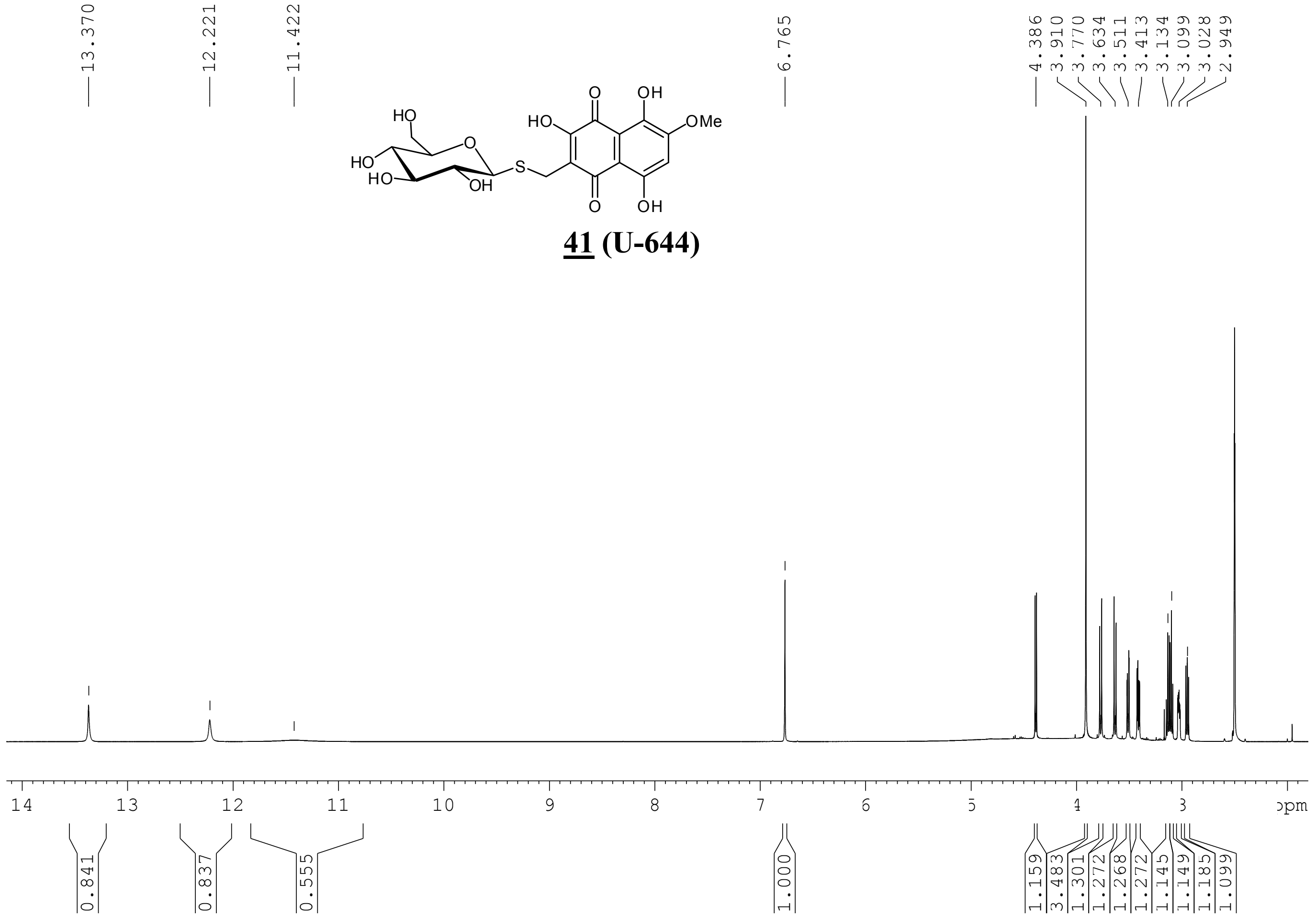

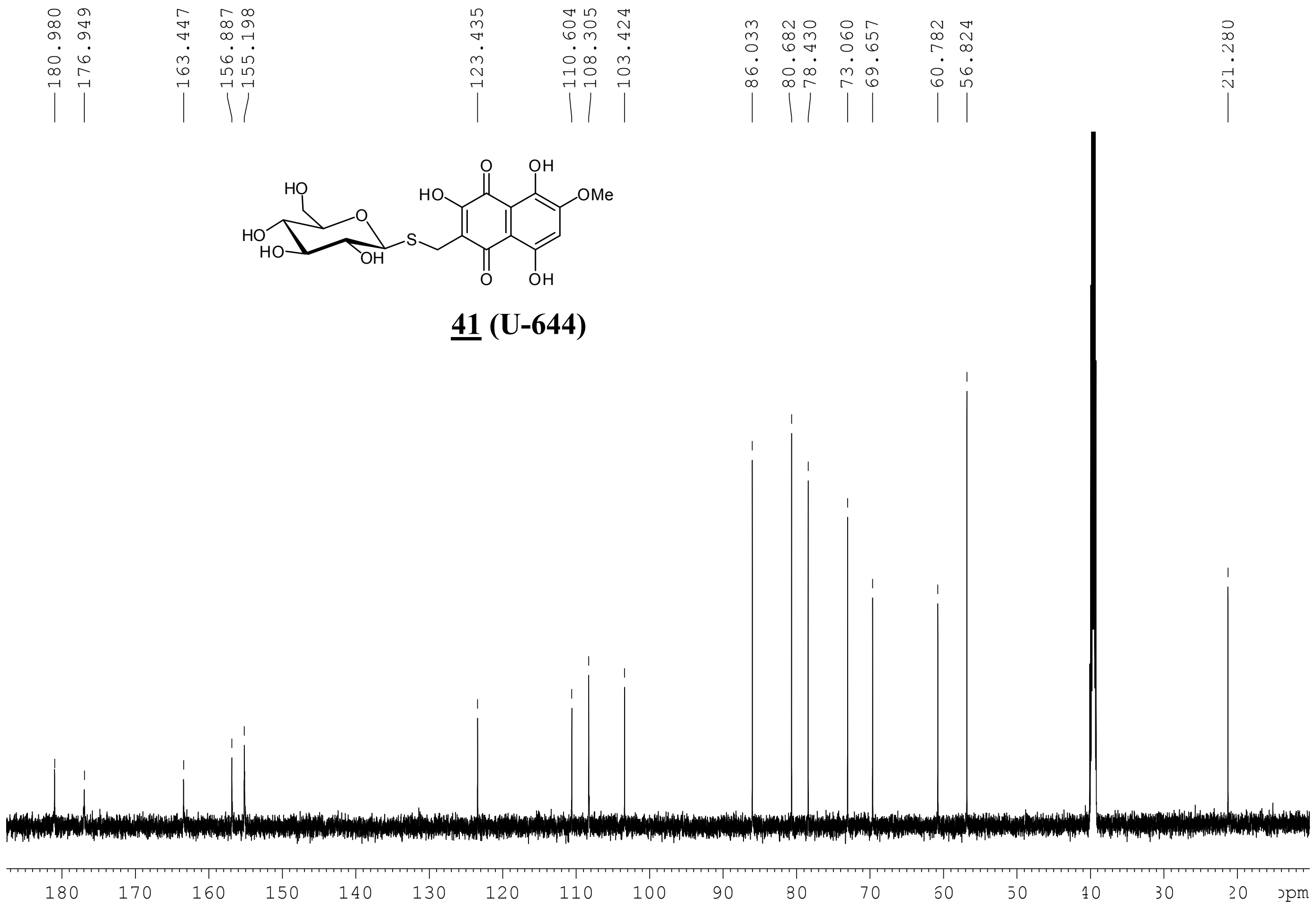

3-(β-D-Glucopyranosyl-1-thiomethyl)-2,5,8-trihydroxy-6,7-dimethoxynaphthalene-1,4-dione **42** *(U-640)*, *(^{1}H NMR - 500 MHz, ^{13}C NMR - 125 MHz, solvent – DMSO-d_6)*

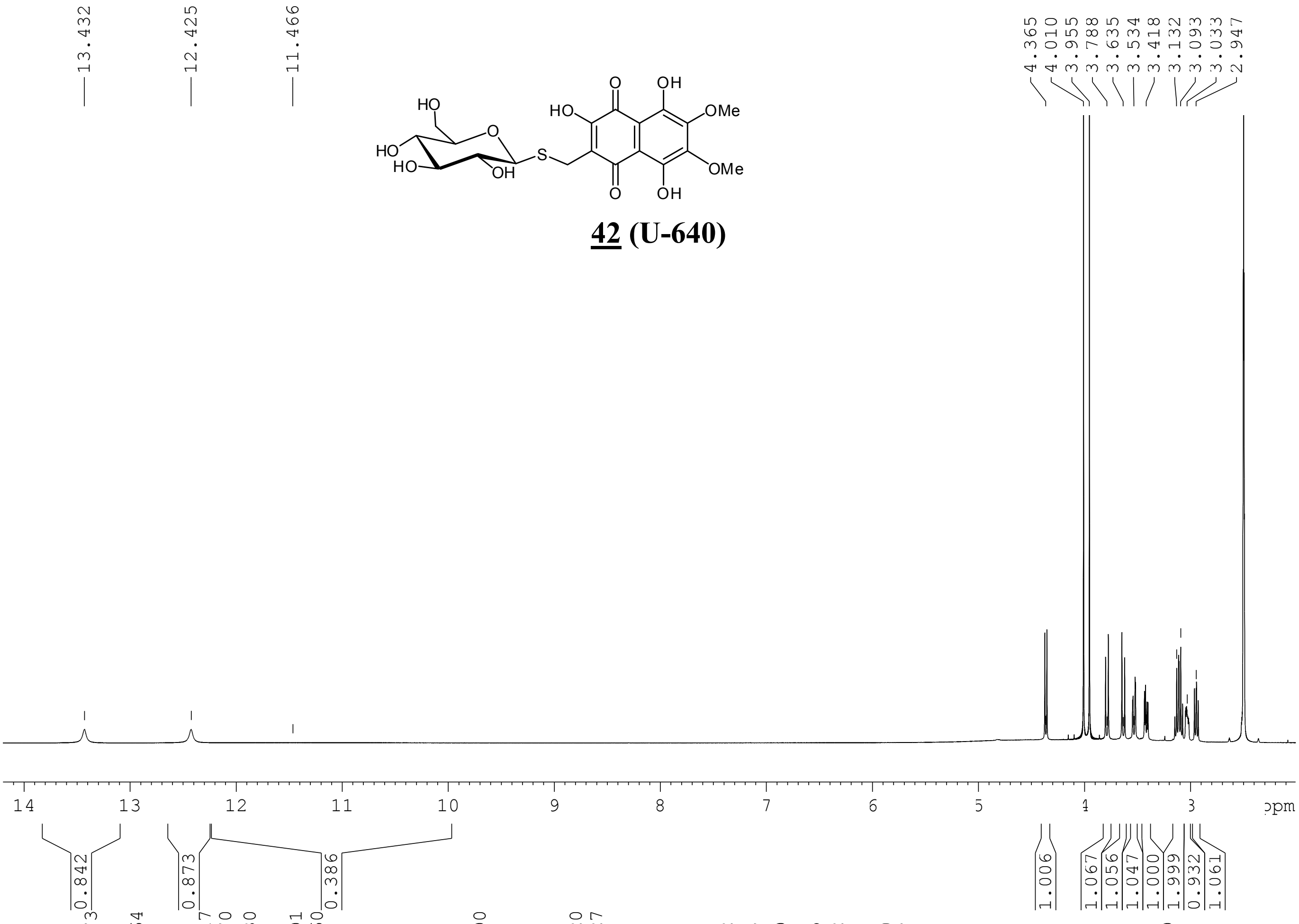

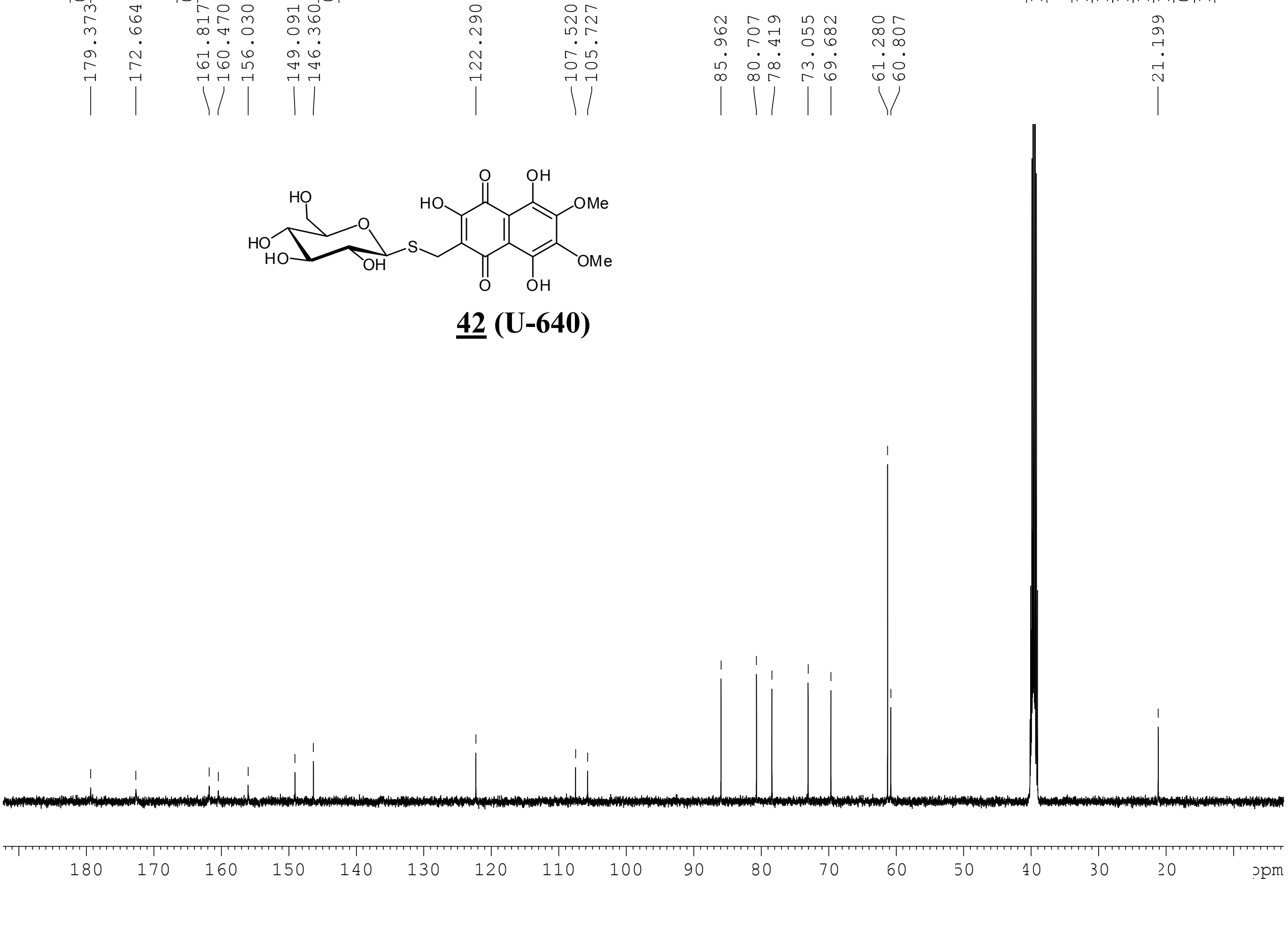

3-(Tetra-O-acetyl-β-D-glucopyranosyl-1-thiomethyl)-5,8-dihydroxy-2-methoxynaphthalene-1,4-dione ***43*** *(U-634)*, *(^{1}H NMR - 500 MHz, ^{13}C NMR - 125 MHz, solvent – $CDCl_3$)*

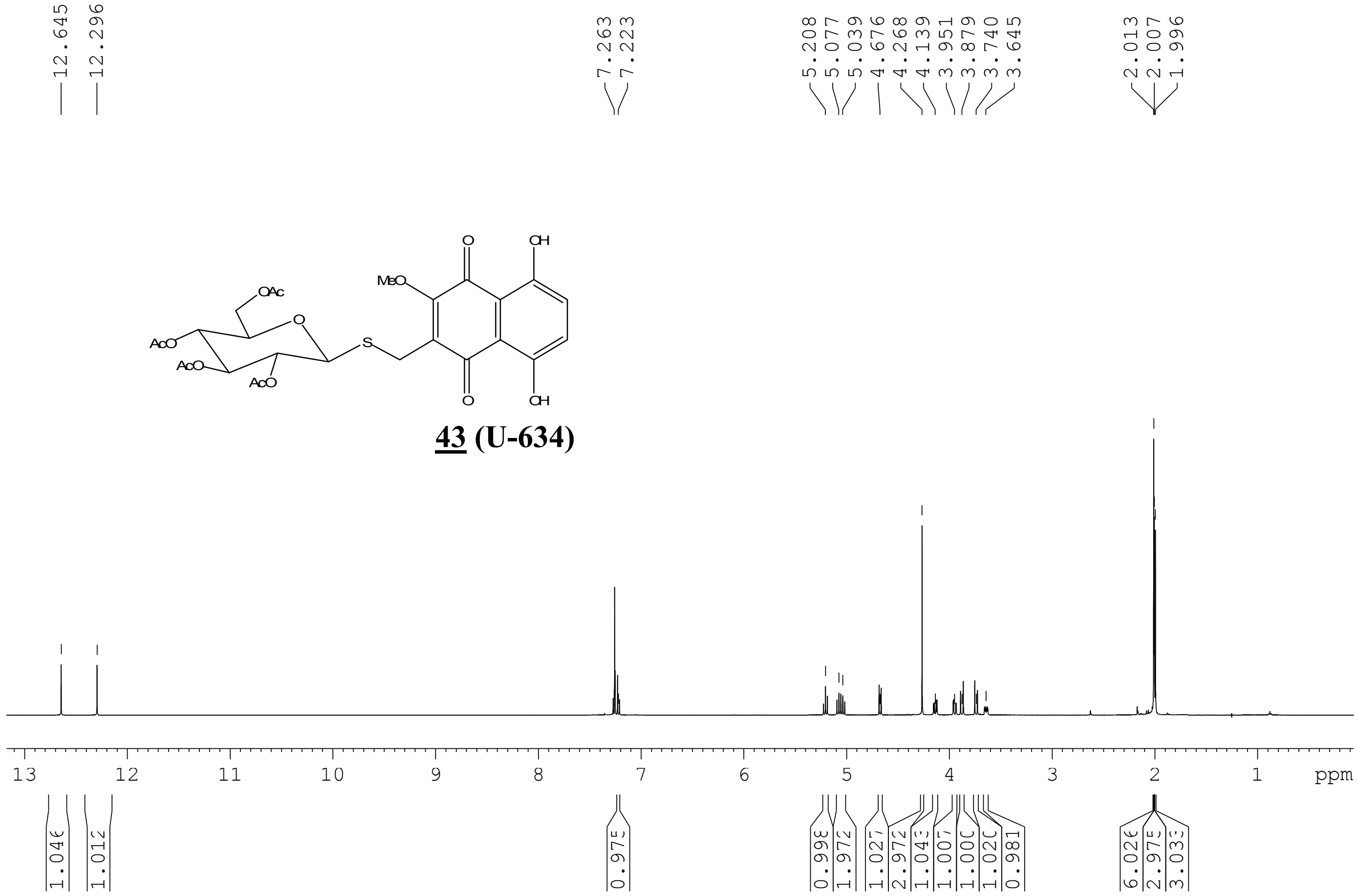

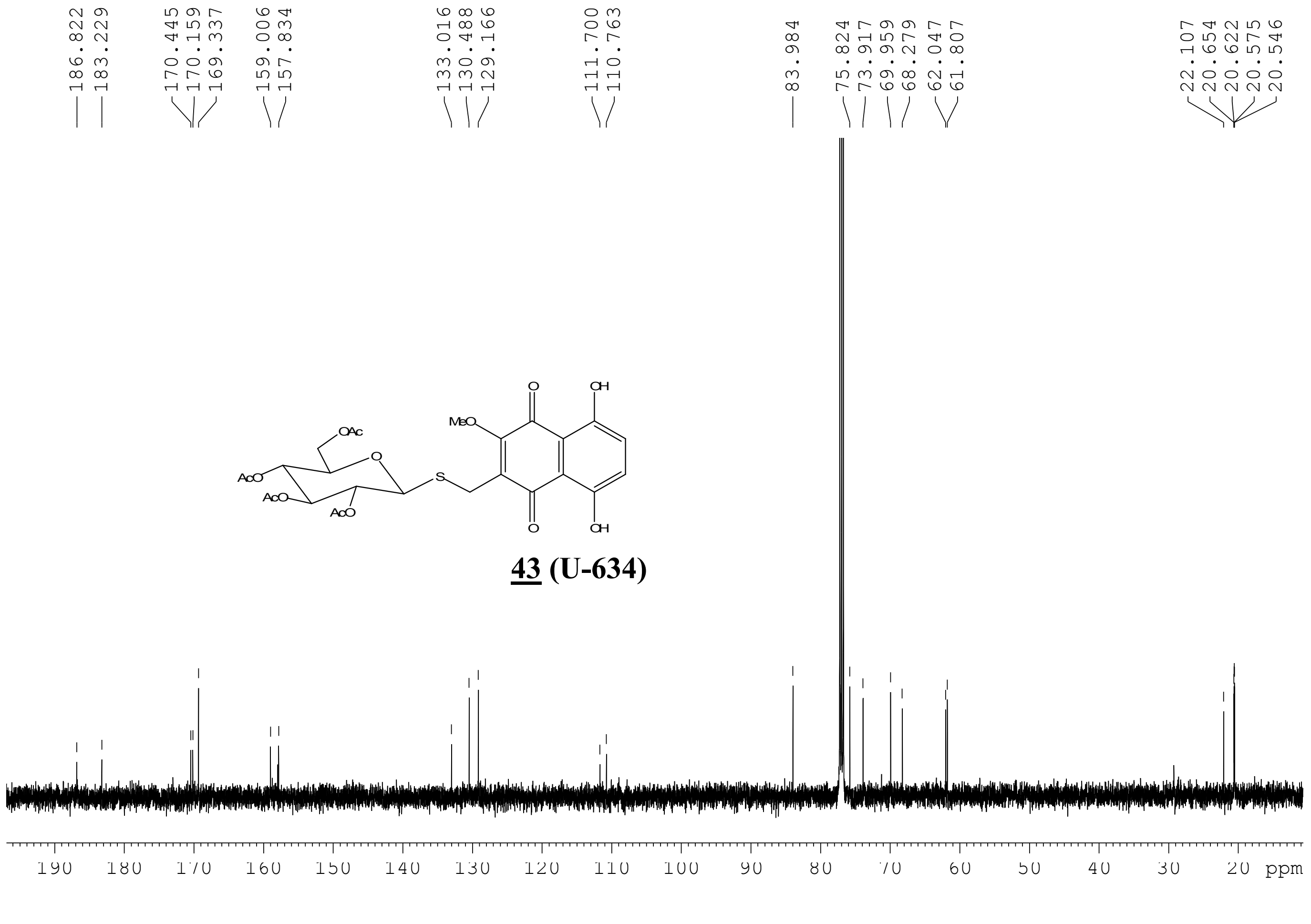

3-(Tetra-O-acetyl-β-D-glucopyranosyl-1-thiomethyl)-5,8-dihydroxy-2-methoxy-6,7-dimethylnaphthalene-1,4-dione ***44*** *(U-521),*
(^{1}H NMR - 500 MHz, ^{13}C NMR - 125 MHz, solvent – $CDCl_3$)

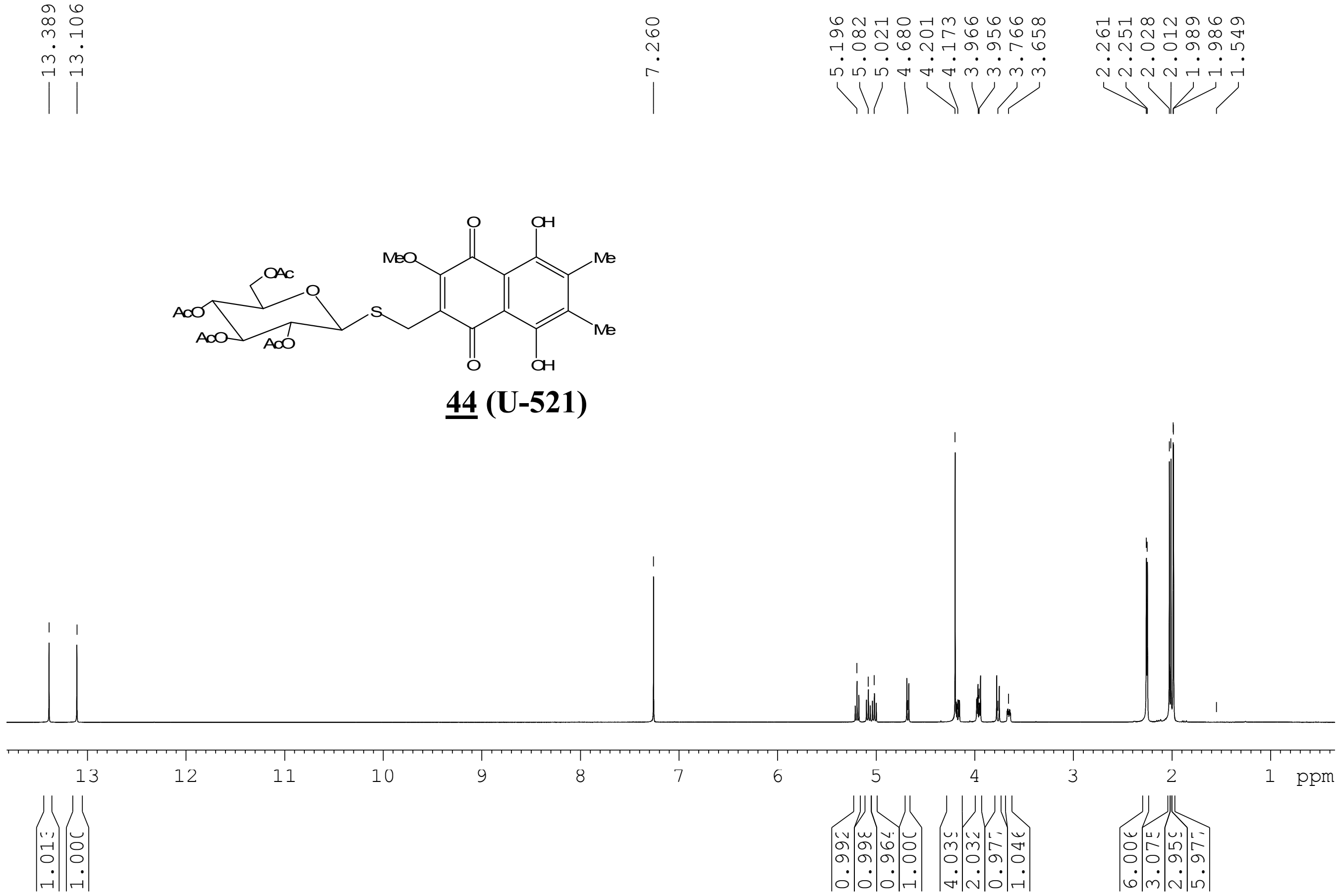

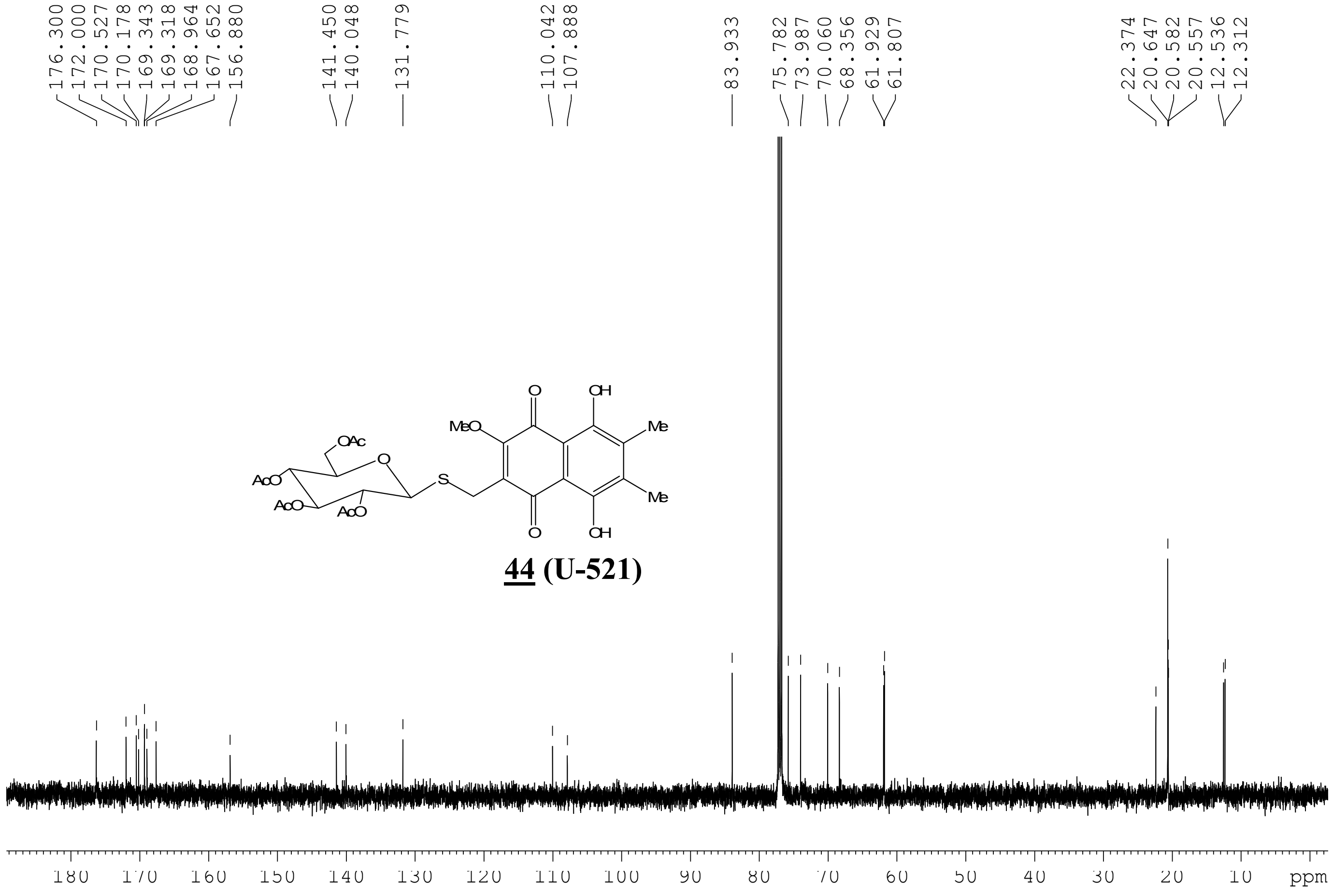

3-(Tetra-O-acetyl-β-D-glucopyranosyl-1-thiomethyl)-6,7-dichloro-5,8-dihydroxy-2-methoxynaphthalene-1,4-dione **45** *(U-523)*, *(^{1}H NMR - 500 MHz, ^{13}C NMR - 125 MHz, solvent – $CDCl_3$)*

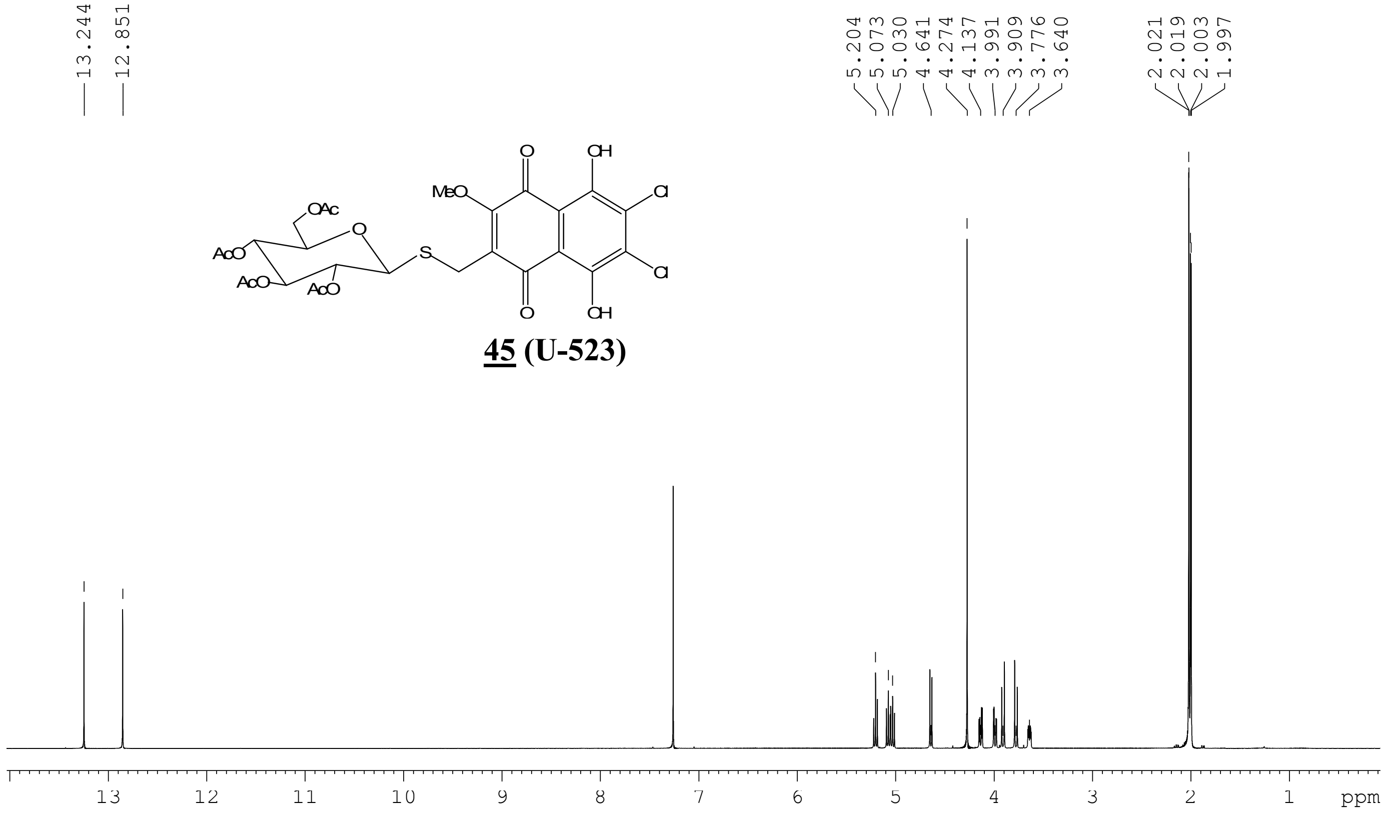

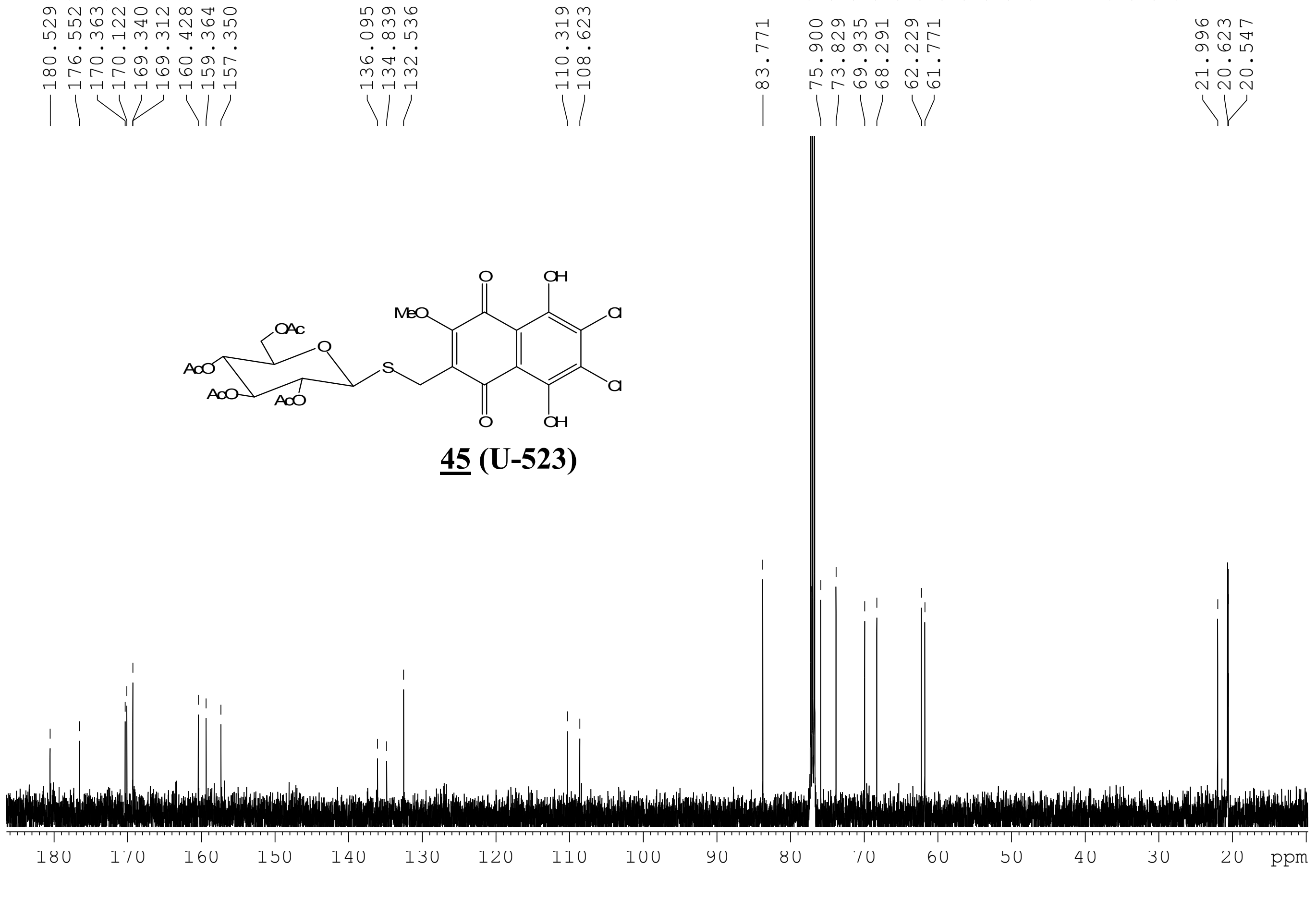

3-(Tetra-O-acetyl-β-D-glucopyranosyl-1-thiomethyl)-5,8-dihydroxy-2,7-dimethoxynaphthalene-1,4-dione **46** *(U-645),* (^{1}*H NMR - 700 MHz,* ^{13}*C NMR - 176 MHz, solvent – CDCl*$_3$*)*

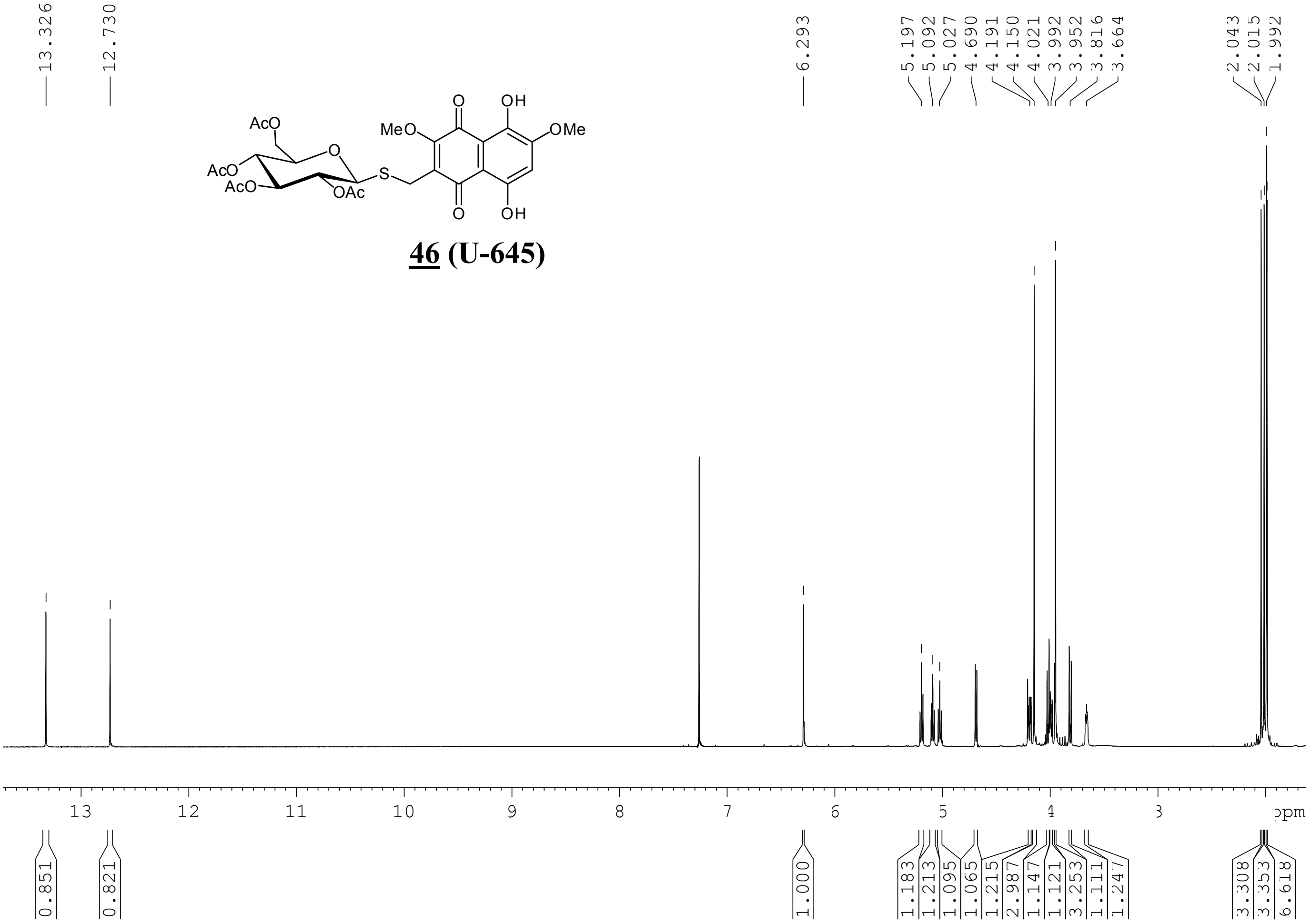

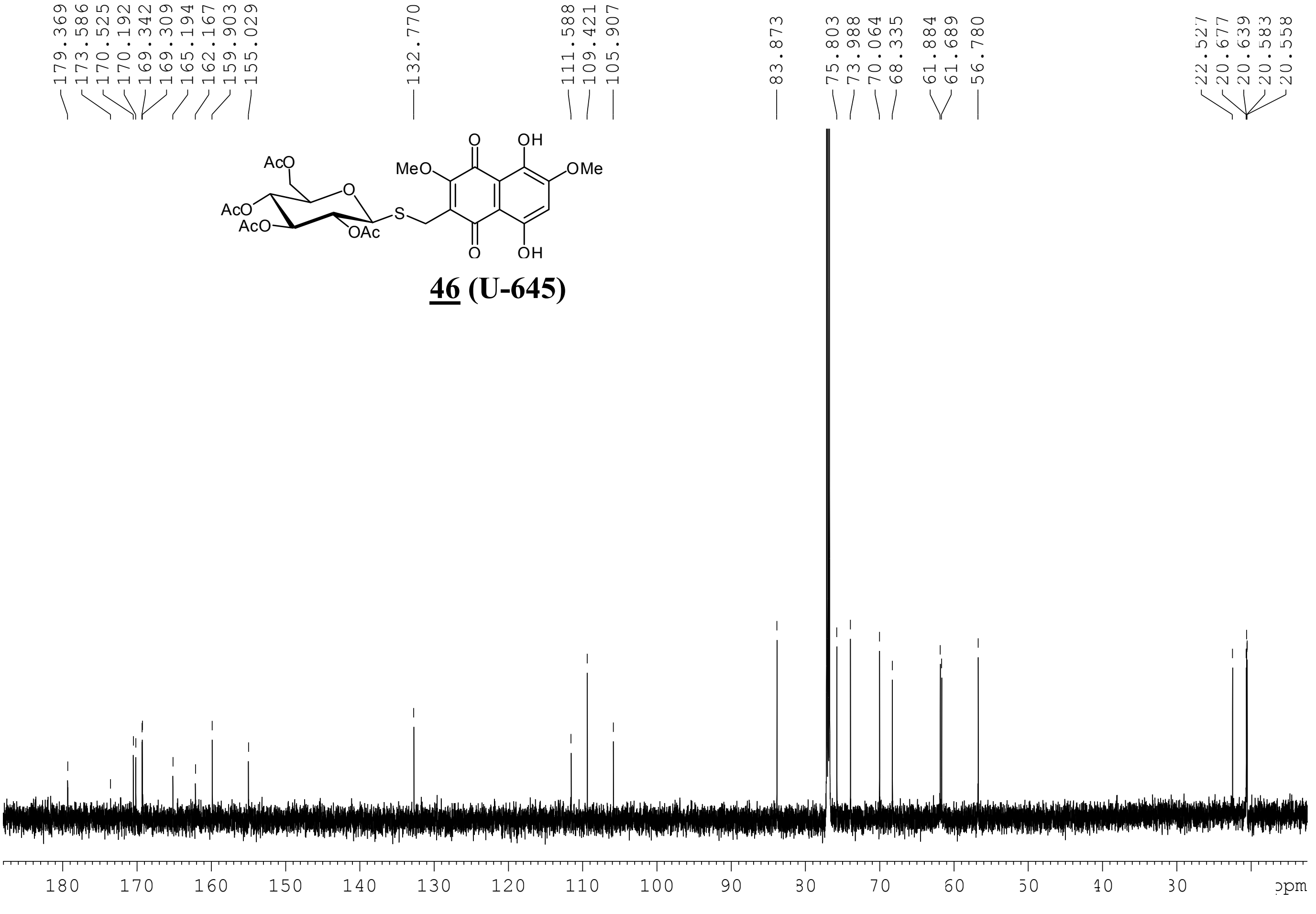

3-(Tetra-O-acetyl-β-D-glucopyranosyl-1-thiomethyl)-5,8-dihydroxy-2,6,7-trimethoxynaphthalene-1,4-dione ***47*** *(U-638)*, *(^{1}H NMR - 500 MHz, ^{13}C NMR - 125 MHz, solvent – $CDCl_3$)*

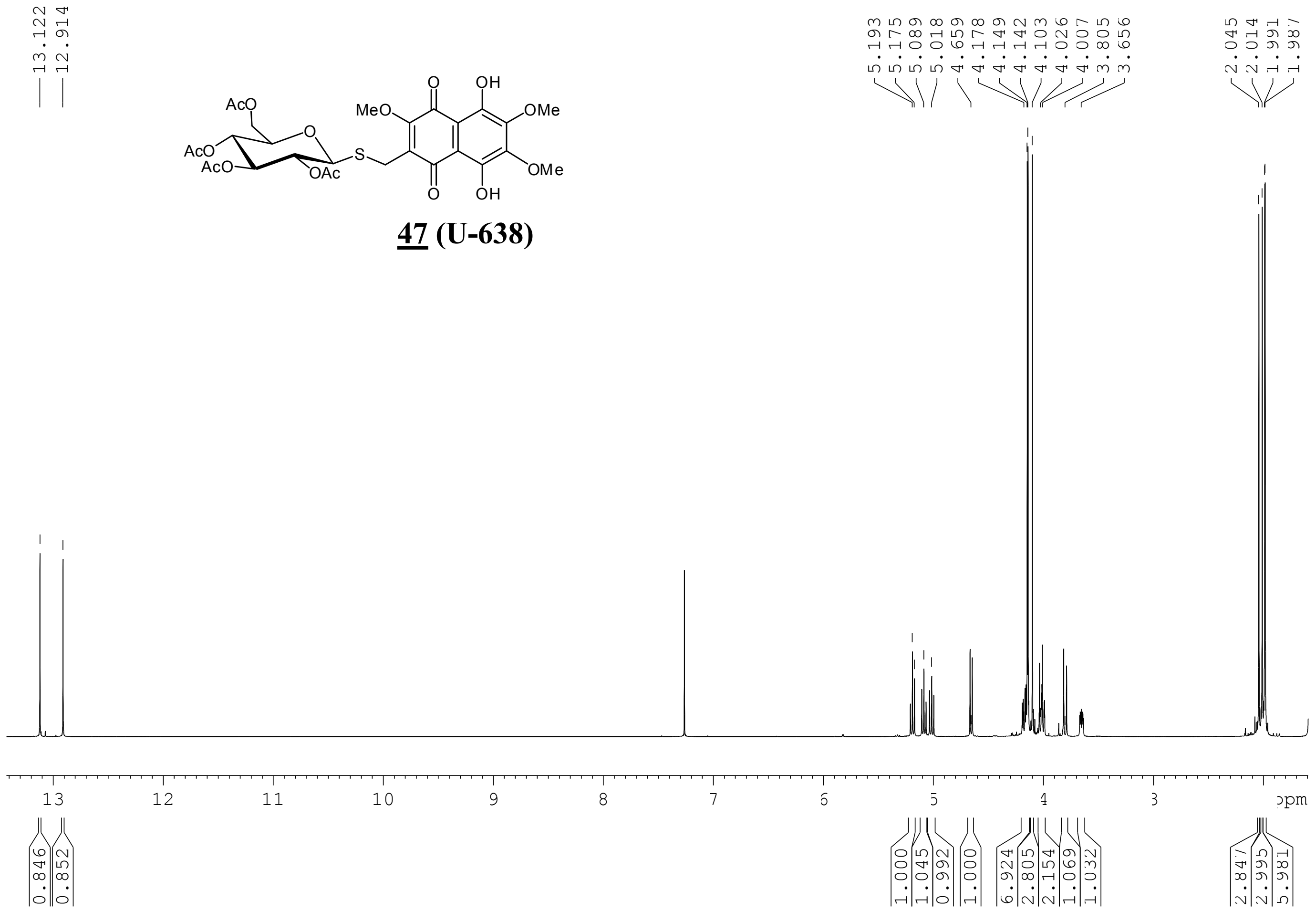

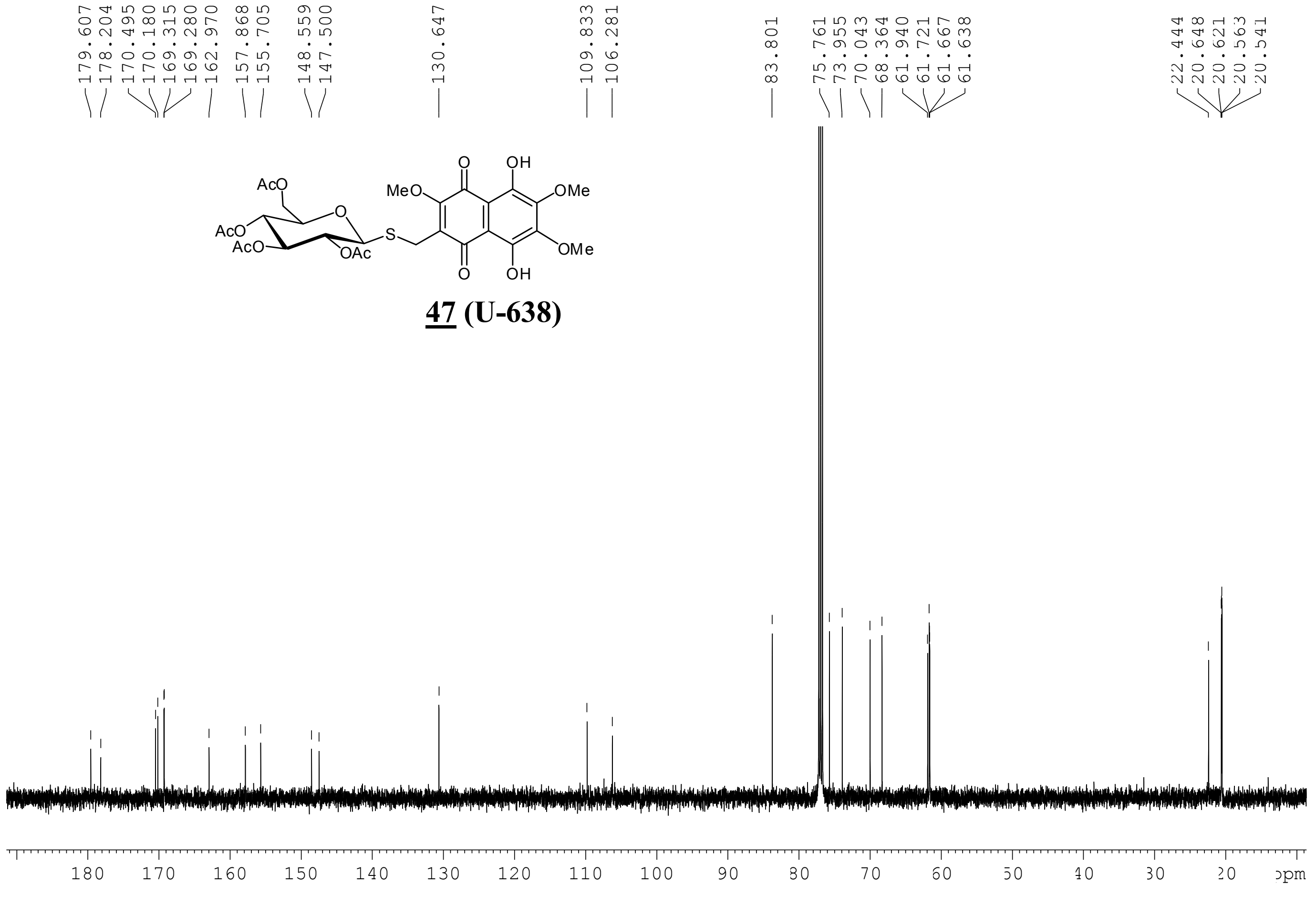

3-(β-D-Glucopyranosyl-1-thiomethyl)-5,8-dihydroxy-3-methoxynaphthalene-1,4-dione ***48*** *(U-636)*, *(¹H NMR - 500 MHz, ¹³C NMR - 125 MHz, solvent – DMSO-d₆)*

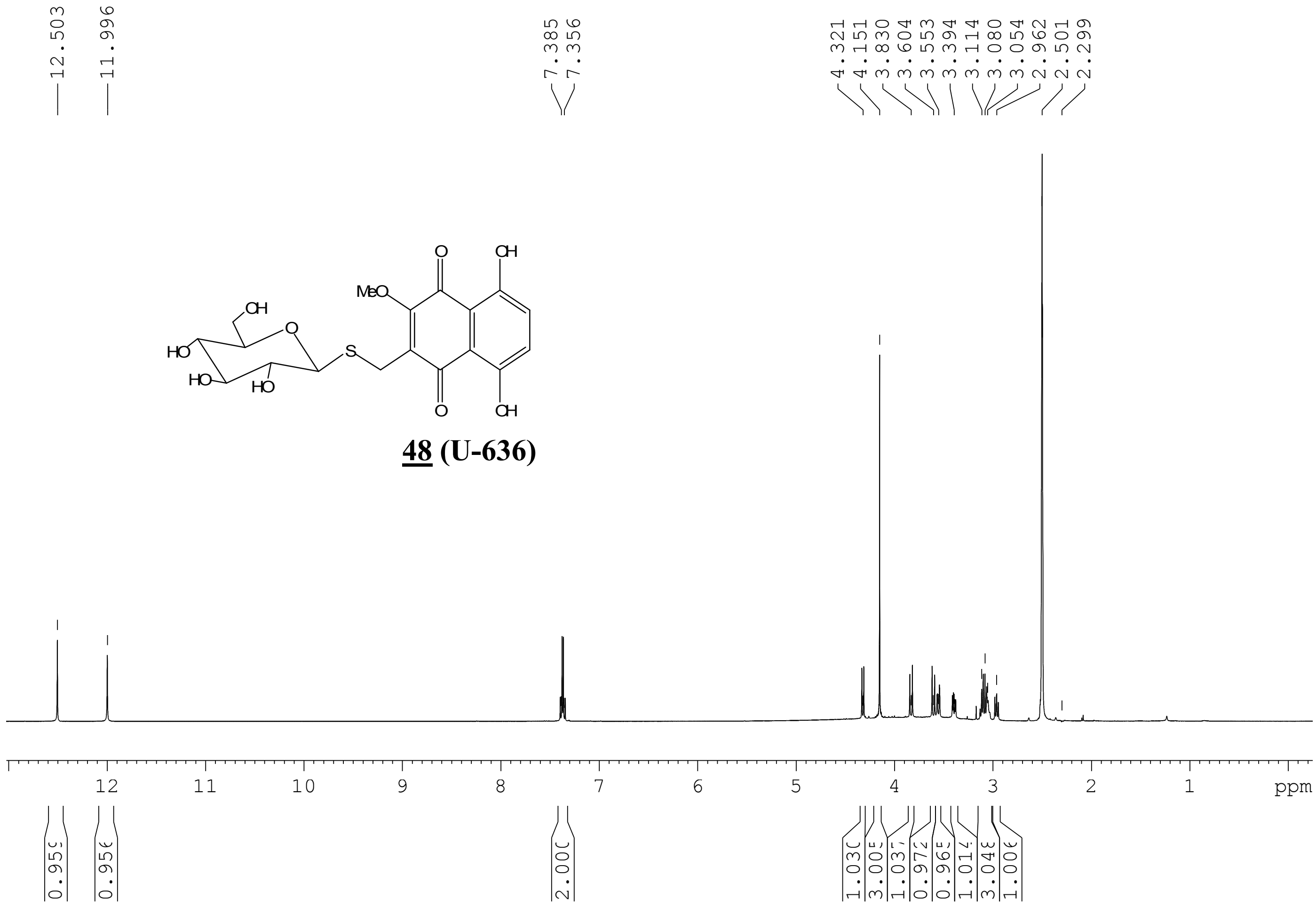

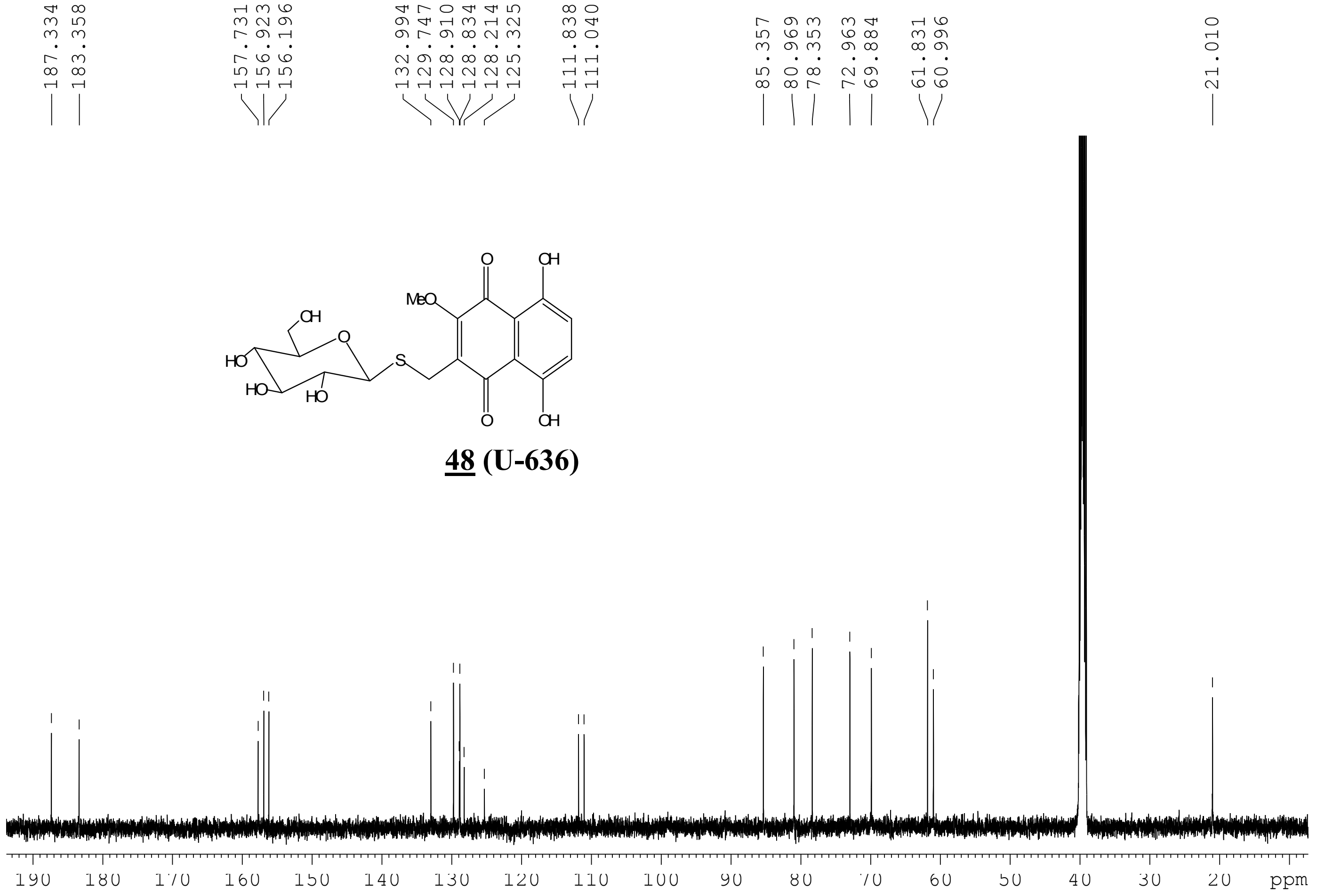

3-(β-D-Glucopyranosyl-1-thiomethyl)-5,8-dihydroxy-2-methoxy-6,7-dimethylnaphthalene-1,4-dione ***49*** *(U-522)*, (^{1}*H NMR - 700 MHz,* ^{13}*C NMR - 176 MHz, solvent – DMSO-d*$_6$)

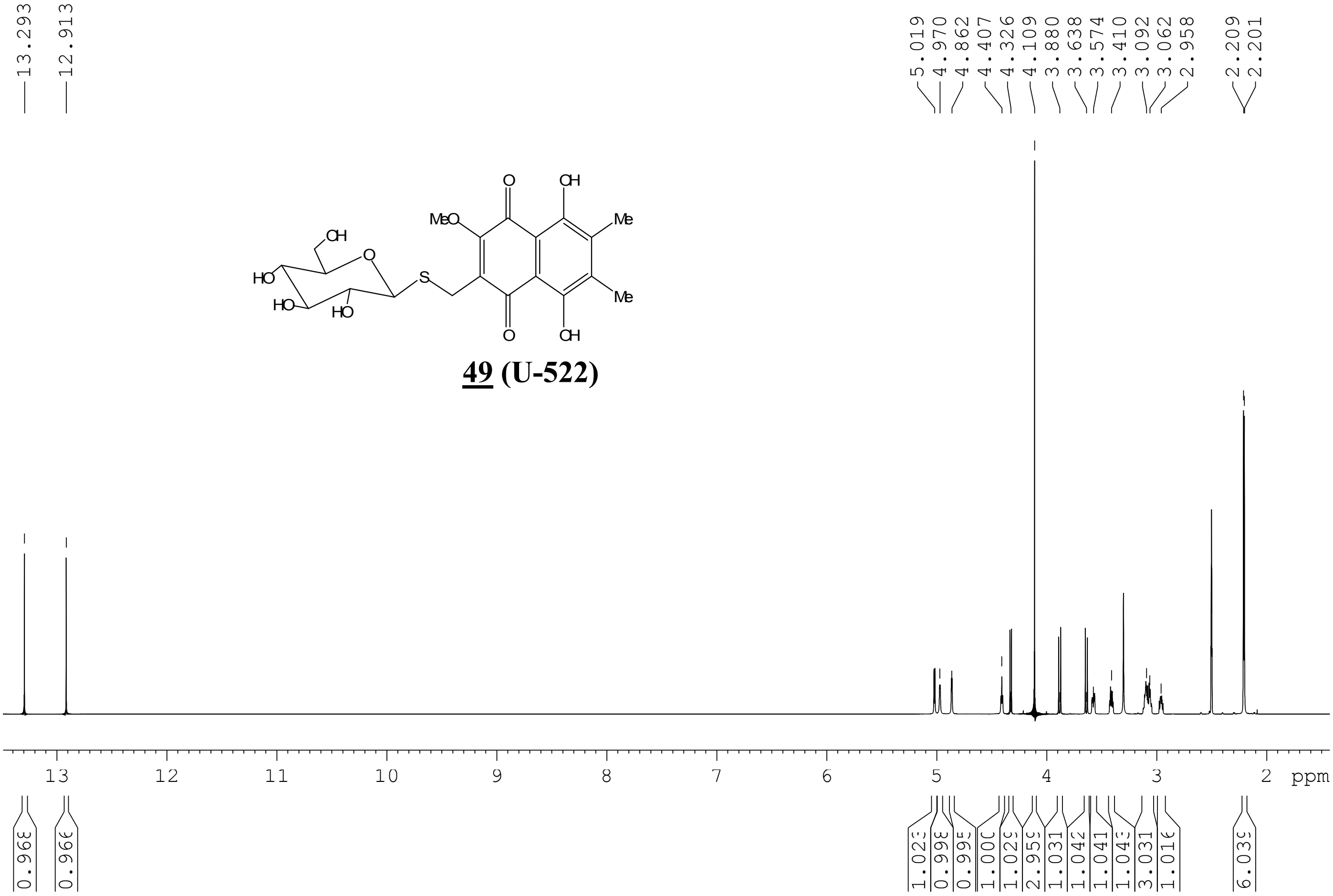

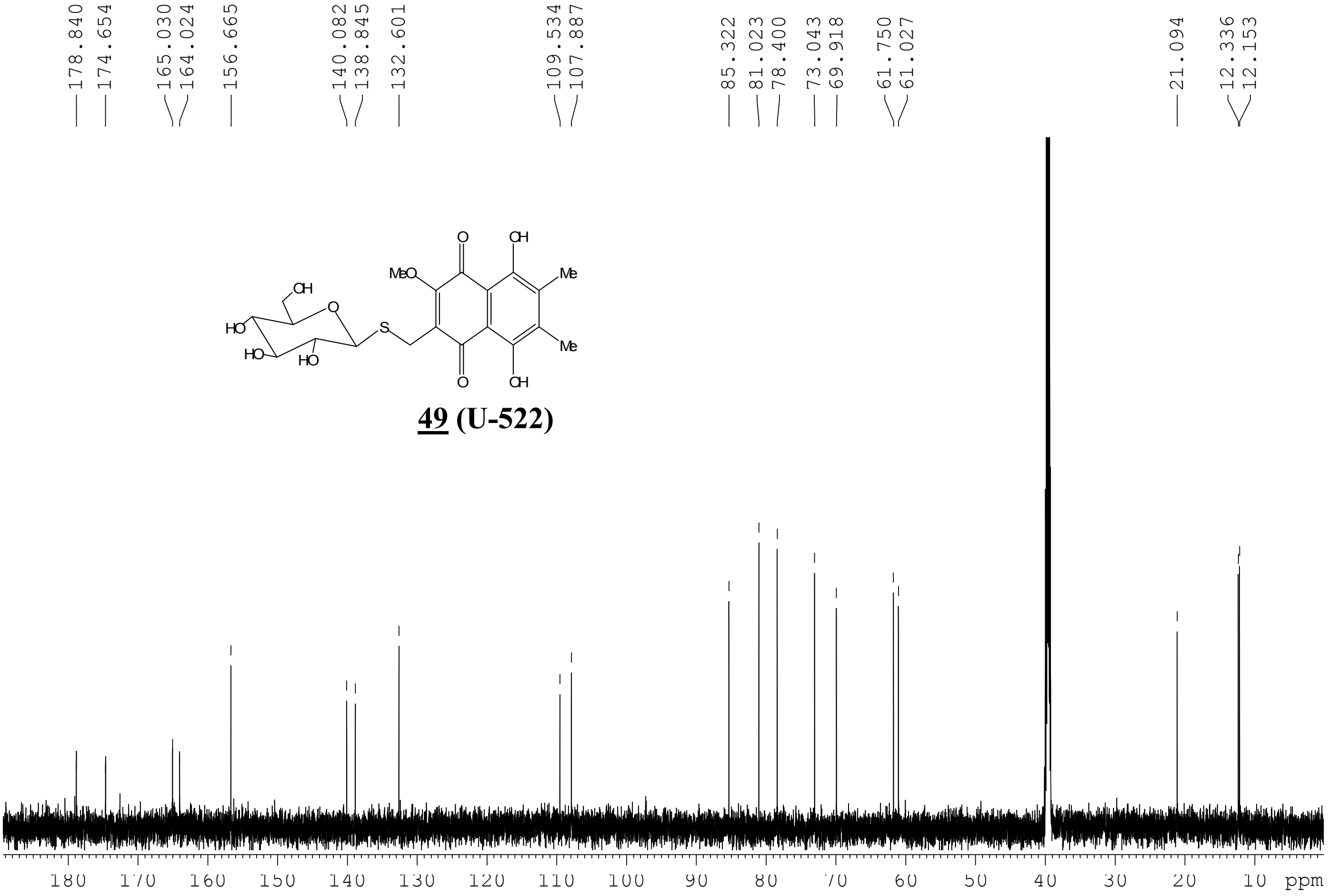

6,7-Dichloro-3-(β-D-glucopyranosyl-1-thiomethyl)-5,8-dihydroxy-2-methoxynaphthalene-1,4-dione ***50*** *(U-625)*, *(^{1}H NMR - 500 MHz, ^{13}C NMR - 125 MHz, solvent – DMSO-d6)*

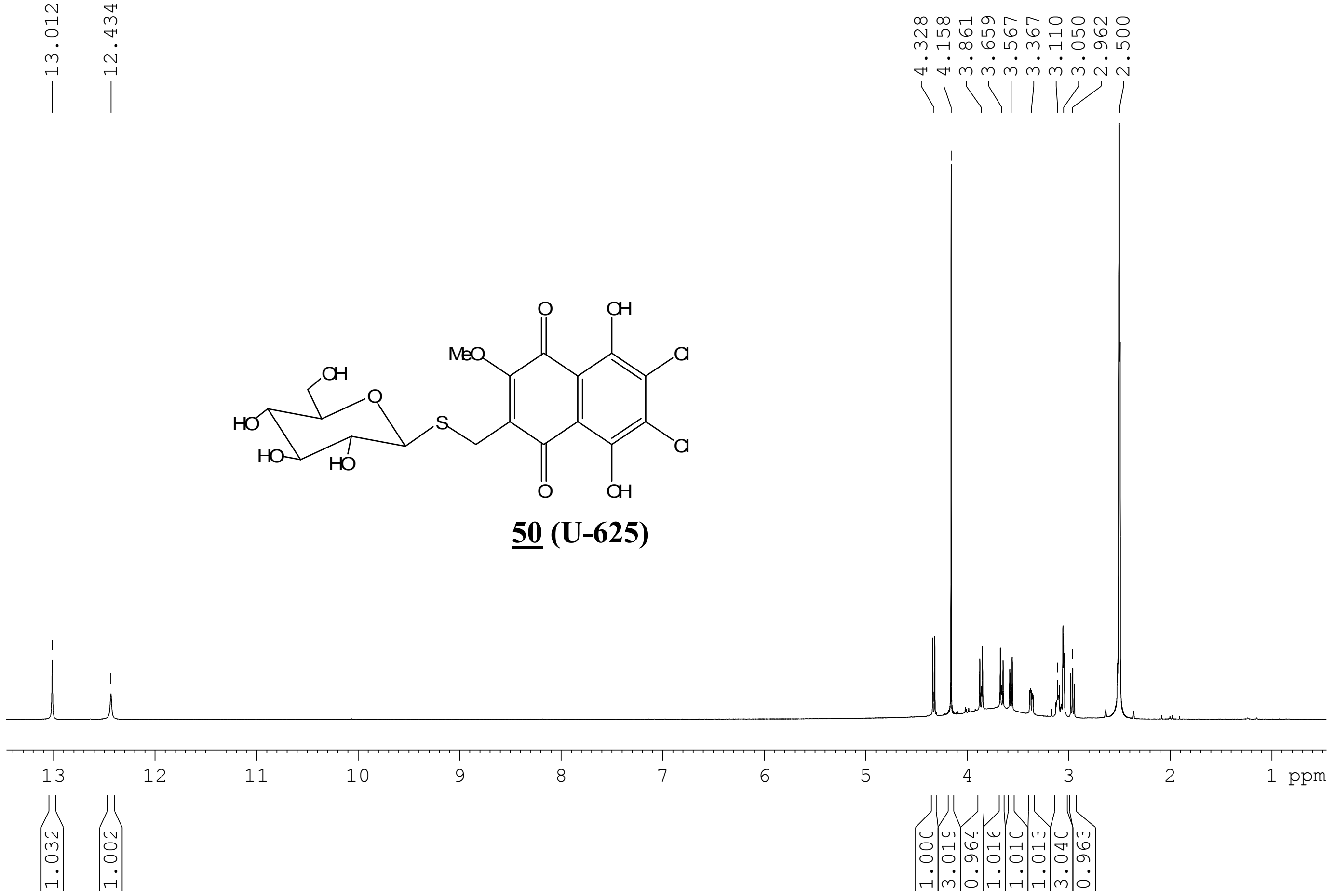

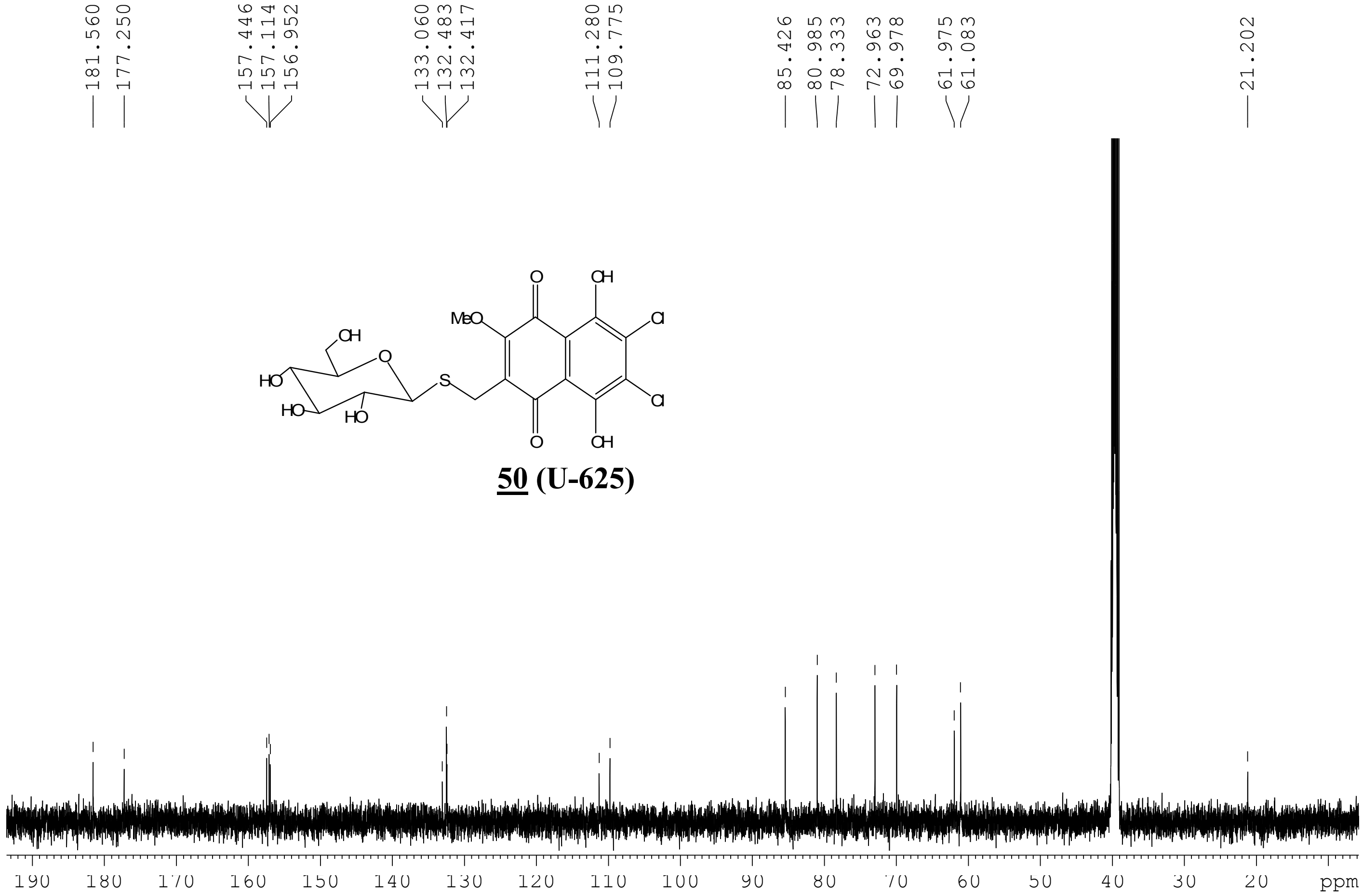

3-(β-D-Glucopyranosyl-1-thiomethyl)-5,8-dihydroxy-2,7-dimethoxynaphthalene-1,4-dione ***51*** *(U-646)*, *(^{1}H NMR - 500 MHz, ^{13}C NMR - 125 MHz, solvent – DMSO-d_6)*

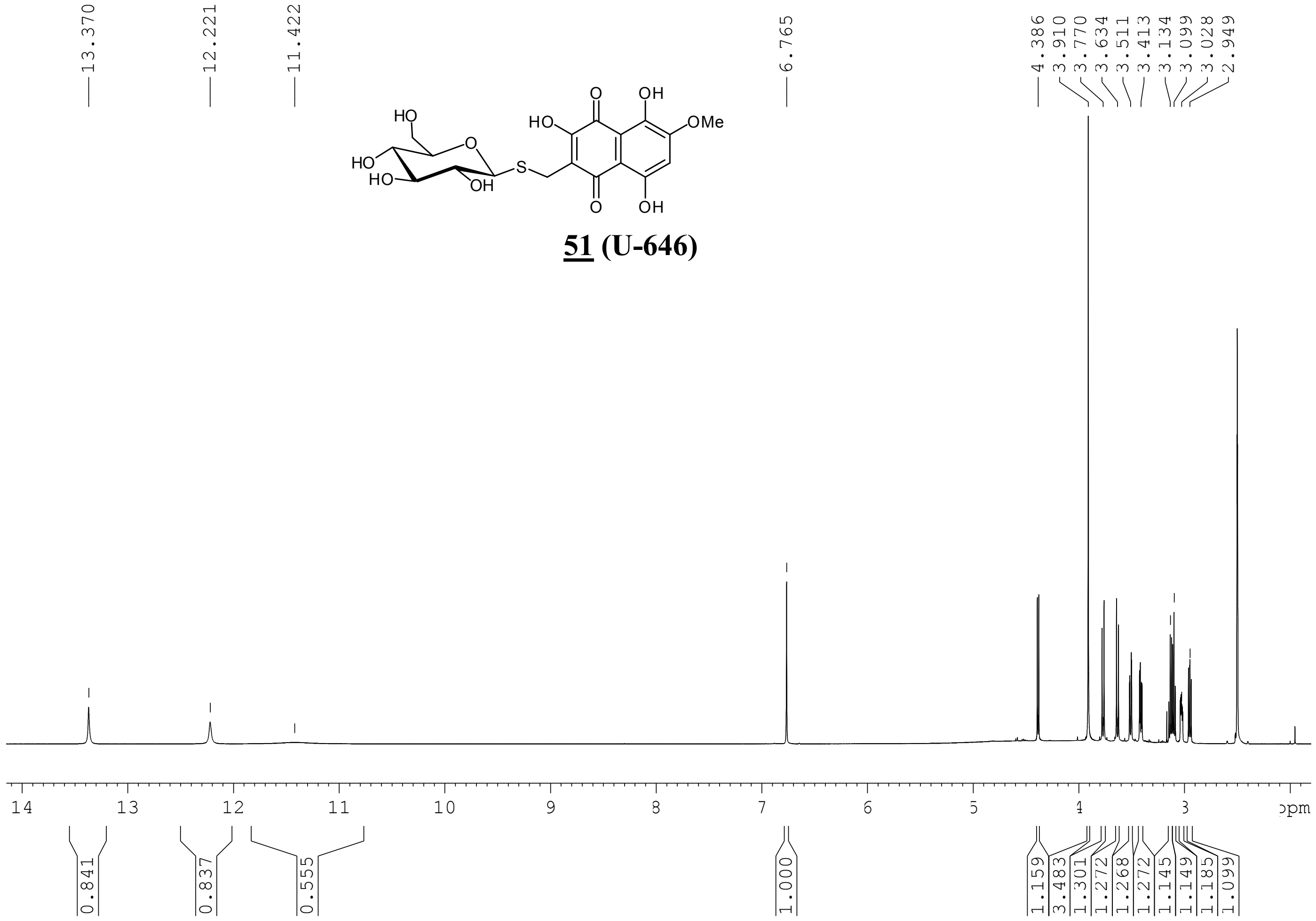

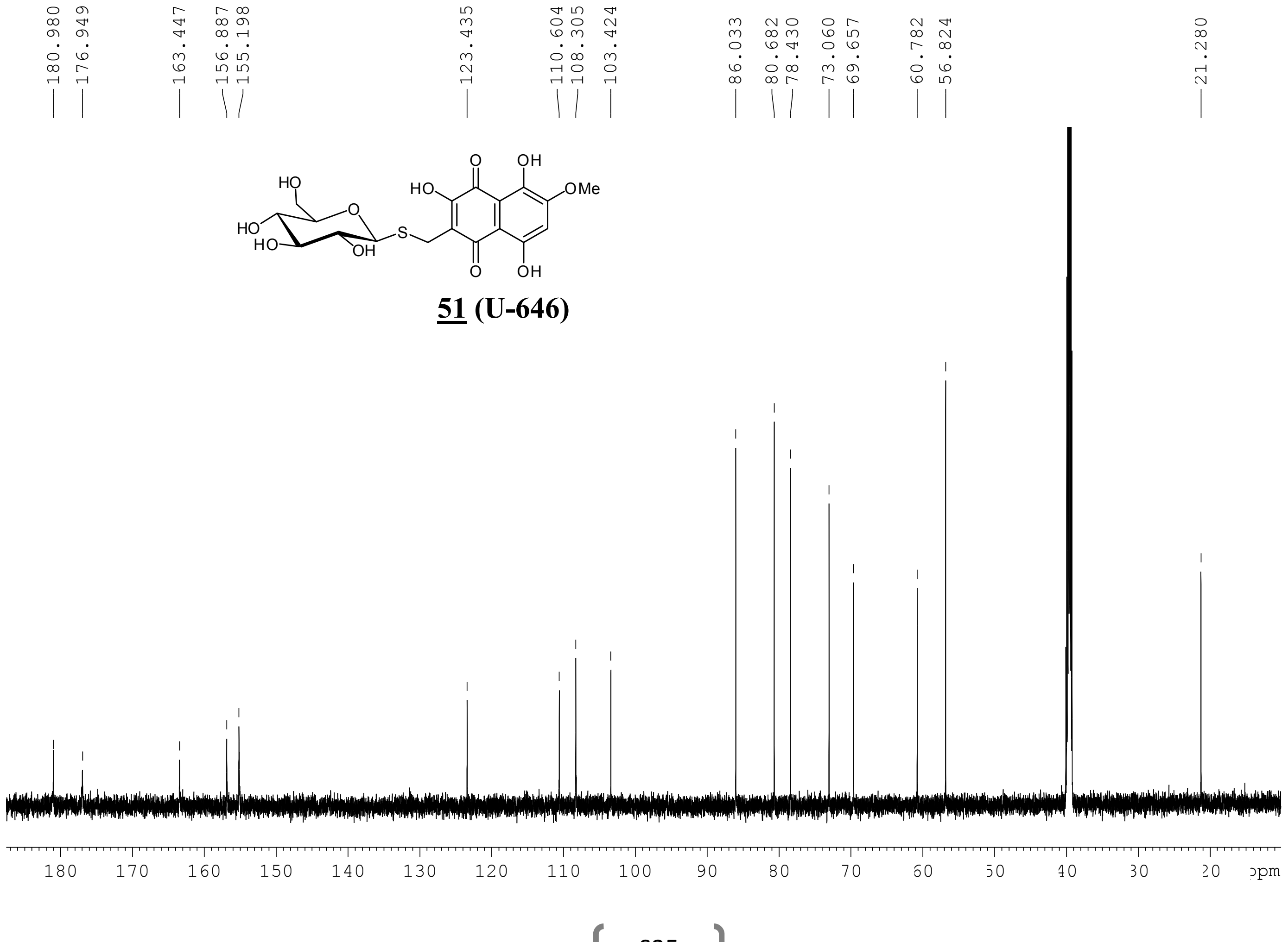

3-(β-D-Glucopyranosyl-1-thiomethyl)-5,8-dihydroxy-2,6,7-trimethoxynaphthalene-1,4-dione **52** *(U-641)*, *(^{1}H NMR - 700 MHz, ^{13}C NMR - 176 MHz, solvent – DMSO-d_6)*

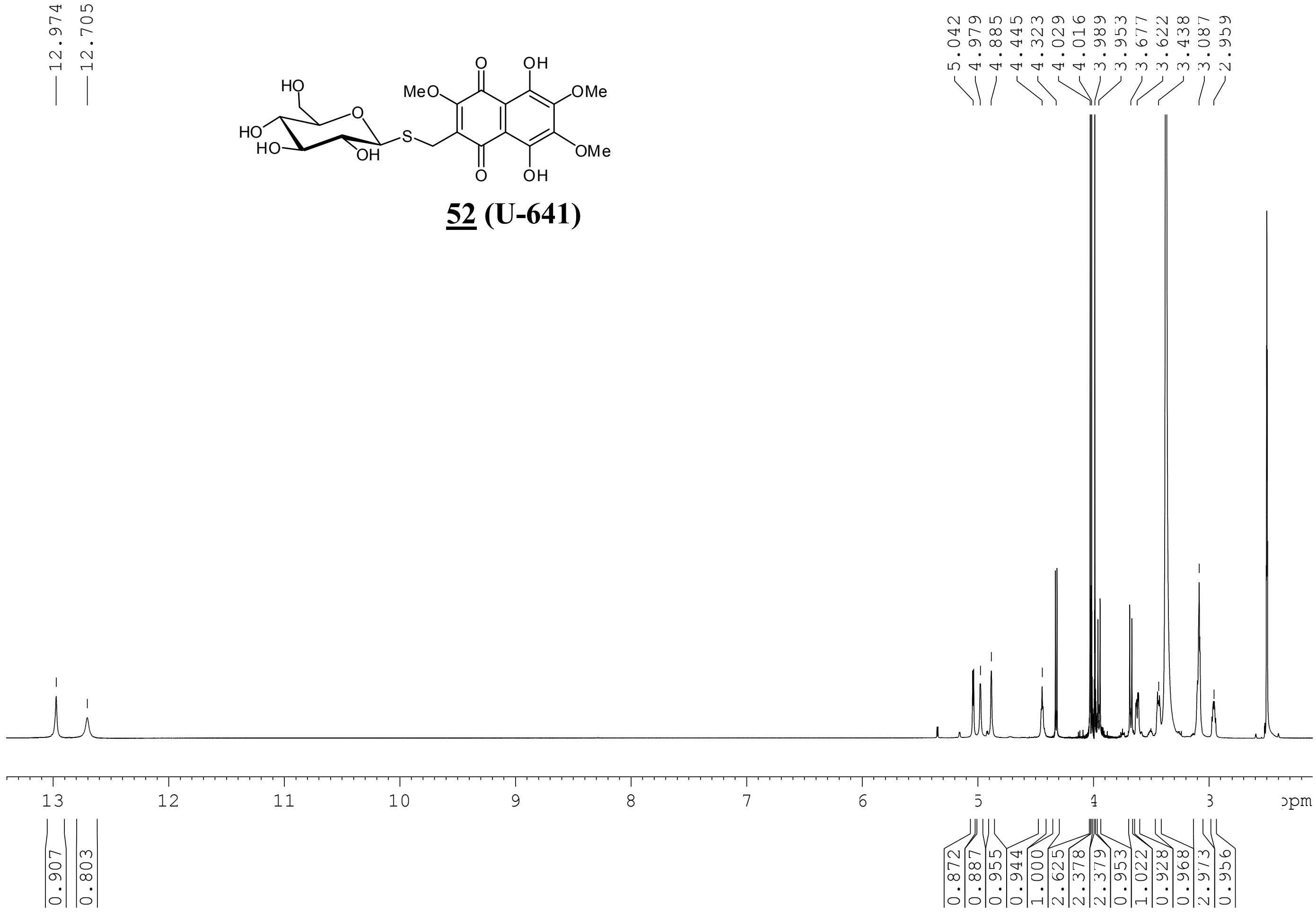

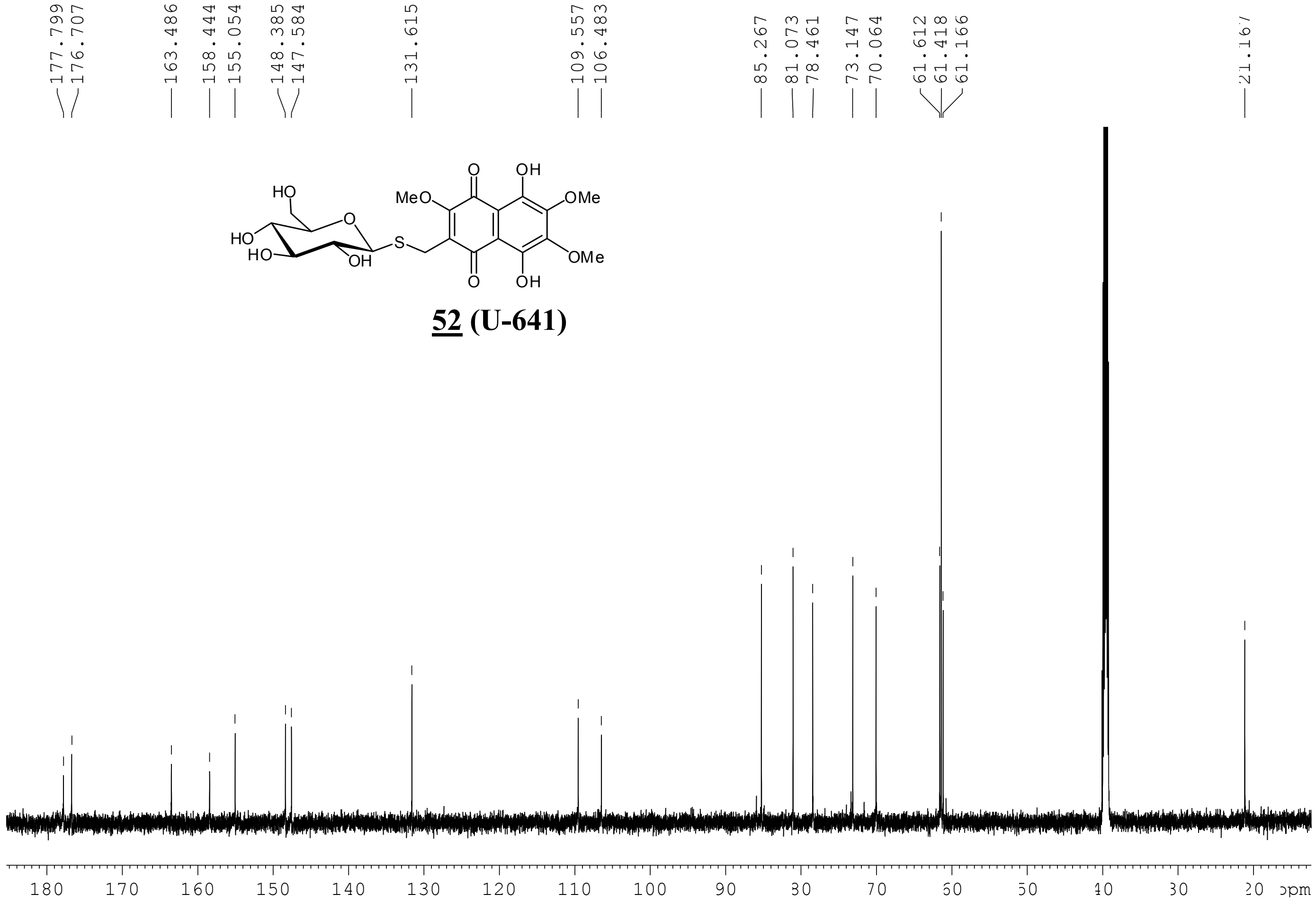

3-(Tetra-O-acetyl-β-D-glucopyranosyl-1-thiomethyl)-2,5,6,7,8-pentahydroxynaphthalene-1,4-dione ***56*** *(U-629)***,** *(^{1}H NMR - 700 MHz, ^{13}C NMR - 176 MHz, solvent – $CDCl_3$)*

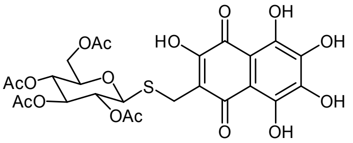

56 (U-629)

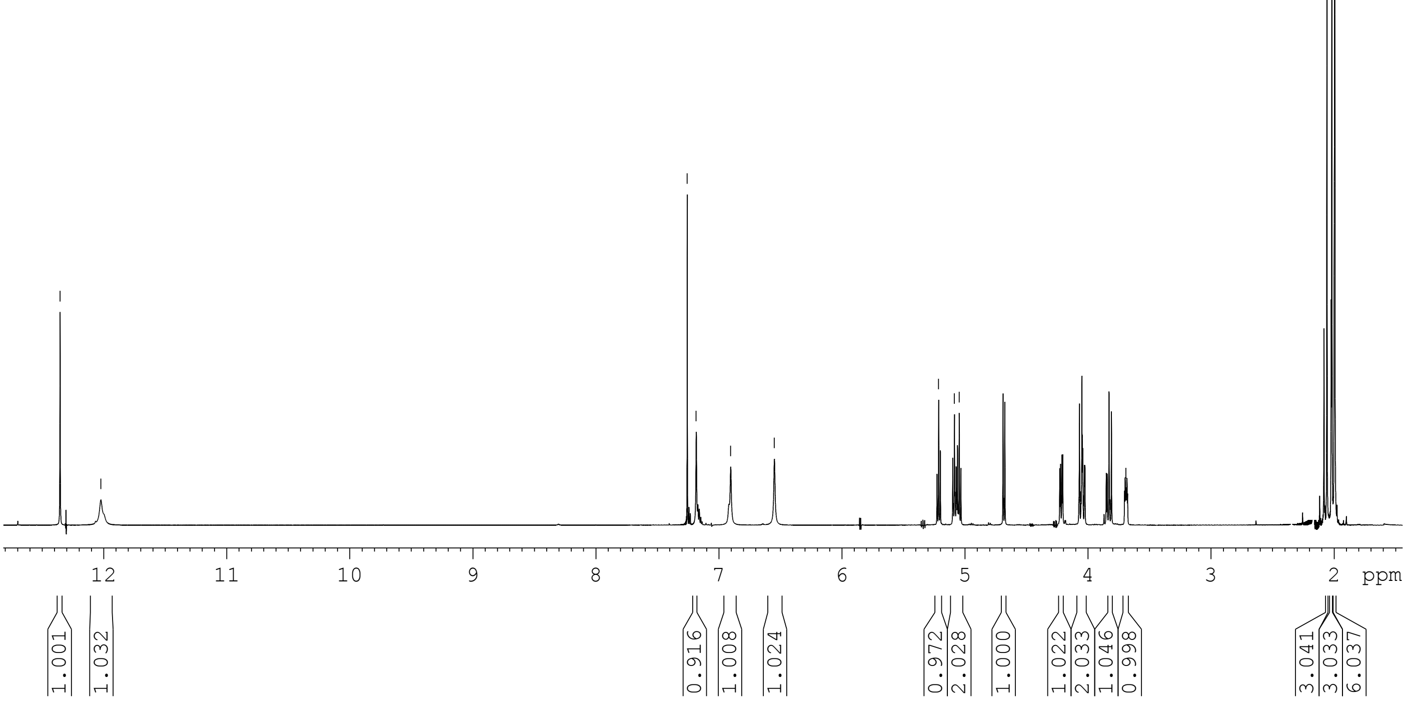

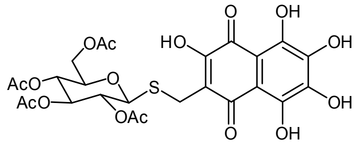

56 (U-629)

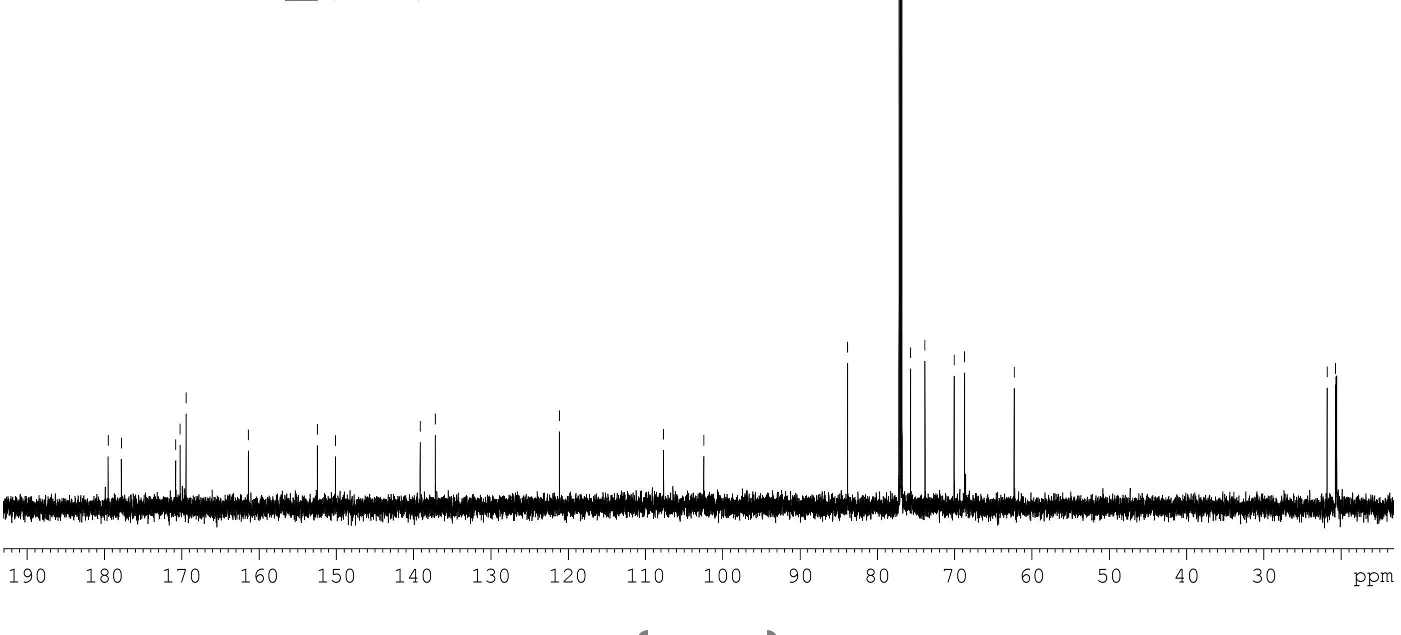

3-(Tetra-O-acetyl-β-D-galactopyranosyl-1-thiomethyl)-2,5,6,7,8-pentahydroxynaphthalene-1,4-dione ***57*** *(U-631)*, *(^{1}H NMR - 700 MHz, ^{13}C NMR - 176 MHz, solvent – DMSO-d$_6$)*

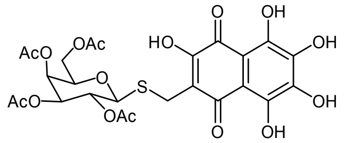

57 (U-631)

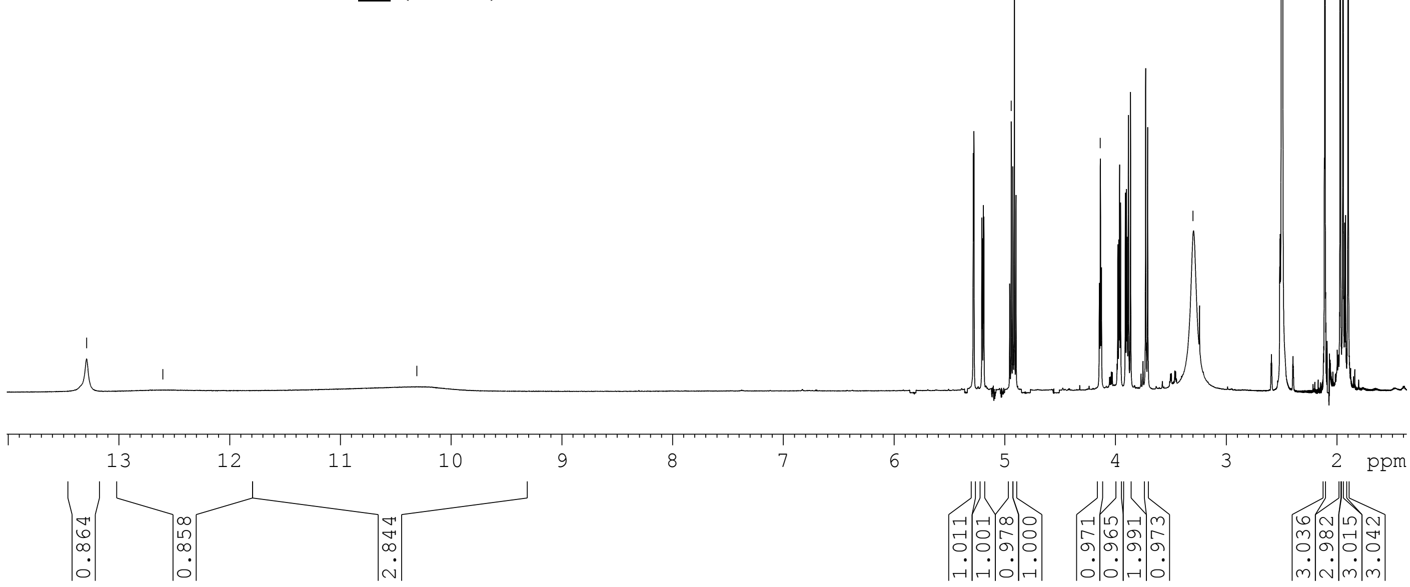

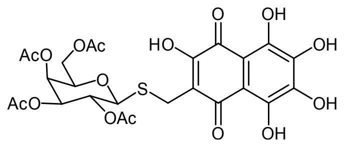

57 (U-631)

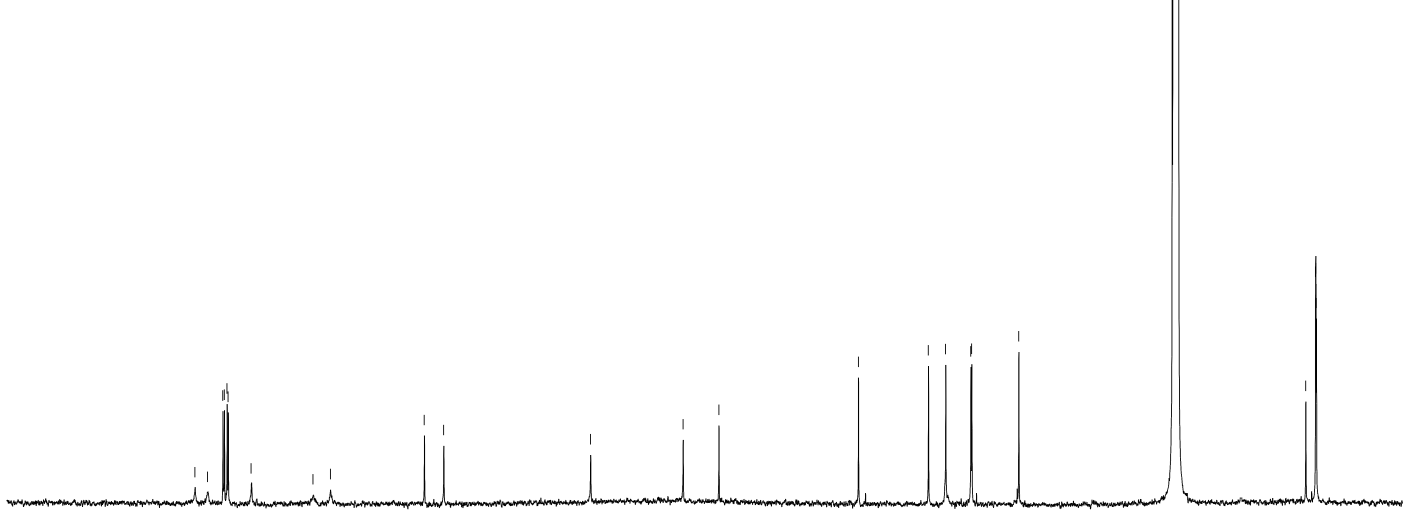

*3-(Tetra-O-acetyl-β-D-mannopyranosyl-1-thiomethyl)-2,5,6,7,8-pentahydroxynaphthalene-1,4-dione **58** (U-630)*, *(^{1}H NMR - 500 MHz, ^{13}C NMR - 125 MHz, solvent – $CDCl_3$)*

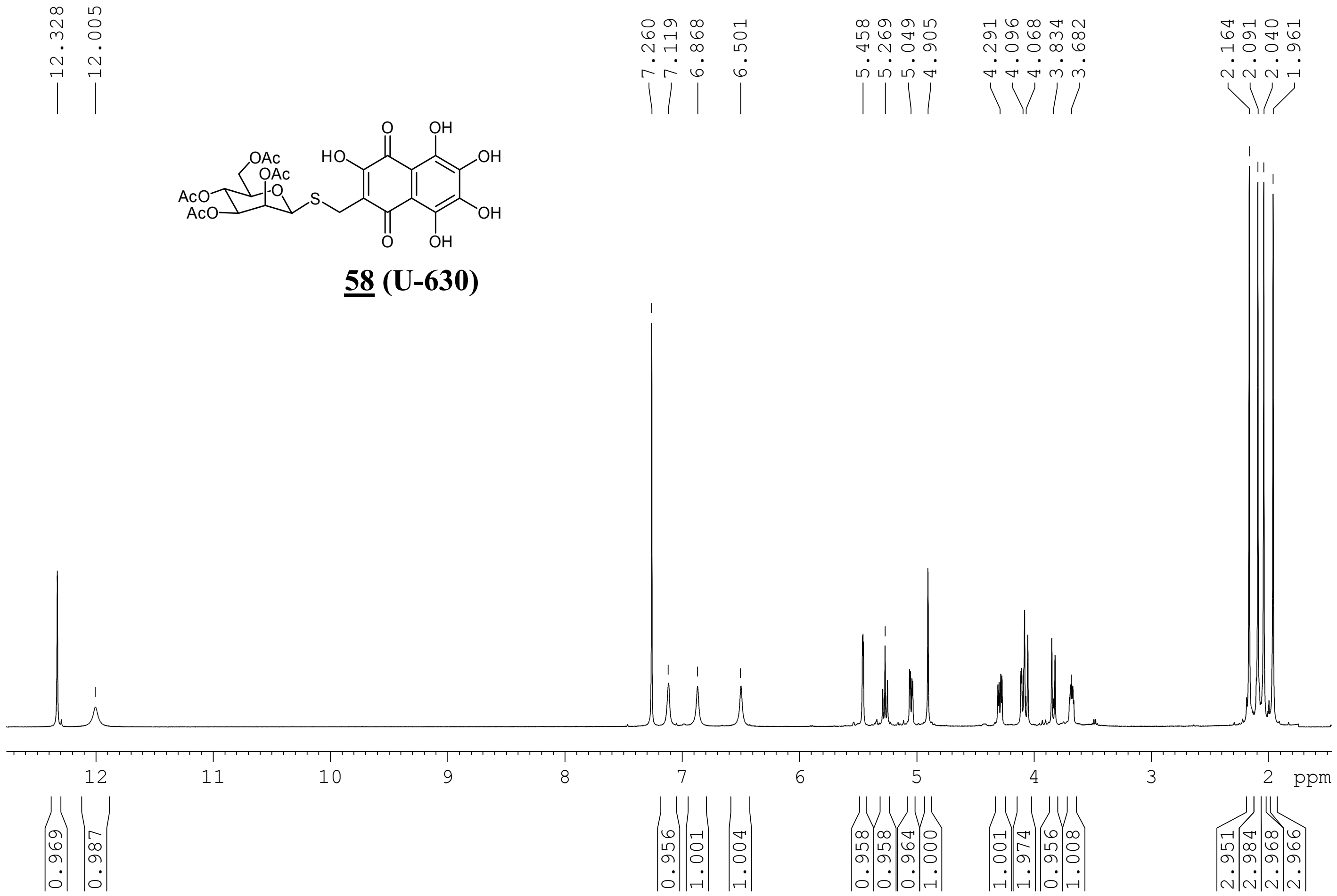

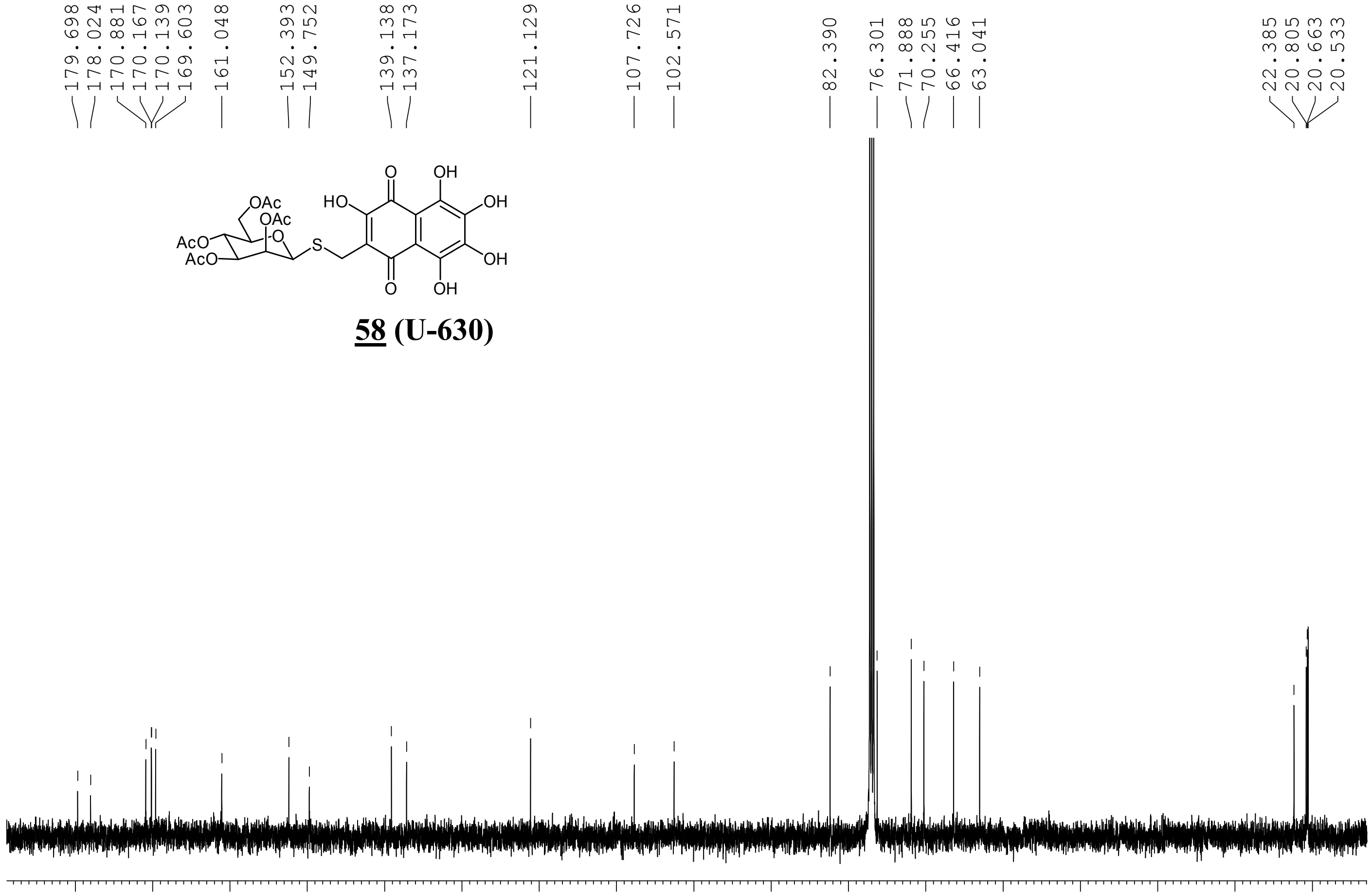

3-(Tri-O-acetyl-β-D-xylopyranosyl-1-thiomethyl)-2,5,6,7,8-pentahydroxynaphthalene-1,4-dione ***59*** *(U-628)*, (*^{1}H NMR - 500 MHz, ^{13}C NMR - 125 MHz, solvent – DMSO-d$_6$*)

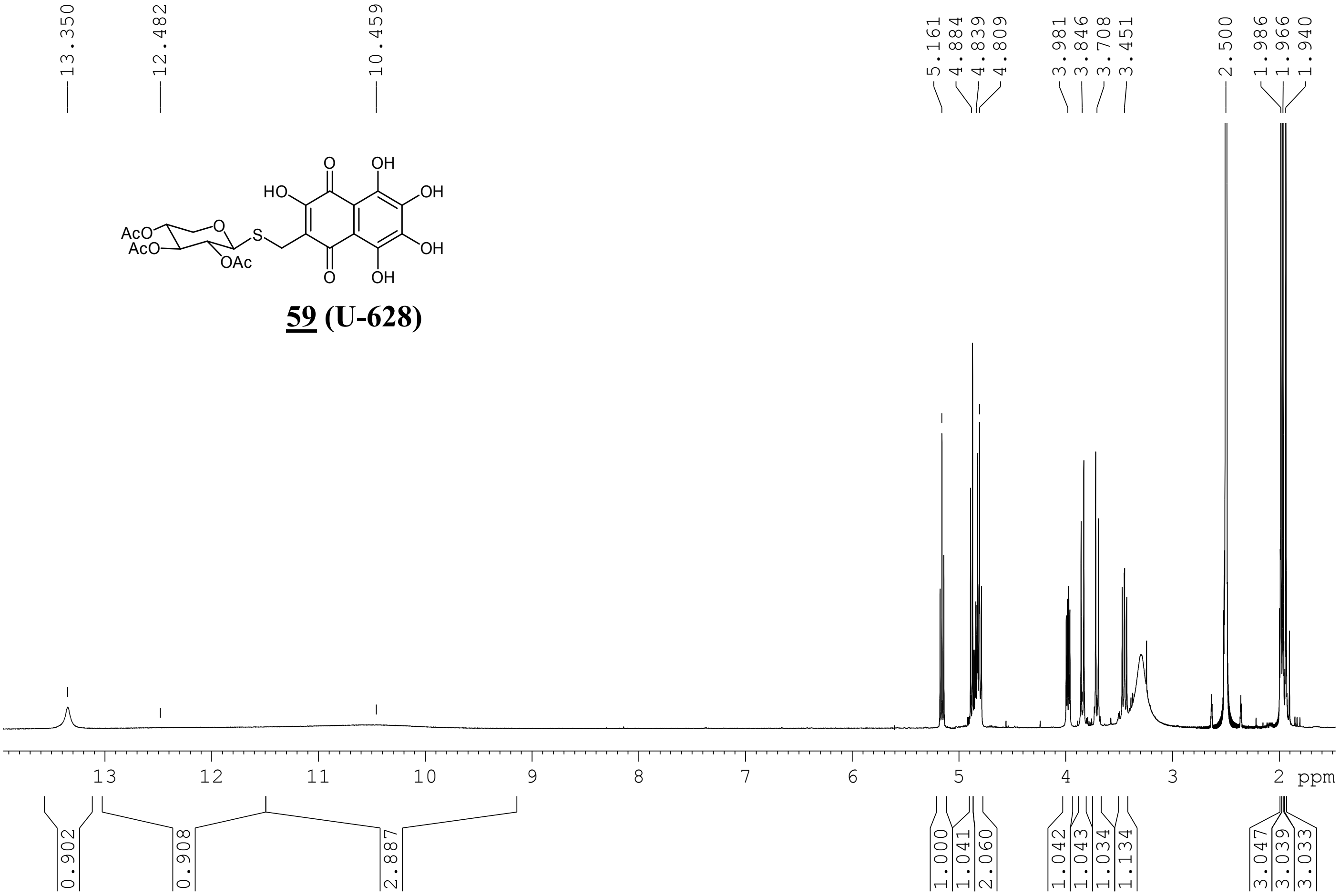

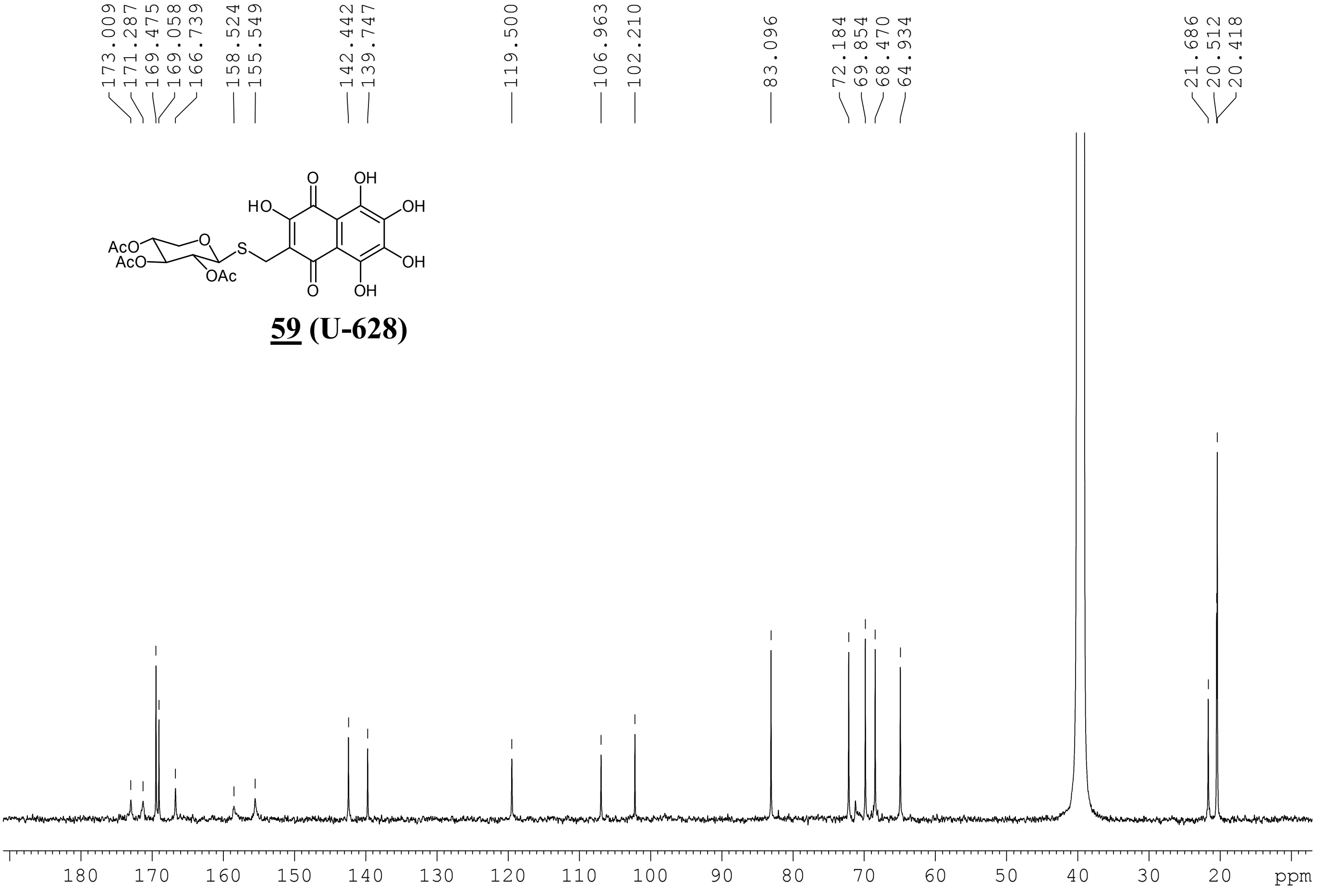

*3-(β-D-Glucopyranosyl-1-thiomethyl)-2,5,6,7,8-pentahydroxynaphthalene-1,4-dione **60** (U-649)*, *(^{1}H NMR - 700 MHz, ^{13}C NMR - 176 MHz, solvent – DMSO-d_6)*

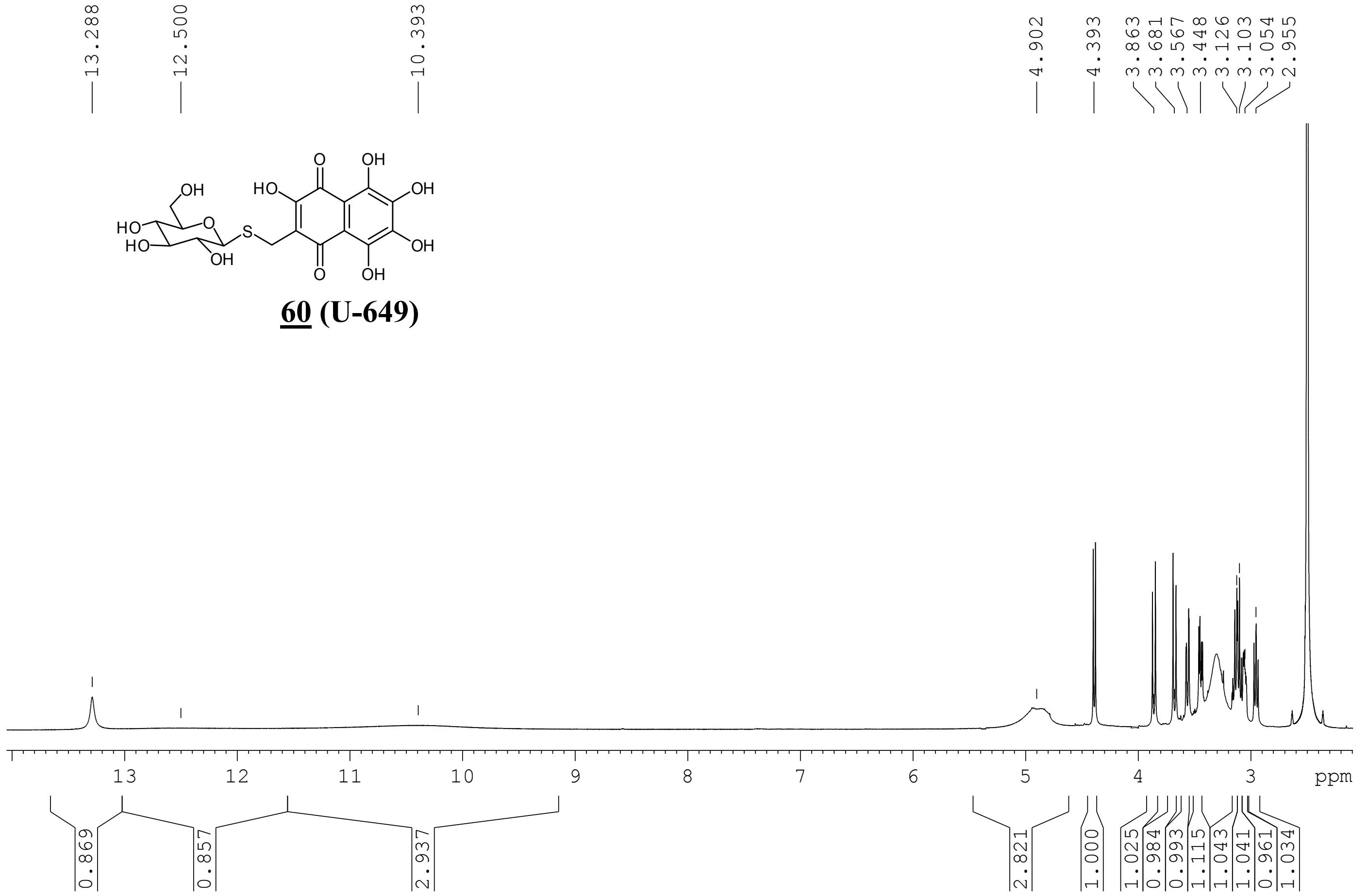

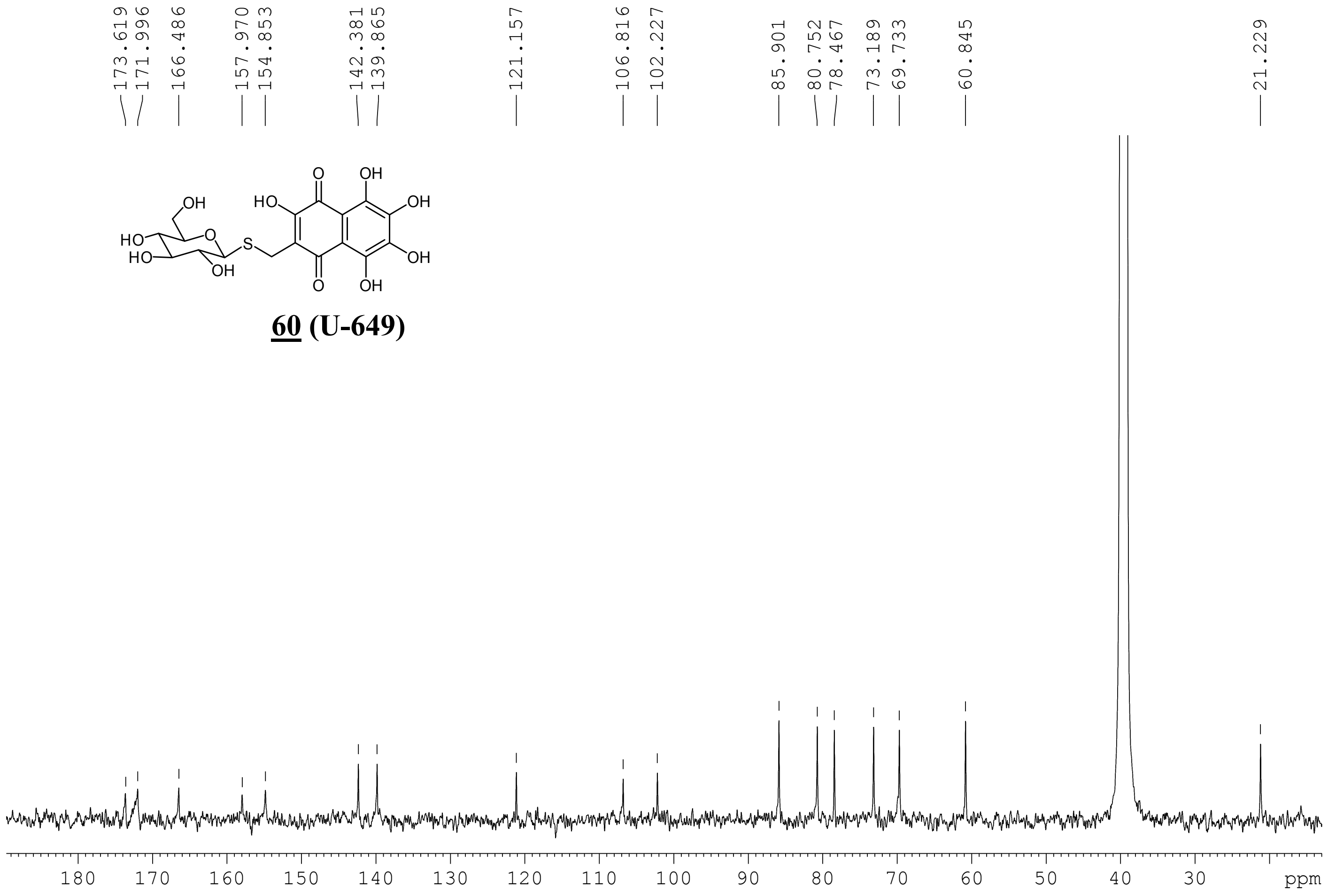

3-(β-D-Galactopyranosyl-1-thiomethyl)-2,5,6,7,8-pentahydroxynaphthalene-1,4-dione ***61*** *(U-650)*, *(^{1}H NMR - 500 MHz, ^{13}C NMR - 125 MHz, solvent – DMSO-d$_6$)*

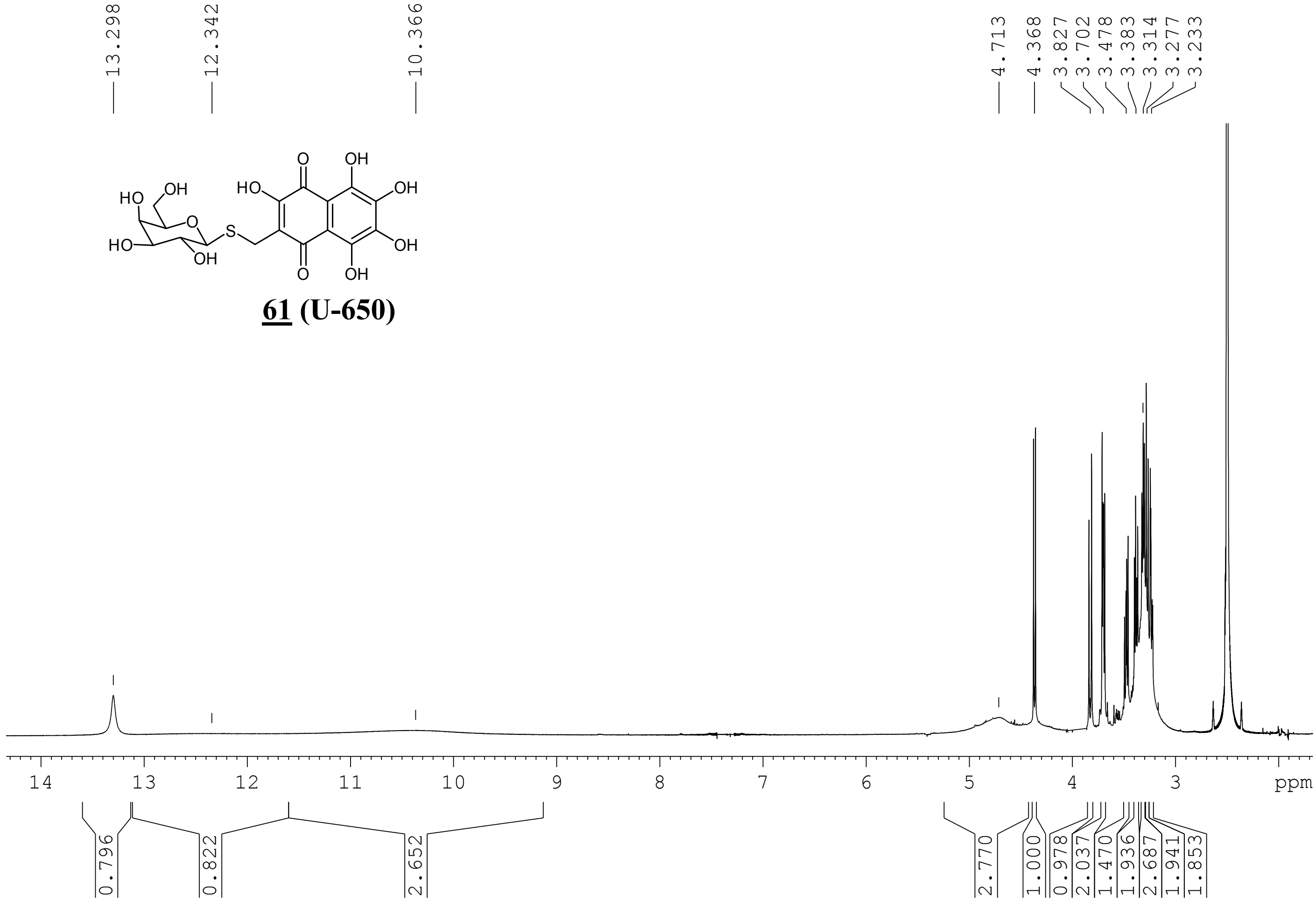

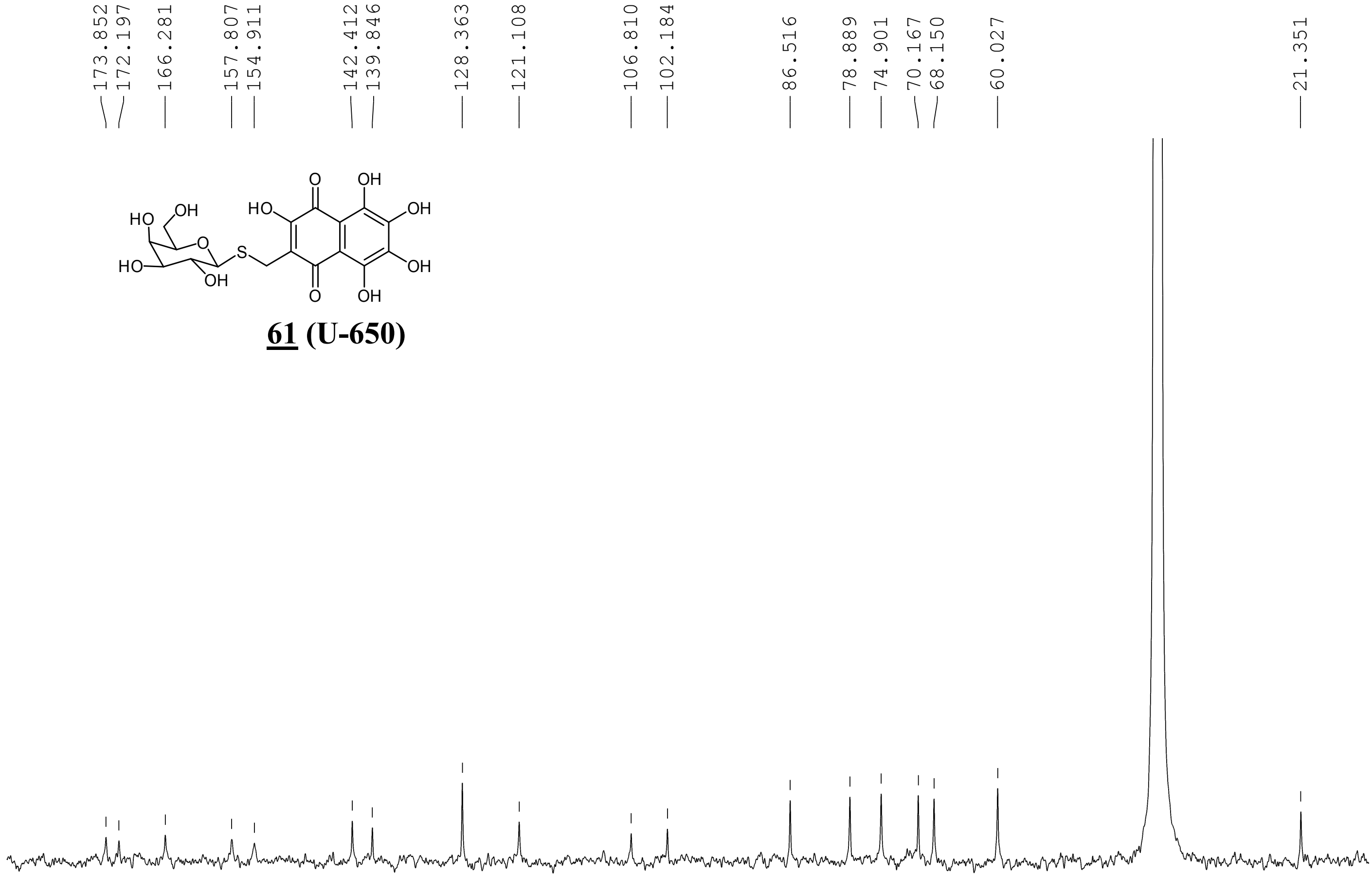

*3-(β-D-Mannopyranosyl-1-thiomethyl)-2,5,6,7,8-pentahydroxynaphthalene-1,4-dione **62** (U-648), (^1H NMR - 500 MHz, ^{13}C NMR - 125 MHz, solvent – DMSO-d_6)*

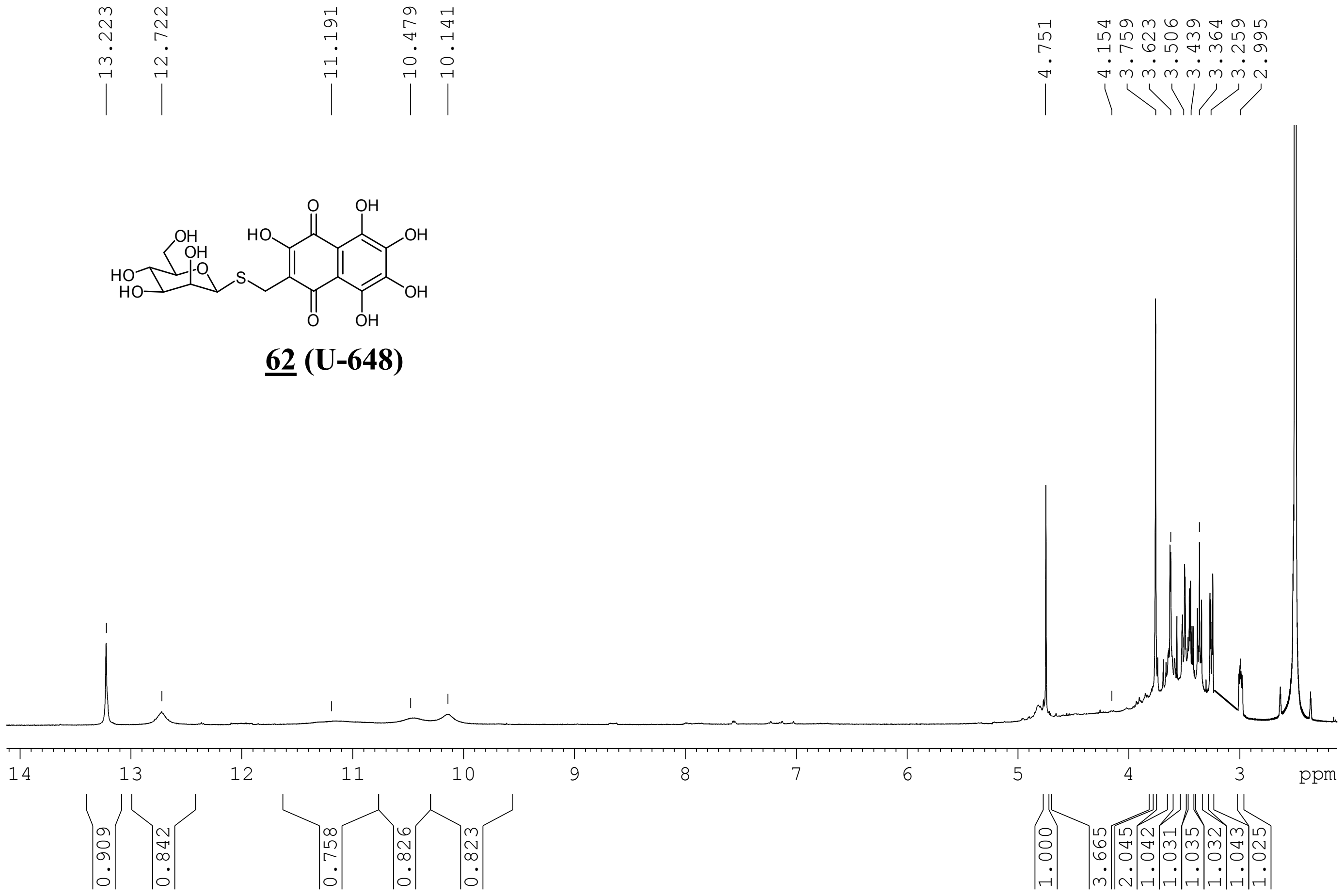

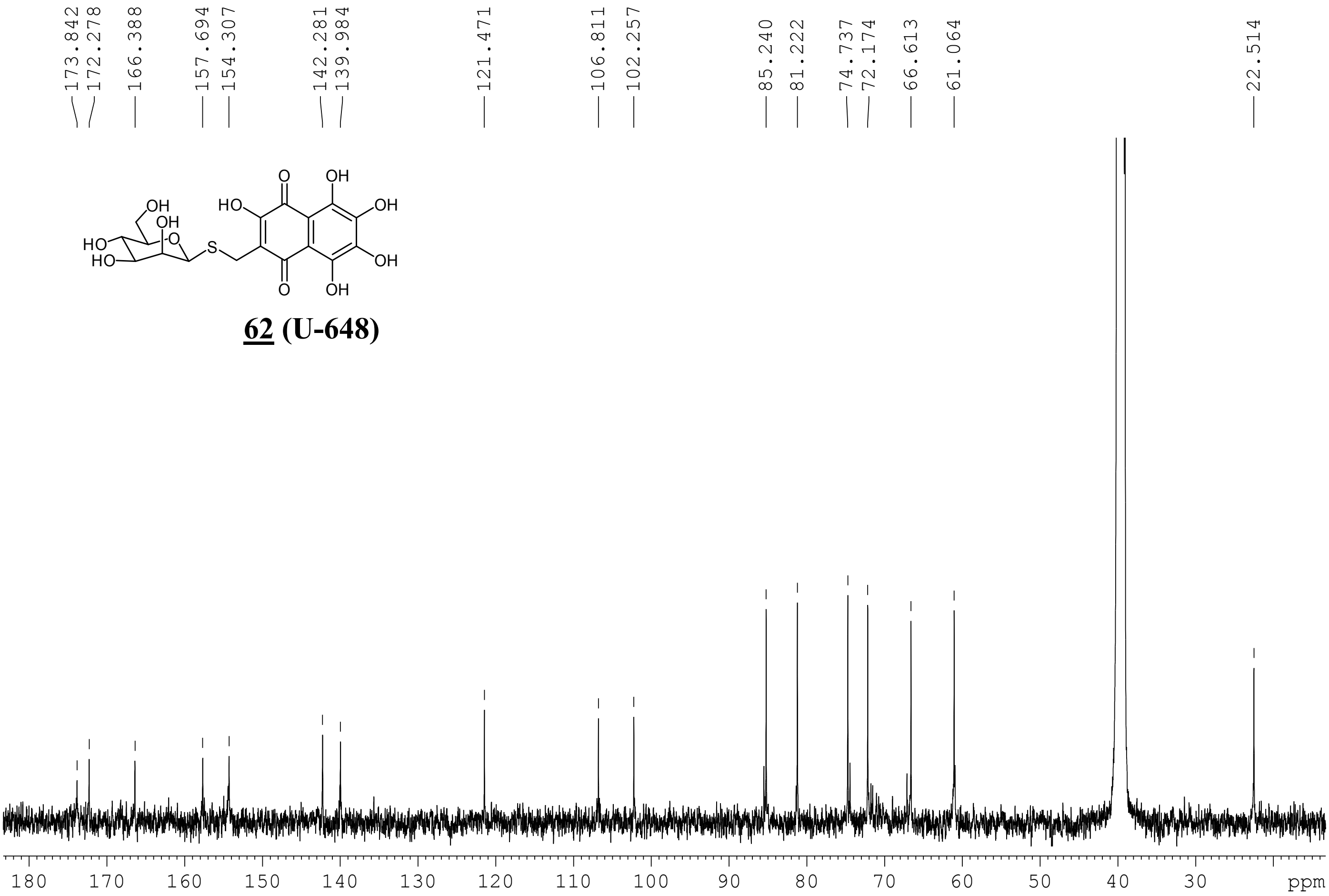

*3-(β-D-Xylopyranosyl-1-thiomethyl)-2,5,6,7,8-pentahydroxynaphthalene-1,4-dione **63** (U-647), (^{1}H NMR - 500 MHz, ^{13}C NMR - 125 MHz, solvent – DMSO-d$_6$)*

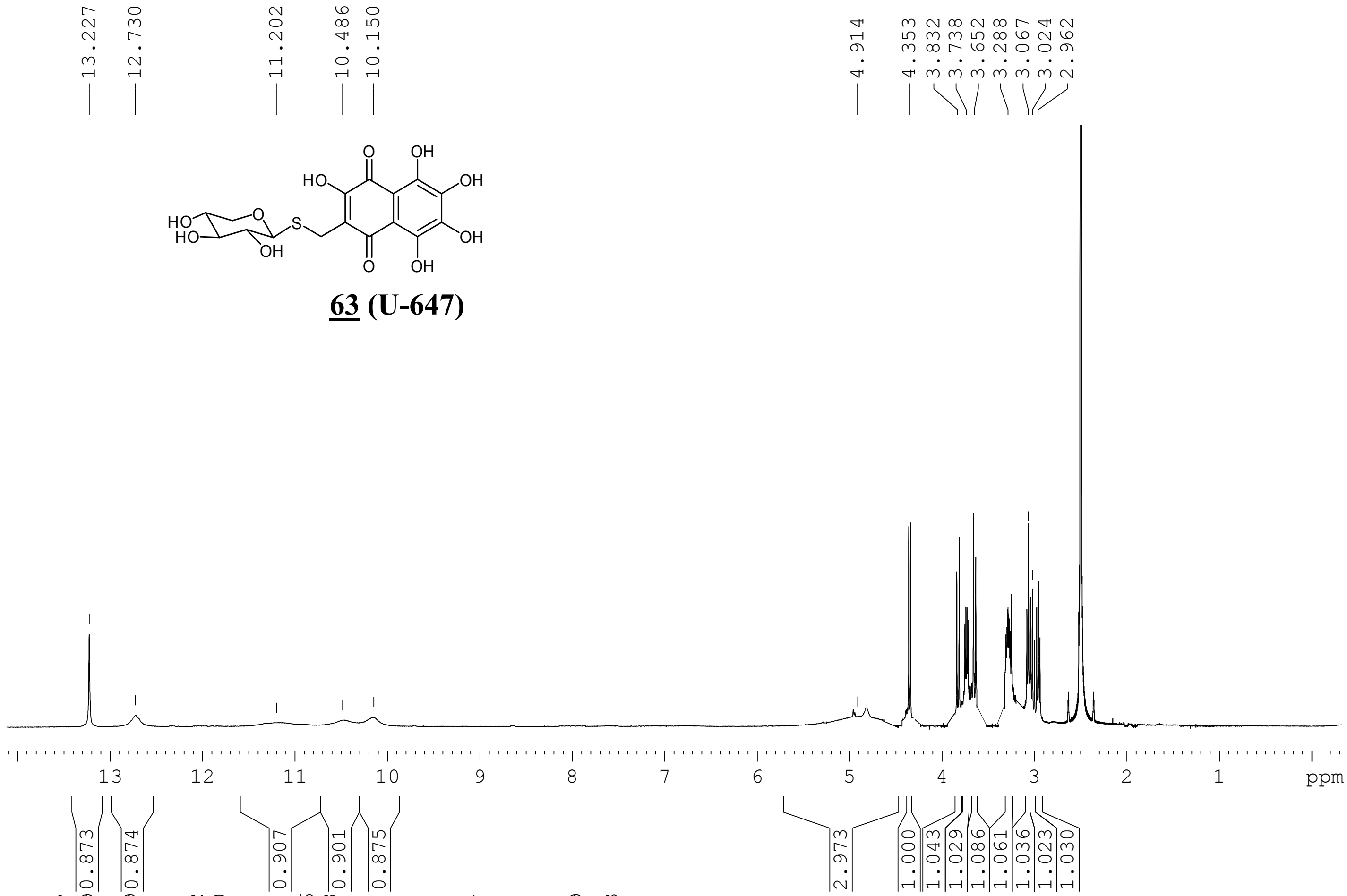

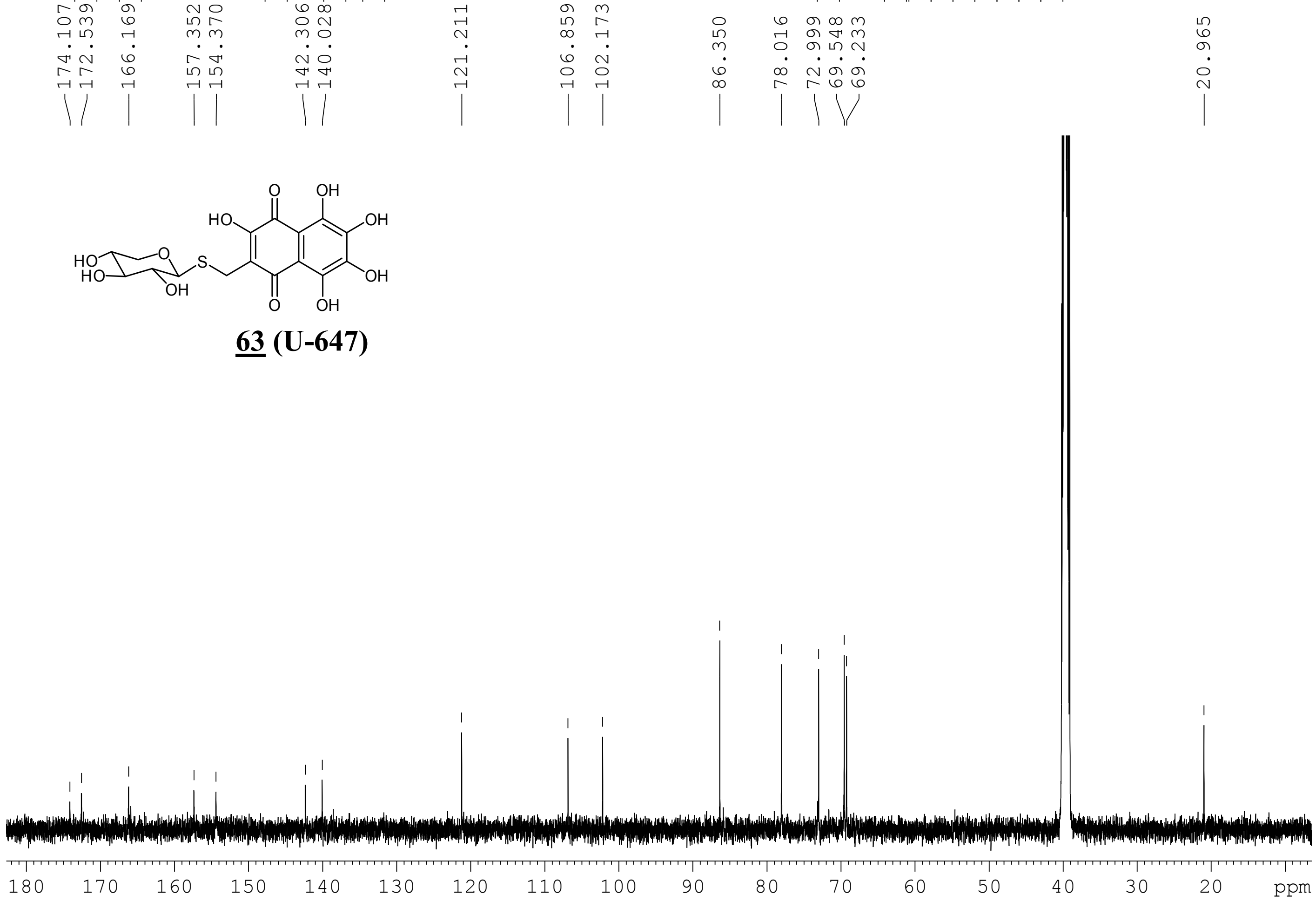

Supplement: Supplementary file 1 [file marinedrugs-18-00602-s001.pdf]
